# Stereochemical Analysis of Tertiary Trifluoroacetamides Leveraging Both Through-Space ^1^H···^19^F Spin–Spin Couplings and Anisotropic Solvent-Induced Shifts

**DOI:** 10.1021/acs.joc.5c02877

**Published:** 2026-02-24

**Authors:** Kizuki Watanabe, Ryota Takano, Hidetsugu Tabata, Kiriko Hirano, Motoo Iida, Tetsuta Oshitari, Hideaki Natsugari, Takenori Kusumi, Kayo Nakamura, Hideyo Takahashi

**Affiliations:** † Faculty of Pharmaceutical Sciences, Tokyo University of Science, 6-3-1 Niijuku, Katsushika-ku, Tokyo 125-8585, Japan; ‡ Faculty of Pharma Sciences, Teikyo University, 2-11-1 Kaga, Itabashi-ku, Tokyo 173-8605, Japan; § Bruker Japan K.K., 3-9 Moriya, Kanagawa-ku, Yokohama, Kanagawa 221-0022, Japan; ∥ Faculty of Pharmacy, Niigata University of Pharmacy and Medical and Life Sciences, 265-1 Higashijima, Akiha-ku, Niigata 956-8603, Japan; ⊥ Professor Emeritus, 13109 The University of Tokushima, 1−78−1 Shomachi, Tokushima 770−8505, Japan

## Abstract

In this study, the assignment of *E*/*Z*-isomers of *N*,*N*-dialkyl trifluoroacetamides (**1–8**) is investigated by using ^1^H-nuclear magnetic resonance spectroscopy, and data pertaining to both through-*space* spin–spin couplings (TSCs) and aromatic solvent-induced shifts (ASISs) are utilized to develop a reliable and convenient approach for determining the stereochemistry of these isomers. Although TSCs—observed when the F from the CF_3_ group is spatially close to protons—alone may be useful for determining *E*/*Z* isomers, through-*bond* couplings (TBCs) are also observed when the proton is five bonds apart from the F in the CF_3_ group. Thus, an additional one-dimensional ^1^H–^19^F heteronuclear Overhauser enhancement spectroscopy (HOESY) experiment is required to distinguish between the TSCs and TBCs. By contrast, the ASIS results for all compounds are consistent with the general observation that C_6_D_6_ preferentially shifts *trans*-methyl/methylene protons to the carbonyl oxygen atom over *cis*-methyl/methylene protons. Additionally, the ASISs with C_6_F_6_ of compounds **4–8** are analyzed to demonstrate the reliability of the ASIS-based method. Considering that ^1^H–^19^F HOESY experiments are somewhat specialized and uncommon, factoring both the TSCs and ASISs during the deduction process proves highly effective for determining the stereochemical assignment of *E*/*Z*-isomers of *N*,*N*-dialkyl trifluoroacetamides.

## Graphical abstract

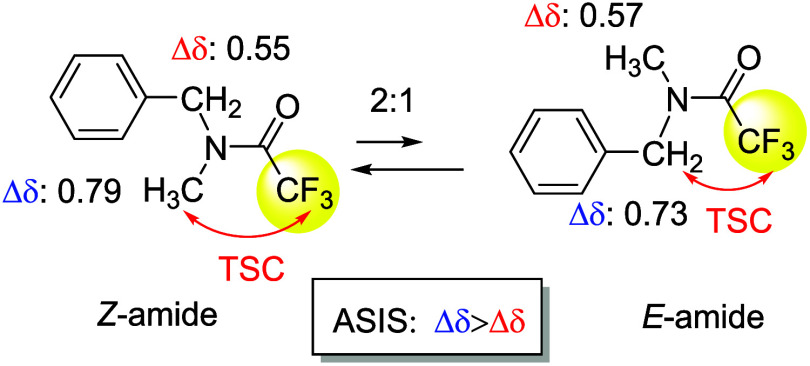

## Introduction

Recent studies have uncovered the remarkable chemical, physical, and biological properties of F,^1^ with more than 20% of currently manufactured drugs containing the F atom.^2^ Elucidating the steric and electronic effects of fluorine substituents in the molecular structures of drugs is therefore necessary for a rational drug design. In this context, we investigated the stereochemical structures of trifluoroacetamide compounds, *N*-acyl 5*H* dibenzo­[*b*,*d*]­azepin-7­(6*H*)-ones, expecting that the amide-derived *E*/Z conformations would significantly affect immunosuppressive activity by inhibiting the potassium channels (Kv1.3, IK-1) in T cells.^3^ To elucidate the steric and electronic effects of F substituents on the molecular structures of drugs, factoring the concept of through-*space* spin–spin couplings (TSCs)^4^ proves useful. TSCs are observed between two atoms when either possesses lone-pair electrons, and both are constrained at a distance smaller than the sum of their van der Waals radii. Two nuclei, such as ^19^F/^19^F, ^19^F/^1^H, or ^19^F/^13^C, can exchange spin information through space when in van der Waals contact, regardless of the number of chemical bonds separating them.^5^ Our previous research on the conformation of F-containing molecules factored the TSC principle to differentiate between *E*/*Z* isomers.^6^ Specifically, in that study,^6b^ the assignment of the ^1^H-nuclear magnetic resonance (NMR) signals of *N*,*N*-dimethyl trifluoroacetamide (DMTFA) **1** ^7^ was elucidated on the basis of the TSCs.

Conventionally, the relative chemical shifts of *N*-methyl groups in tertiary amides have often been rationalized in terms of the anisotropic effect of the carbonyl group, with the downfield-shifted methyl group frequently assigned as being *cis* to the amide carbonyl oxygen. However, in our previous study,^6b^ the TSC arising from CF_3_ was observed corresponding to the peak at a lower chemical shift, indicating that this methyl group was located on the side opposite to the amide carbonyl group. On the basis of the ^1^H–^19^F heteronuclear Overhauser spectroscopy (HOESY) experiment^8^ for **1**, we concluded that the signal at a lower magnetic field (3.14 ppm) corresponded to the *trans*-methyl group, and it followed that the signal at the upper magnetic field (3.05 ppm) corresponded to the *cis*-methyl group (Figure 1). However, at that stage, we did not consider the possibility that not only TSCs but also through-*bond* couplings (TBCs) could contribute to the observed interactions. It was subsequently recognized that the coupling constant (^5^ *J*_HF_) of *cis*-methyl/*trans-*methyl in compound **1** had not been adequately verified.

**Figure 1:**
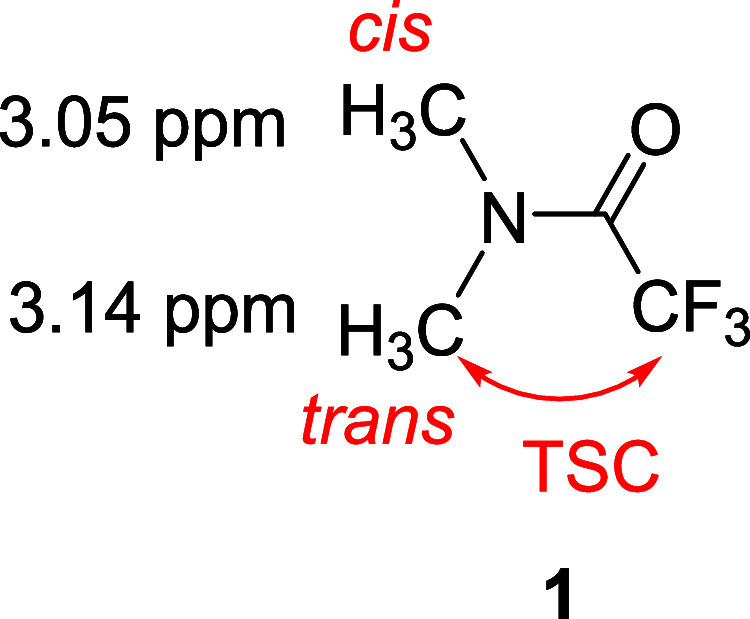
*N*,*N*-Dimethyl trifluoroacetamide **1**.

These studies prompted us to examine the stereochemical assignment of *N*-alkyl protons of *N*,*N*-disubstituted trifluoroacetamides on the basis of the TSCs. In this study, ^1^H–^19^F HOESY experiments and aromatic solvent-induced shifts (ASISs)^9^ are leveraged to complement the TSC results, as extra verifications are needed to differentiate TSCs and TBCs that arise similar to TSCs. Despite the fact that these HOESY experiments are reliable, they are not commonly performed. By contrast, the ASIS-based method is a fundamental and useful method that has been utilized for over 50 years. This method is based on the well-known observation that the chemical shifts in the NMR spectra of organic molecules change depending on the solvent used. In an anisotropic solvent (e.g., C_6_D_6_), the NMR signals shift upfield when compared to their shifts in an isotropic solvent (e.g., CDCl_3_). Such chemical shifts caused by solvents possessing different magnetic properties are defined as ASISs (Δδ): Δδ = δ_S_ – δ_AS_, where δ_S_ is the position of the signal of an H atom in an isotropic solvent (e.g., CDCl_3_) and δ_AS_ is the corresponding value for the same signal in an anisotropic solvent (e.g., C_6_D_6_). Considering the ASIS (Δδ) observed in ^1^H NMR experiments, the position of the H atoms relative to the carbonyl group was predicted, which is known as the carbonyl planar rule.^10^ Based on this rule, *cis*/*trans*-methyl or methylene was assigned to the carbonyl group of the tertiary acetamides based on the value of Δδ.^11^

## Results and Discussion

Initially, the assignment of the ^1^H NMR signals of *N*,*N*-dimethyl trifluoroacetamide (DMTFA) **1** in CDCl_3_ was reexamined. Although the assignment of each methyl peak was ultimately determined, the question remained as to why a slight doublet coupling was observed for the *cis*-methyl peak (Figure 2(a)). Therefore, these peaks were reexamined more closely.

**Figure 2:**
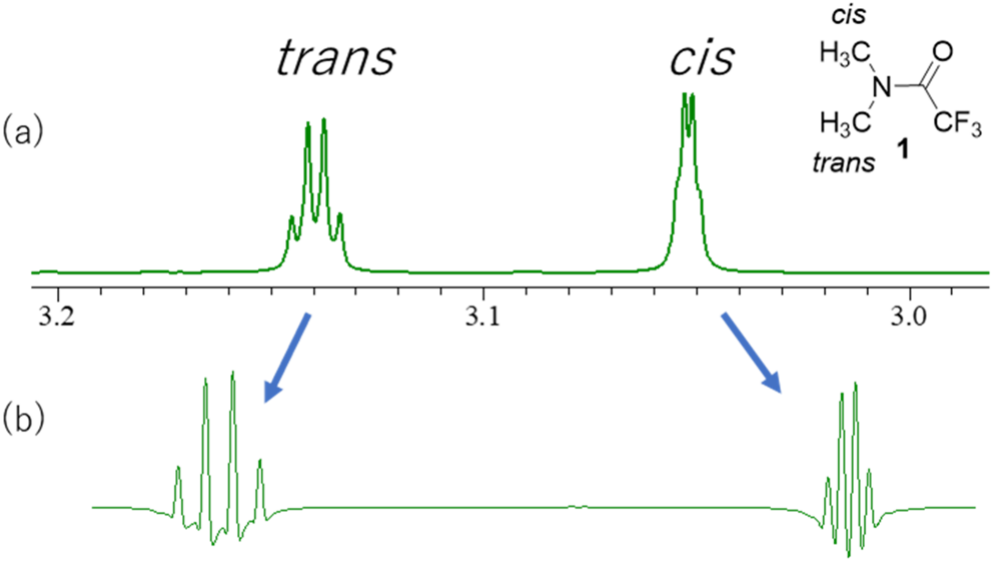
(a) ^1^H NMR spectrum of **1** (400 MHz, CDCl_3_) and (b) ^1^H NMR spectrum of **1** (400 MHz, CDCl_3_) after application of the sine bell window function.

The peak of the methyl group *trans* to the amide carbonyl group was observed as a broad signal. Upon application of a sine bell window function, a commonly applied NMR data-processing technique to enhance spectral resolution, both methyl peaks appeared as quartets, indicating the presence of ^1^H–^19^F couplings (Figure 2(b)). The coupling constant of *cis*-methyl (^5^ *J*_HF_ = 0.8 Hz) is smaller than that of *trans-*methyl (^5^ *J*_HF_ = 1.6 Hz). Considering that both protons were five bonds apart from the F in the CF_3_ group, TBCs were observed. Additionally, to distinguish TSCs from TBCs, **1** was subjected to ^1^H–^19^F HOESY experiments (Figure 3).

**Figure 3:**
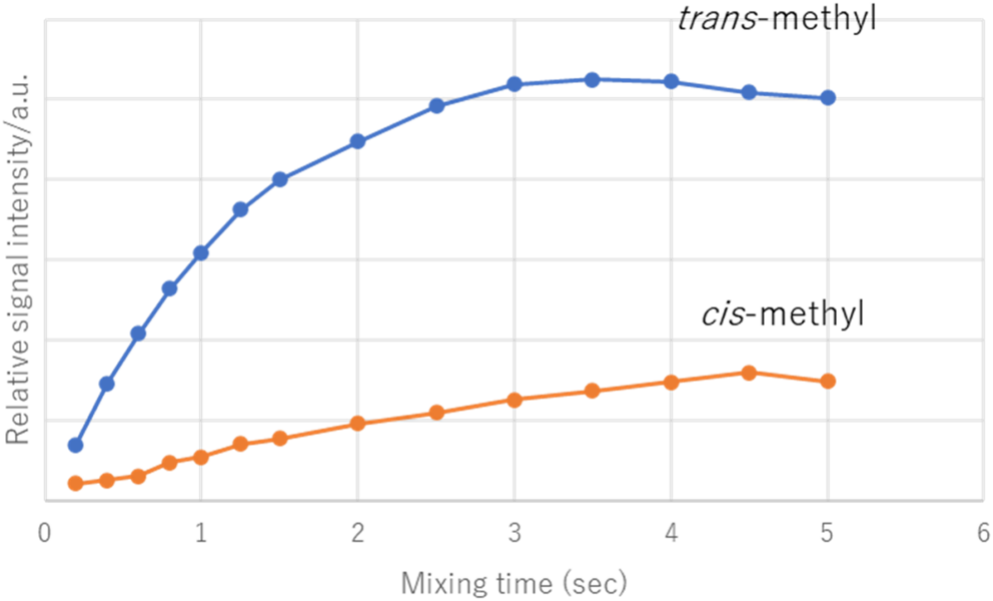
^1^H–^19^F HOESY built-up curves (irradiation at – 69.573 ppm: ^19^F signal) (600/564 MHz, CDCl_3_) of **1**.

In Figure 3, *trans*-methyl (3.14 ppm), which has a steeper slope than that of *cis*-methyl (3.05 ppm), is spatially closer to CF_3_.^12^ This result confirms that *trans*-methyl exhibits TSCs, *cis*-methyl exhibits TBCs, and the coupling constant of TSC (^5^ *J*_HF_ = 1.6 Hz) is larger than that of TBC (^5^ *J*_HF_ = 0.8 Hz). This suggests that the ^5^ *J*_HF_ coupling depends on the spatial distance dependence. To clarify this point, DFT calculations were performed to determine the most stable conformation of compound **1**, and the distances between the F atoms of CF_3_ and the hydrogen atoms of the *cis*- and *trans*-methyl groups were calculated (Supporting Information Figure S23). The H···F distance between *cis*-methyl H and CF_3_ F was 4.543 Å, and the H···F distance between *trans*-methyl H and CF_3_ F was 2.431 Å. These results indicate that the *trans*-methyl group, which is closer to the F atom, exhibits stronger TSC coupling. Notably, the peak of *cis*-methyl, which is located close to the carbonyl oxygen, appears corresponding to the *upper* magnetic field as opposed to that of *trans*-methyl.

As part of our further investigations of the ASIS on **1**, *cis*-methyl and *trans*-methyl peaks were observed in C_6_D_6_ at 2.24 and 2.12 ppm (Supporting Information Figure S1), with ASIS (Δδ) values of 0.81 and 1.02, respectively. Evidently, the ASIS (Δδ) value of *trans*-methyl is larger than that of *cis*-methyl (Figure 4). This result is consistent with the carbonyl planar rule, which states that anisotropic solvent shifts of methyl protons *trans* to the carbonyl oxygen atom are larger than those oriented *cis* to the oxygen.

**Figure 4:**
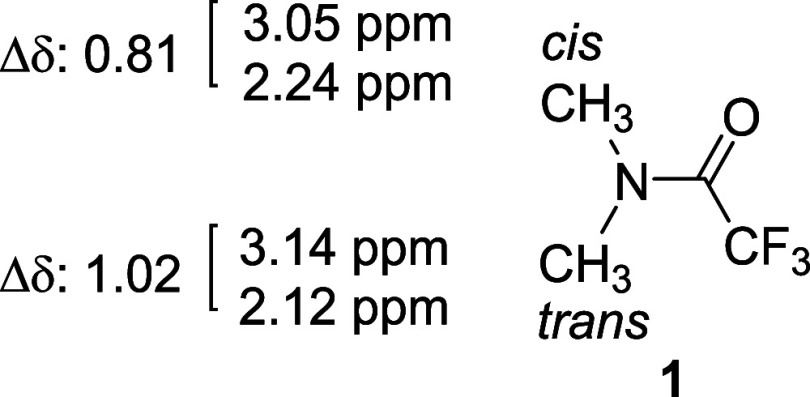
ASIS (Δδ) of the methyl groups of **1**. Chemical shifts: up in CDCl_3_, down in C_6_D_6_ Δδ values: 0.81 (*cis*), 1.02 (*trans*).

Subsequently, we investigated the ^1^H NMR spectra of *N*,*N*-diethyl trifluoroacetamide **2**.^13^ However, the methylene proton signals of *cis*/*trans* ethyl groups in CDCl_3_ could not be distinguished because of overlap, although the methyl signals were distinctly separated (Supporting Information Figure S3). The methylene protons of the *cis*/*trans* ethyl groups in C_6_D_6_ were well separated (Supporting Information Figure S5), and applying a sine bell window function enabled the calculation of the Δδ values (Figure 5).

**Figure 5:**
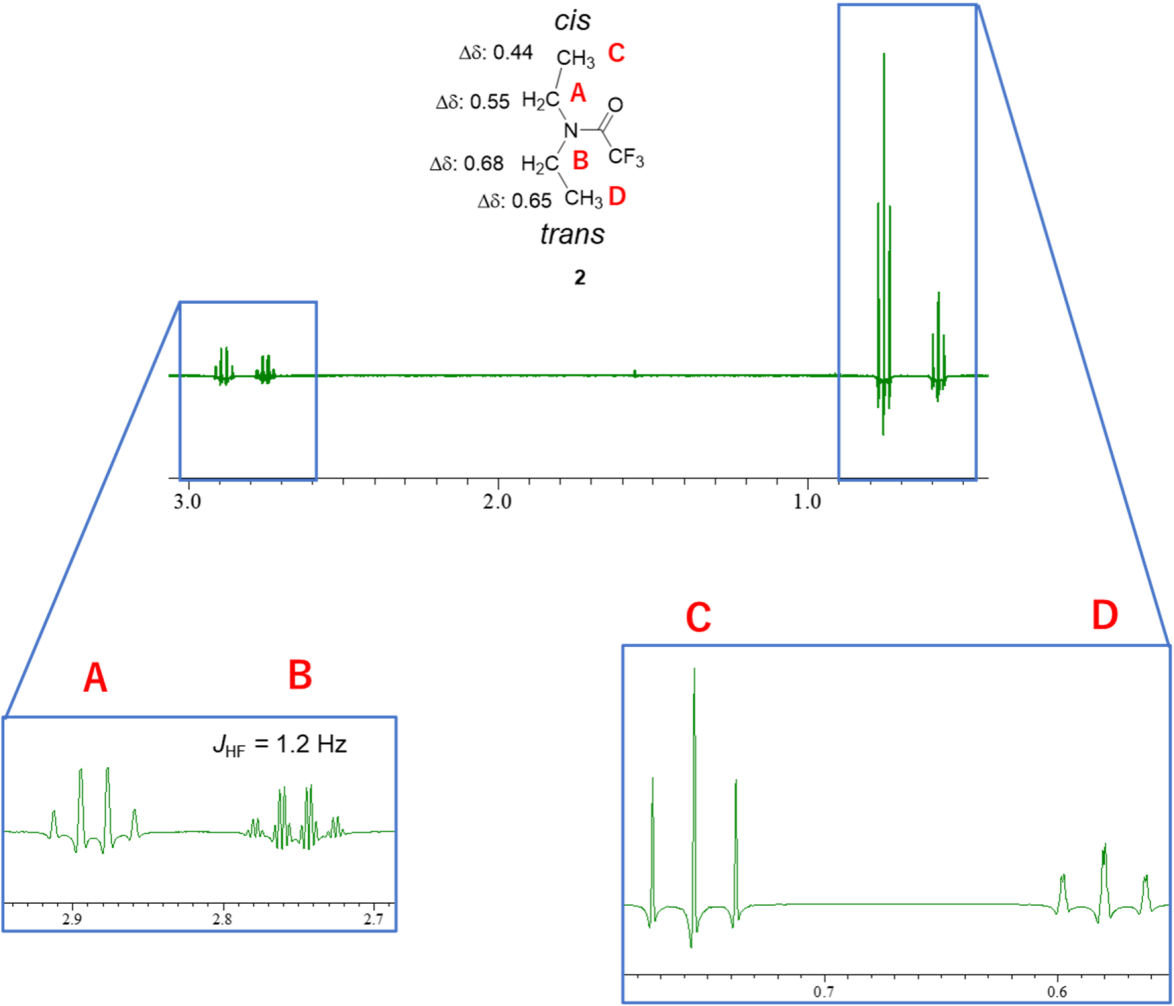
^1^H NMR spectrum of **2** (400 MHz, C_6_D_6_) after sine bell window function processing. Δδ values: 0.55 (A), 0.68 (B), 0.44 (C), 0.65 (D).

Signal (**A**) (2.89 ppm) was observed as a simple quartet with a lower chemical shift than signal (**B**) (2.76 ppm), which was observed as a quartet of quartets. These signals correspond to the methylene protons of the ethyl groups. Signal (**A**) was split into a simple quartet owing to coupling with the adjacent methyl group, and no TSC/TBC with the F of CF_3_ was observed. By contrast, the signal (**B**) was observed as a quartet of quartets (^5^ *J*_HF_ = 1.2 Hz) because of the TSC from CF_3_. For the methyl protons, signal (**C**) (0.76 ppm) was observed as a simple triplet with a chemical shift lower than that of signal (**D**) (0.59 ppm), which appeared as a doublet of triplets. Because the methyl protons are six bonds from the F of the CF_3_ group, a TBC between them would be impossible. Given this consideration, the upperfield methyl signal (**D**) was assumed to be that of the *trans*-methyl group, which is close to CF_3_, with a small TSC.

The origin of the small couplings observed in signals (**B**) and (**D**) was further verified via HOESY spectroscopy; the slope of (**B**) was observed to be the steepest, followed by those of (**D**), (**A**), and (**C**) (Figure 6). From these results, it was concluded that the small couplings of signals (**B**) and (**D**) were due to TSC; thus, signal (**B**) at the upper magnetic field corresponded to the *trans*-methylene group, and signal (**A**) at the lower magnetic field corresponded to the *cis*-methylene group. Similarly, the signal (**D**) at the upper magnetic field corresponded to the *trans*-methyl group and the signal (**C**) at the lower magnetic field corresponded to the *cis*-methyl group.

**Figure 6:**
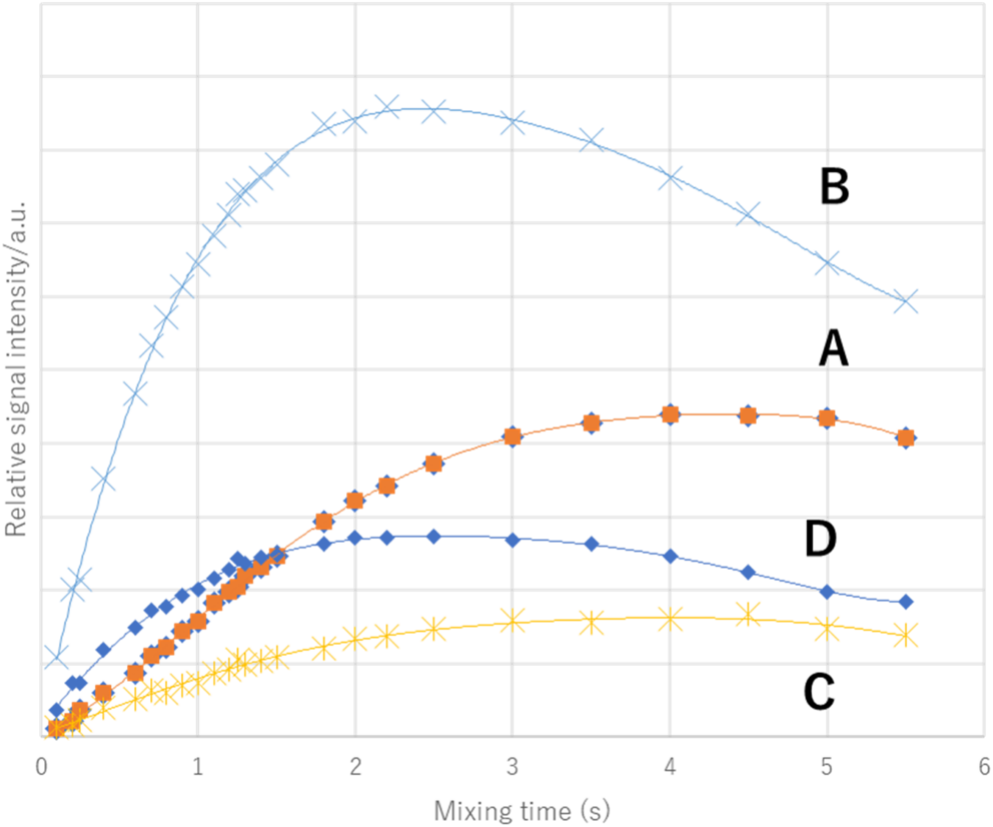
^1^H–^19^F HOESY built-up curves (irradiation at –69.377 ppm: ^19^F signal) (600/564 MHz, C_6_D_6_) of **2**.

Contrary to the case of *N*,*N*-dimethyl trifluoroacetamide **1**, signals (**B**) and (**D**), which were *trans* to the carbonyl oxygen, were observed at a magnetic field higher than those *cis* to the oxygen. Notably, however, the spectrum of **2** in Figure 5 was obtained using C_6_D_6_ as the solvent.

Next, we investigated ASIS on **2**. For the *cis*-methylene group (**A**), the ASIS (Δδ) was 0.55, and that of the *trans*-methylene group (**B**) was 0.68, indicating a larger ASIS (Δδ) value of the *trans*-methylene group than that of the *cis*-methylene group. Similarly, the ASIS (Δδ) value of the *cis*-methyl group was 0.44, and that of the *trans*-methyl group was 0.65, indicating a larger ASIS (Δδ) value of the *trans*-methyl group than that of the *cis*-methyl group (Figure 5). This result is consistent with the carbonyl planar rule, which states that the anisotropic solvent shifts of the protons *trans* to the carbonyl oxygen atom are greater than those of the *cis* protons.

Notably, in the ^13^C­{^1^H} NMR spectrum (and contrary to the ^1^H NMR spectrum), each peak of the *trans*-methylene/methyl group, assigned based on the heteronuclear single quantum coherence (HSQC) spectrum (Supporting Information Figure S7), appeared at a magnetic field lower than that for peaks shown by the *cis*-methylene/methyl group (Figure 7). Furthermore, the signal of *trans*-methylene was observed as a broad quartet (^4^ *J*_CF_ = 3.4 Hz) considering the TSC between C^13^ and F.

**Figure 7:**
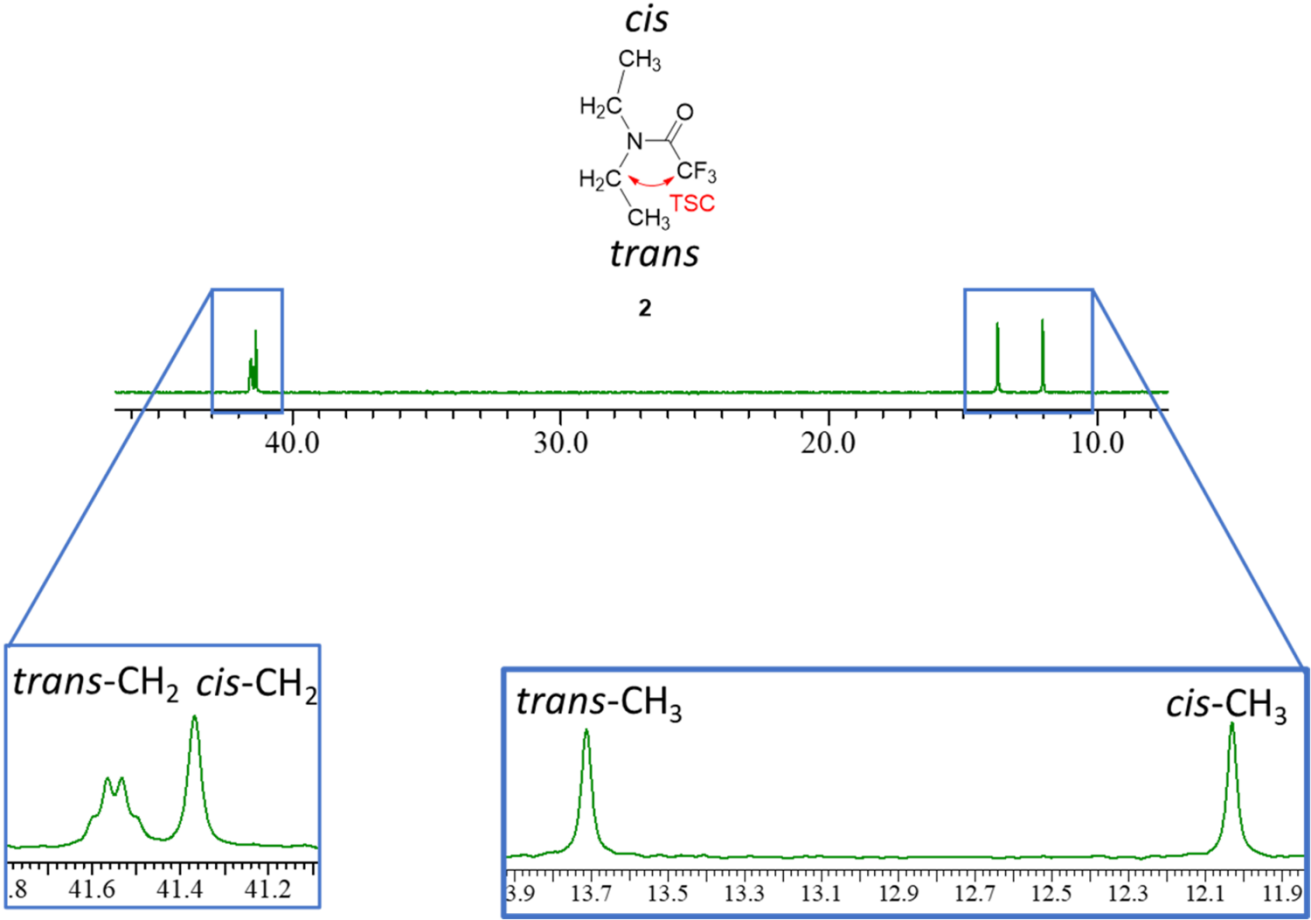
^13^C­{^1^H} NMR spectrum of **2** (100 MHz, C_6_D_6_).

Additionally, the assignment of the ^1^H NMR signals of *N*-benzyl-*N*-methyl trifluoroacetamide **3** ^14^ in CDCl_3_ was examined. In this case, compound **3** was observed to exist as an equilibrium mixture of *E*/*Z* amide rotamers (**3**-**I** and **3**-**II**) in a 2:1 ratio (Figure 8(a)). Moreover, the benzyl methylene protons of both *E*/*Z* amide rotamers were observed as broad signals. On the contrary, of the signals corresponding to the methyl groups, the one at a lower magnetic field was observed as a clear quartet, and the other appeared as a slightly broad signal. Upon application of the sine bell window function, signals (**A)**, which corresponded to the methylene group of **3**-**I**, and signal (**B)**, which corresponded to the methylene group of **3**-**II**, remained broad. However, signal (**C)**, which corresponded to the methyl group of the major isomer (**3**-**I**), and signal **D**, which corresponded to the minor isomer (**3**-**II**), were observed as well-defined quartets (Figure S8).

**Figure 8:**
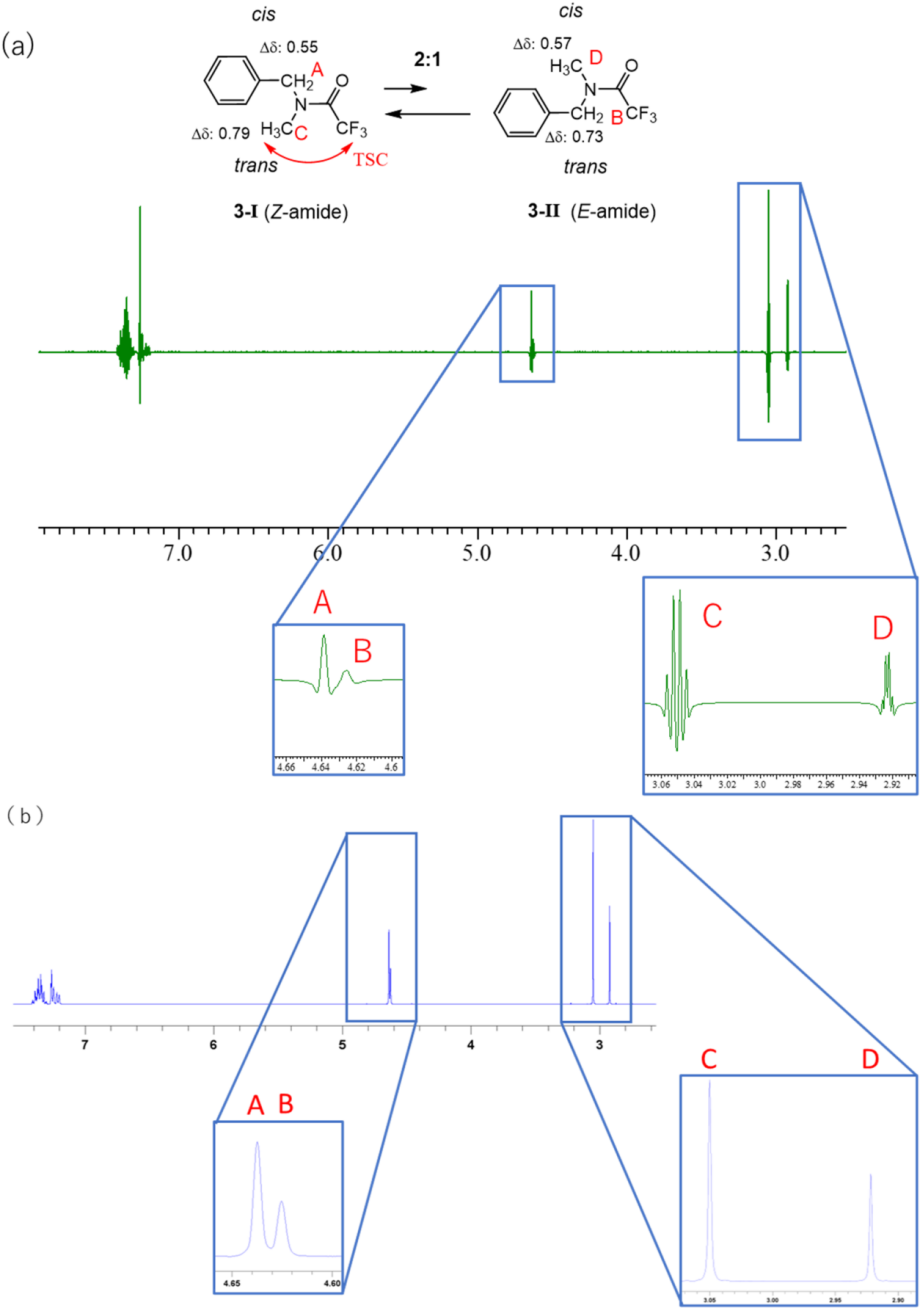
(a) ^1^H NMR spectrum of **3** (400 MHz, CDCl_3_). (b) ^19^F-decoupled ^1^H NMR spectra of **3** (400 MHz, CDCl_3_). Δδ values: 0.55 (A), 0.73 (B), 0.79 (C), 0.57 (D).

To confirm that the splitting of signals **C** and **D** was due to the F atoms, ^19^F-decoupled ^1^H NMR experiments were performed. As shown in Figure 8(b), irradiation of ^19^F resulted in the simplification of their signal patterns to singlets; moreover, these experiments facilitated the calculation of the H–F coupling constants of signals **C** and **D** as 1.6 and 0.8 Hz, respectively. To confirm whether the couplings were due to TSC or TBC, we performed one-dimensional (1D) HOESY ^1^H–^19^F experiments again.

Figure 9(a) shows the ^1^H–^19^F HOESY built-up curves of **3**-**I** observed in CDCl_3_; the slope of **C** (methyl group) is evidently steeper than that of **A** (methylene group). Therefore, it is clear that the coupling of signal **C** is due to TSC. Thus, the major isomer of **3**-**I** is *Z*-amide, in which the methyl group is close to CF_3_. Similarly, Figure 9(b) shows that the slope of **B** (methylene group) is steeper than that of **D** (methyl group); thus, the minor isomer **3**-**II** is *E*-amide, in which the methylene group is close to CF_3_. To improve the reliability of these assignments, we examined the proton-decoupled ^13^C­{^1^H} NMR spectrum of **3** (Supporting Information Figure S9), in which the signals were assigned according to the HSQC spectrum of **3** (Supporting Information Figure S10). The CH_2_ of **3**-**II** (52.9 ppm) and CH_3_ of **3**-**I** (34.2 ppm) were observed as broad quartets, whereas the CH_2_ of **3**-**I** (52.3 ppm) and CH_3_ of **3**-**II** (34.0 ppm) were observed as singlets. These results confirmed that each coupling observed in CH_2_ of **3**-**II** and CH_3_ of **3**-**I** was caused by TSC.

**Figure 9:**
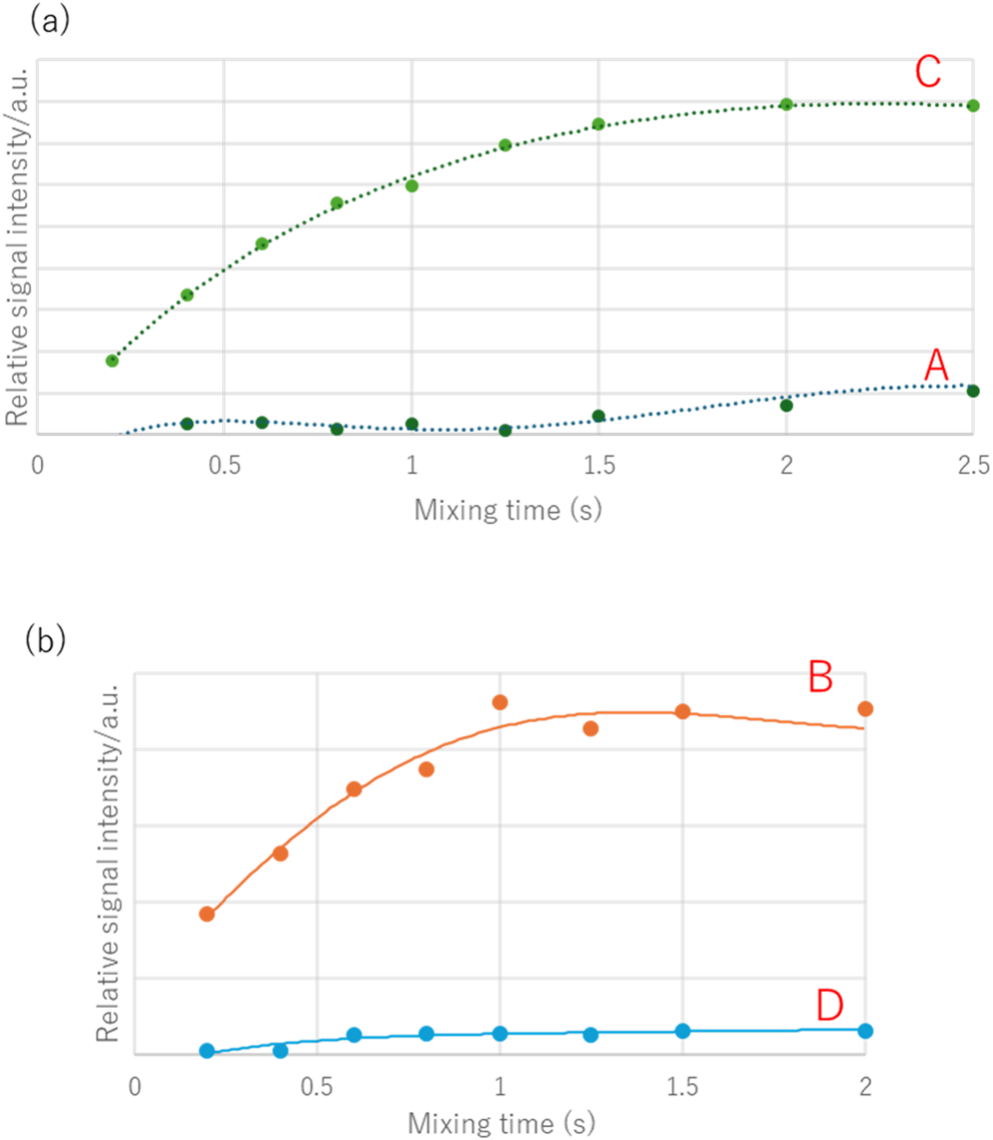
^1^H–^19^F HOESY built-up curves of **3**. (a) Irradiation of major isomers **3**-**I** at –69.52 ppm (^19^F). (b) Irradiation of minor isomers **3**-**II** (600/564 MHz, CDCl_3_) at –67.95 ppm (^19^F).

Subsequently, we investigated the ASISs on **3**-**I** and **3**-**II (** Figure 8(a)). The ^1^H NMR spectrum of **3** in C_6_D_6_ is shown in the Supporting Information (Figure S11). For **3**-**I**, the value of ASIS (Δδ) of the methylene group was 0.55, and that of the methyl group was 0.79, indicating that the methyl *trans* to the amide carbonyl group had a larger ASIS (Δδ) value than that of the methylene group *cis* to the amide carbonyl group. Similarly, for **3**-**II**, the ASIS (Δδ) value of the methylene group (0.73) was larger than that of the methyl group (0.57). Thus, the ASIS (Δδ) value of the methylene protons *trans* to the amide carbonyl group was larger than that of the methyl group *cis* to the amide carbonyl group. This result was consistent with the carbonyl planar rule (*vide supra*).

The results of ^1^H NMR experiments on the tertiary trifluoroacetamide derivatives (**1**–**3**) are summarized as follows: (1) In compound **1** in CDCl_3_, TSC was observed at the *trans*-methyl group close to the trifluoromethyl group, which was observed at a lower magnetic field, and the peak of the *cis*-methyl was observed at the upper magnetic field. (2) In compound **2** in C_6_D_6_, TSC was observed at the *trans*-methyl and *trans*-methylene groups close to the trifluoromethyl group; however, each peak was observed at a magnetic field higher than those of the peaks of the *cis*-methyl and methylene. (3) In CDCl_3_, between the methyl groups of **3**-**I** and **3**-**II**, the *trans*-methyl group of **3**-**I**, which exhibited TSC owing to the trifluoromethyl group, was observed at a lower magnetic field than that of the *cis*-methyl group of **3**-**II**. By contrast, the *cis*-methylene group of **3**-**I** was observed at a magnetic field lower than that of the *trans*-methylene group of **3**-**II**. Considering the above results (1) and (3), for some cases of tertiary trifluoroacetamide derivatives, such as compound **1** in CDCl_3_, the *trans*-methyl was observed at a lower magnetic field than that of the *cis*-methyl; in other cases, such as for compound **2** in C_6_D_6_, the *trans*-methyl and *trans*-methylene were observed at a higher field than that of the *cis-*methyl and *cis-*methylene, respectively. Overall, the assignment of the ^1^H NMR signals of *N*,*N*-dialkyl trifluoroacetamide and the distinction of *cis*/*trans* rotamers based on the conventional anisotropic effect of the carbonyl group are not reliable. In contrast, the TSC is based on spatial distance and therefore appears to be useful to consider. However, TBC should also be factored if the protons are five bonds apart from F in the CF_3_ group. As we have shown, the HOESY measurements can distinguish between TBC and TSC; however, these experiments are somewhat sophisticated and therefore less commonly utilized. When both TSC and TBC were observed, such as in the *cis*/*trans*-methyl groups of compound **1** (Figure 2(b)) and compound **3** (Figure 8(a)), the coupling constant of TSC being larger than that of TBC should aid the differentiation between TSC and TBC. Additionally, examining the classical ASIS proves helpful. For compounds **1**–**3**, the ASIS-based assignment was reliable, consistent with the general carbonyl planar rule (*vide supra*).

Therefore, we decided to distinguish between *cis*/*trans* rotamers of trifluoroacetamide derivatives **4–8** using two methods: (1) Classical ASIS and (2) comparison of the coupling constants derived from the TSC and TBC. First, the NMR spectra of CDCl_3_ and C_6_D_6_ were measured for each compound (Figures S13–S22) to determine ASIS. For all the *cis*/*trans* rotamers of compounds **4–8** (*N*-Me and *N*-CH_2_−), the ASIS (Δδ) value of the methyl/methylene group *trans* to the amide carbonyl group was expected to be larger than that of the group *cis* to the amide carbonyl group. Given this expectation, the *cis* and *trans* isomers were estimated by comparing the ASIS (Δδ) values for the protons (α/α’), which were five bonds apart from F in the CF_3_ group (Figure 10(a), Table S1). *N*-Methyl-*N*-propyl trifluoroacetamide **4**,^15^ *N*-ethyl-*N*-benzyl trifluoroacetamide **5**,^16^ *N*-benzyl-*N*-propyl trifluoroacetamide **6**,^17^ *N*-ethyl-*N*-propyl trifluoroacetamide **7**,^18^ and *N*-allyl-*N*-methyl trifluoroacetamide **8** ^19^ in C_6_D_6_/CDCl_3_ exist as equilibrium mixtures of *cis*/*trans* rotamers (**4-I**/**4-II**, **5-I**/**5-II**, **6-I**/**6-II**, **7-I**/**7-II**, and **8-I**/**8-II**) in various ratios.

**Figure 10:**
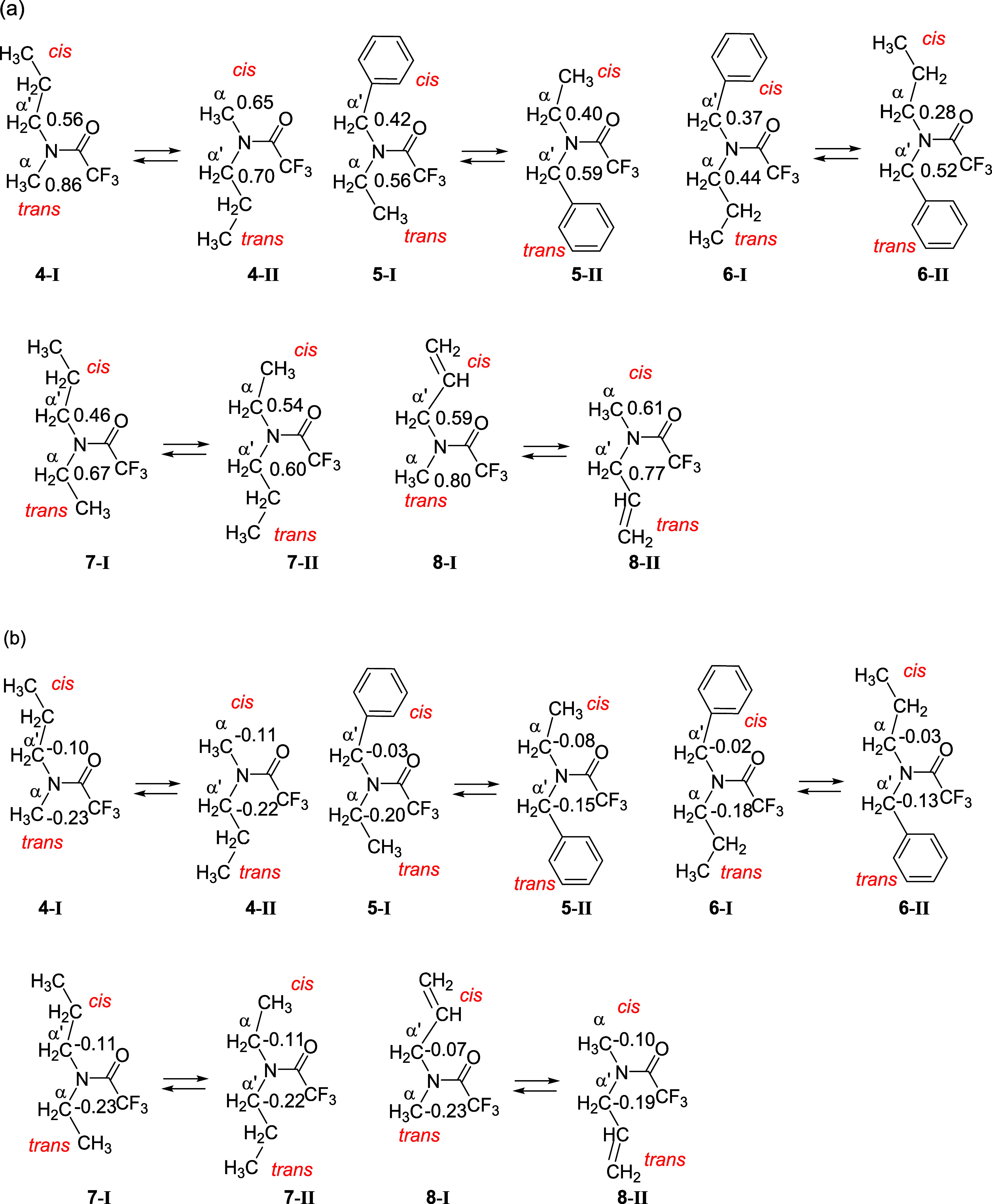
(a) ASIS (CDCl_3_/C_6_D_6_) spectrum of compounds **4–8**. Δδ values for **4**-**I**: 0.86 (α), 0.56 (α′), Δδ values for **4**-**II**: 0.65 (α), 0.70 (α′), Δδ values for **5**-**I**: 0.56 (α), 0.42 (α′), Δδ values for **5**-**II**: 0.40 (α), 0.59 (α′), Δδ values for **6**-**I**: 0.44 (α), 0.37 (α′), Δδ values for **6**-**II**: 0.28 (α), 0.52 (α′), Δδ values for **7**-**I**: 0.67 (α), 0.46 (α′), Δδ values for **7**-**II**: 0.54 (α), 0.60 (α′), Δδ values for **8**-**I**: 0.80 (α), 0.59 (α′), Δδ values for **8**-**II**: 0.61 (α), 0.77 (α′). (b) ASIS (CDCl_3_/C_6_F_6_) of compounds **4–8**. Δδ values for **4**-**I**: −0.23 (α), −0.10 (α′), Δδ values for **4**-**II**: −0.11 (α), −0.22 (α′), Δδ values for **5**-**I**: −0.20 (α), −0.03 (α′), Δδ values for **5**-**II**: −0.08 (α), −0.15 (α′), Δδ values for **6**-**I**: −0.18 (α), −0.02 (α′), Δδ values for **6**-**II**: −0.03 (α), −0.13 (α′), Δδ values for **7**-**I**: −0.23 (α), −0.11 (α′), Δδ values for **7**-**II**: −0.11 (α), −0.22 (α′), Δδ values for **8**-**I**: −0.23 (α), −0.07 (α′), Δδ values for **8**-**II**: −0.10 (α), −0.19 (α′).

For each isomer, the proton exhibiting the larger ASIS (Δδ) value was assigned a *trans* configuration to the amide carbonyl group. This assignment allowed us to estimate the *cis*/*trans* ratios of the rotamers of **4–8**. To further verify this, the coupling constant of ^1^H–^19^F coupling in C_6_D_6_ was determined for the protons (α/α′), which are five bonds apart from the F in CF_3_ (Table 1). In compound **4**-**II**, the α′-protons closer to the trifluoromethyl group—which is *trans*-methylene to the amide carbonyl group—had a larger coupling constant (1.2 Hz) than that of the α-protons (0.4 Hz). Similarly, in compounds **8**-**II**, the α′-protons, *trans*-methylene to the amide carbonyl group, closer to the trifluoromethyl group, showed a larger coupling constant (1.2 Hz) than that of the α-protons (0.8 Hz). For all other isomers except compounds **5**-**II** and **6**-**II**, ^1^H–^19^F couplings (0.8–1.6 Hz) derived from TSC were observed for the protons spatially close to the trifluoromethyl group but not for the protons *cis* to the amide carbonyl group; this implies that no TBC was observed in these protons either. These results are consistent with the fact that the coupling constant of TSC is larger than that of TBC. For compounds **5**-**II** and **6**-**II**, in which TSC-derived ^5^ *J*_HF_ couplings were not observed, stable conformations were determined by DFT calculations, and the distances between the F atom of the trifluoromethyl group and the H atom at the benzyl position were calculated (Supporting Information Figures S24–S25). As a result, the distances between the F atom and the H atom at the benzyl position in compounds **5**-**II** and **6**-**II** were found to be almost the same as the distance between the F atom of the trifluoromethyl group and the H atom of the methyl group in compound **1**. Therefore, in this case, TSCs may not be observed due to the influence of the electronic effects of the benzene ring. Further consideration of this point is required.

**Table 1:** ^1^H–^19^F Coupling Constants of Compounds **4–8** in C_6_D_6_

	^1^H–^19^F coupling constants (Hz)	
compound	*trans*	*cis*
**4-I**	α: 1.6	α′: not observed
**4-II**	α′: 1.2	α: 0.4
**5-I**	α: 1.2	α′: not observed
**5-II**	α′: not observed	α: not observed
**6-I**	α: 0.8	α′: not observed
**6-II**	α′: not observed	α: not observed
**7-I**	α: 1.2	α′: not observed
**7-II**	α′: 1.2	α: not observed
**8-I**	α: 1.6	α′: not observed
**8-II**	α′: 1.2	α: 0.8

Furthermore, the ASIS was calculated by considering C_6_F_6_ instead of C_6_D_6_. Protons at the positive end of the dipole are shielded in C_6_D_6_ and deshielded in C_6_F_6_, whereas the opposite occurs for protons at the negative end. Consequently, the direction of the C_6_F_6_-induced shift is opposite that of the C_6_D_6_-induced shift. The carbonyl planar rule also reportedly holds true for ASIS with both C_6_F_6_ and C_6_D_6_, although the value of ASIS (Δδ) in this case: Δδ = δ_CDCl3_ – δ_C6F6_ is negative.^20^ The ASIS values of the α- and α′-protons of the compounds **4–8** exhibit a general trend, i.e., the ASIS phenomena are reversed for the C_6_F_6_ effects (Figure 10(b)). Additionally, the ASIS values of all of the protons of compounds **1–8** also show a general trend for the C_6_F_6_ effects (Supporting Information Table S2). Ultimately, as mentioned earlier, the configuration of *cis*/*trans* rotamers of trifluoroacetamide derivatives **4**–**8** was determined based on TSC in addition to ASIS.

## Conclusion

For the assignment of *N*,*N*-dialkyl trifluoroacetamides (**1**–**3**), a conventional method based on the anisotropic effect of the carbonyl group was proven to be unreliable. Therefore, a combination of TSC and ASIS for determining the assignment was investigated in this study. Notably, TSC and TBC could be observed for protons that were five bonds apart from F in the CF_3_ group. In such cases, the observed ^1^H–^19^F coupling constants for TSC being larger than those of TBC proved to be useful for the analyses, and the additional 1D ^1^H–^19^F HOESY experiments provided solid evidence for verification. We confirmed that for compounds **2–8**, the observed TSCs were always larger than the TBCs, and the additional 1D ^1^H–^19^F HOESY experiments provided further evidence to verify this observation. Additionally, the ASIS (CDCl_3_/C_6_D_6_) in compounds **1 −3** supported the assignments deduced from the TSC. These experiments clarified that methyl/methylenes, which are *trans* to the amide carbonyl groups, have larger ASIS (Δδ) values than those *cis* to the amide carbonyl groups. The stereochemistry of the *cis*/*trans* rotamers of trifluoroacetamide derivatives **4–8** was also verified by considering the ASIS (CDCl_3_/C_6_D_6_). Additionally, the ASIS (CDCl_3_/C_6_F_6_) of compounds **4–8** also supported the aforementioned conclusion.

For the assignments of *N*,*N*-dialkyl trifluoroacetamides, the TSC supported by ^1^H–^19^F HOESY experiments proved to be reliable, although the HOESY experiments require specialized measurements. In contrast, TSC and ASIS can be more easily obtained using conventional 1D techniques. However, there are cases where TSC is not observed; in such cases, it will be necessary to verify with a larger number of compounds if it is acceptable to determine the stereochemistry of *N*,*N*-dialkyl trifluoroacetamides using ASIS alone. Despite the phenomena of both TSC and ASIS being known for a long time, they have not been exploited significantly in recent years. Our method, which combines TSC and ASIS, is therefore expected to be applicable to the determination of *cis*/*trans* configurations in other tertiary trifluoroacetamides. Improvements to this method will be reported in the future.

## Experimental Section

### Materials

Compounds **1**–**8** were purchased and used.

### General Methods

The NMR spectra were recorded on a spectrometer at 400 MHz for ^1^H NMR, and at 100 MHz for ^13^C­{^1^H} NMR; additionally, ^19^F NMR spectra were recorded at 564 MHz. The chemical shifts were expressed in parts per million (ppm) downfield from tetramethylsilane as the internal standard, and the coupling constants (*J*) were reported in hertz (Hz). The splitting patterns are abbreviated herein as follows: singlet (s), doublet (d), triplet (t), quartet (q), sextet (sext), doublet of quartet (dq), triplet of quartet (tq), quartet of quartet (qq), and multiplet (m). The structural assignments were performed using additional information obtained from correlation spectroscopy (COSY), nuclear Overhauser effect spectroscopy (NOESY), and HSQC experiments. NMR measurements were performed by dissolving 3 mg of the substrate in 0.6 mL of a deuterated solvent at 23 °C.

### *N*,*N*-Dimethyltrifluoroacetamide (**1**)^6b,7^

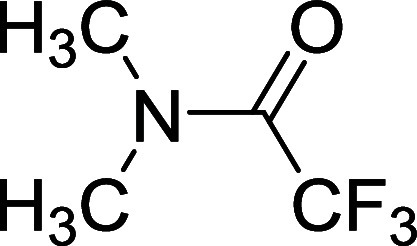



Colorless oil

^1^H NMR (400 MHz, CDCl_3_) δ 3.14 (q, ^5^ *J*_HF_ = 1.6 Hz, 3H), 3.05 (q, ^5^ *J*_HF_ = 0.8 Hz, 3H).

^1^H NMR (400 MHz, C_6_D_6_) δ 2.24 (q, ^5^ *J*_HF_ = 0.8 Hz, 3H), 2.12 (q, ^5^ *J*_HF_ = 1.6 Hz, 3H).

### *N*,*N*-Diethyltrifluoroacetamide (**2**)^11a,13^

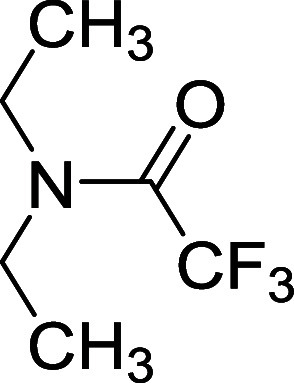



Colorless oil

^1^H NMR (400 MHz, CDCl_3_) δ 3.44 (q, *J* = 7.2 Hz, 2H), 3.44 (qq, *J* = 7.2, ^5^ *J*_HF_ = 1.2 Hz, 2H) 1.24 (t, *J* = 7.2 Hz, 3H), 1.20 (t, *J* = 7.2 Hz, 3H).

^1^H NMR (400 MHz, C_6_D_6_) δ 2.89 (q, *J* = 7.2 Hz, 2H), 2.76 (qq, *J* = 7.2, ^5^ *J*_HF_ = 1.2 Hz, 2H), 0.76 (t, *J* = 7.2 Hz, 3H), 0.59 (t, *J* = 7.2 Hz, 3H).

### *N*-Benzyl-*N*-methyltrifluoroacetamide (**3**)^14,21^

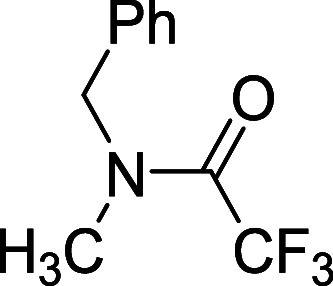



Colorless oil

^1^H NMR (400 MHz, CDCl_3_) *E*-isomer: δ 7.41–7.30 (m, 3H), 7.22–7.20 (m, 2H), 4.63 (s, 2H), 2.92 (q, ^5^ *J*_HF_ = 0.8 Hz, 3H); *Z*-isomer: δ 7.41–7.30 (m, 3H), 7.27–7.23 (m, 2H), 4.64 (s, 2H), 3.05 (q, ^5^ *J*_HF_ = 1.6 Hz, 3H).

^1^H NMR (400 MHz, C_6_D_6_) *E*-isomer: δ 7.04–6.98 (m, 3H), 6.73–6.69 (m, 2H), 3.90 (s, 2H), 2.35 (q, ^5^ *J*_HF_ = 0.4 Hz, 3H); *Z*-isomer: δ 7.04–6.98 (m, 3H), 6.95–6.91 (m, 2H), 4.09 (s, 2H), 2.26 (q, ^5^ *J*_HF_ = 1.6 Hz, 3H).

### *N*-Methyl-*N*-propyltrifluoroacetamide (**4**)^15^

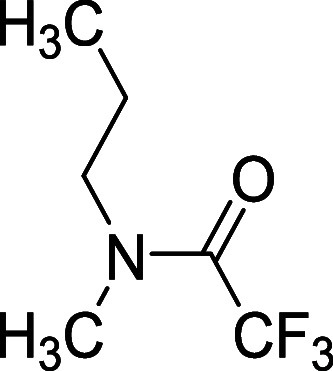



Colorless oil

^1^H NMR (400 MHz, CDCl_3_) *E*-isomer: δ 3.36 (tq, *J* = 7.6 Hz, ^5^ *J*_HF_ = 1.2 Hz, 2H), 3.02 (q, ^5^ *J*_HF_ = 0.4 Hz, 3H), 1.63 (sext, *J* = 7.6 Hz, 2H), 0.93 (t, *J* = 7.6 Hz, 3H); *Z*-isomer: δ 3.41 (t, *J* = 7.6 Hz, 2H), 3.12 (q, ^5^ *J*_HF_ = 1.6 Hz, 3H), 1.66 (sext, *J* = 7.6 Hz, 2H), 0.93 (t, *J* = 7.6 Hz, 3H).

^1^H NMR (400 MHz, C_6_D_6_) *E*-isomer: δ 2.66 (tq, *J* = 7.6 Hz, ^5^ *J*_HF_ = 1.2 Hz, 2H), 2.37 (q, ^5^ *J*_HF_ = 0.4 Hz, 3H), 0.96 (sext, *J* = 7.6 Hz, 2H), 0.41 (t, *J* = 7.6 Hz, 3H); *Z*-isomer: δ 2.85 (t, *J* = 7.6 Hz, 2H), 2.26 (q, ^5^ *J*_HF_ = 1.6 Hz, 3H), 1.09 (sext, *J* = 7.6 Hz, 2H), 0.55 (t, *J* = 7.6 Hz, 3H).

^1^H NMR (400 MHz, C_6_F_6_) *E*-isomer: δ 3.58 (tq, *J* = 7.6 Hz, ^5^ *J*_HF_ = 1.2 Hz, 2H), 3.13 (q, ^5^ *J*_HF_ = 0.4 Hz, 3H), 1.93 (sext, *J* = 7.6, 2H), 1.17 (t, *J* = 7.6 Hz, 3H); *Z*-isomer: δ 3.51 (t, *J* = 7.6 Hz, 2H), 3.35 (q, ^5^ *J*_HF_ = 1.6 Hz, 3H), 1.80 (sext, *J* = 7.6 Hz, 2H), 1.09 (t, *J* = 7.6 Hz, 3H).

### *N*-Benzyl-*N*-ethyltrifluoroacetamide (**5**)^16^

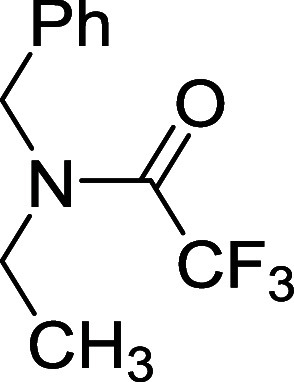



Colorless oil

^1^H NMR (400 MHz, CDCl_3_) *E*-isomer: δ 7.40–7.28 (m, 3H), 7.26–7.21 (m, 2H), 4.62 (s, 2H), 3.37 (q, *J* = 7.2 Hz, 2H), 1.11 (t, *J* = 7.2 Hz, 3H); *Z*-isomer: δ 7.40–7.28 (m, 3H), 7.26–7.21 (m, 2H), 4.66 (s, 2H), 3.41 (qq, *J* = 7.2 Hz, ^5^ *J*_HF_ = 1.2 Hz, 2H), 1.22 (t, *J* = 7.2 Hz, 3H).

^1^H NMR (400 MHz, C_6_D_6_) *E*-isomer: δ 7.06–6.98 (m, 3H), 6.79–6.77 (m, 2H), 4.03 (s, 2H), 2.97 (q, *J* = 7.2 Hz, 2H), 0.69 (t, *J* = 7.2 Hz, 3H); *Z*-isomer: δ 7.06–6.98 (m, 5H), 4.24 (s, 2H), 2.85 (qq, *J* = 7.2 Hz, ^5^ *J*_HF_ = 1.2 Hz, 2H), 0.59 (t, *J* = 7.2 Hz, 3H).

^1^H NMR (400 MHz, C_6_F_6_) *E*-isomer: δ 7.40–7.20 (m, 5H), 4.77 (s, 2H), 3.44 (q, *J* = 7.2 Hz, 2H), 1.24 (t, *J* = 7.2 Hz, 3H); *Z*-isomer: δ 7.40–7.20 (m, 5H), 4.69 (s, 2H), 3.61 (qq, *J* = 7.2 Hz, ^5^ *J*_HF_ = 1.2 Hz, 2H), 1.49 (t, *J* = 7.2 Hz, 3H).

### *N*-Benzyl-*N*-propyltrifluoroacetamide (**6**)^17^

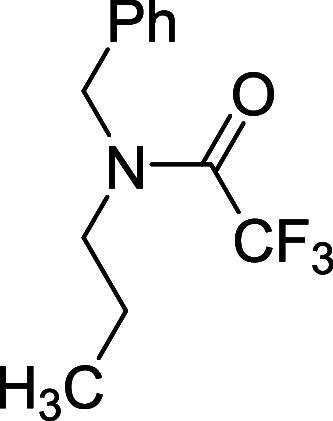



Colorless oil

^1^H NMR (400 MHz, CDCl_3_) *E*-isomer: δ 7.40–7.28 (m, 3H), 7.26–7.19 (m, 2H), 4.62 (s, 2H), 3.27 (t, *J* = 7.2 Hz, 2H), 1.56 (sext, *J* = 7.2 Hz, 2H), 0.86 (t, *J* = 7.2 Hz, 3H); *Z*-isomer: δ 7.40–7.28 (m, 3H), 7.26–7.19 (m, 2H), 4.66 (s, 2H), 3.27 (t, *J* = 7.2 Hz, 2H), 1.65 (sext, *J* = 7.2 Hz, 2H), 0.89 (t, *J* = 7.2 Hz, 3H).

^1^H NMR (400 MHz, C_6_D_6_) *E*-isomer: δ 7.07–6.99 (m, 3H), 6.82–6.79 (m, 2H), 4.10 (s, 2H), 2.99 (t, *J* = 7.6 Hz, 2H), 1.21 (sext, *J* = 7.6 Hz, 2H), 0.52 (t, *J* = 7.6 Hz, 3H); *Z*-isomer: δ 7.07–6.99 (m, 5H), 4.29 (s, 2H), 2.83 (tq, *J* = 7.6 Hz, ^5^ *J*_HF_ = 0.8 Hz, 2H), 1.10 (sext, *J* = 7.6 Hz, 2H), 0.41 (t, *J* = 7.6 Hz, 3H).

^1^H NMR (400 MHz, C_6_F_6_) *E*-isomer: δ 7.39–7.19 (m, 5H), 4.75 (s, 2H), 3.33 (t, *J* = 7.6 Hz, 2H), 1.69 (sext, *J* = 7.6 Hz, 2H), 1.02 (t, *J* = 7.6 Hz, 3H); *Z*-isomer: δ 7.39–7.19 (m, 5H), 4.68 (s, 2H), 3.45 (t, *J* = 7.6 Hz, 2H), 1.91 (sext, *J* = 7.6 Hz, 2H), 1.29 (t, *J* = 7.6 Hz, 3H).

### *N*-Ethyl-*N*-propyltrifluoroacetamide (**7**)^17^

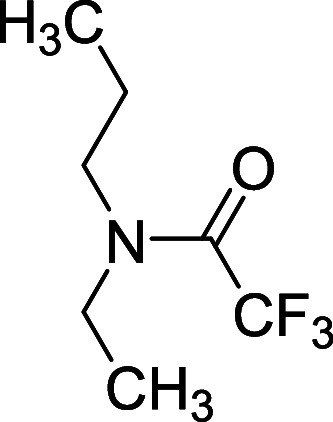



Colorless oil

^1^H NMR (400 MHz, CDCl_3_) *E*-isomer: δ 3.45 (q, *J* = 7.2 Hz, 2H), 3.32 (tq, *J* = 7.2 Hz, ^5^ *J*_HF_ = 1.2 Hz, 2H), 1.64 (sext, *J* = 7.2 Hz, 2H), 1.19 (t, *J* = 7.2 Hz, 3H), 0.93 (t, *J* = 7.2 Hz, 3H); *Z*-isomer: δ 3.45 (q, *J* = 7.2 Hz, 2H), 3.34 (t, *J* = 7.2 Hz, 2H), 1.64 (sext, *J* = 7.2 Hz, 2H), 1.23 (t, *J* = 7.2 Hz, 3H), 0.93 (t, *J* = 7.2 Hz, 3H).

^1^H NMR (400 MHz, C_6_D_6_) *E*-isomer: δ 2.91 (t, *J* = 7.6 Hz, 2H), 2.72 (tq, *J* = 7.2 Hz, ^5^ *J*_HF_ = 1.2 Hz, 2H), 1.24 (sext, *J* = 7.2 Hz, 2H), 0.78 (t, *J* = 7.6 Hz, 3H), 0.45 (t, *J* = 7.6 Hz, 3H); *Z*-isomer: δ 2.88 (q, *J* = 7.2 Hz, 2H), 2.78 (qq, *J* = 7.2 Hz, ^5^ *J*_HF_ = 1.2 Hz, 2H), 1.04 (sext, *J* = 7.6 Hz, 2H), 0.59 (t, *J* = 7.2 Hz, 3H), 0.58 (t, *J* = 7.2 Hz, 3H).

^1^H NMR (400 MHz, C_6_F_6_) *E*-isomer: δ 3.56 (q, *J* = 7.2 Hz, 2H), 3.54 (tq, *J* = 7.2 Hz, ^5^ *J*_HF_ = 1.2 Hz, 2H), 1.91 (sext, *J* = 7.2, 2H), 1.32 (t, *J* = 7.2 Hz, 3H), 1.16 (t, *J* = 7.2 Hz, 3H); *Z*-isomer: δ 3.68 (qq, *J* = 7.2 Hz, ^5^ *J*_HF_ = 0.4 Hz, 2H), 3.45 (t, *J* = 7.2 Hz, 2H), 1.76 (sext, *J* = 7.2 Hz, 2H), 1.47 (t, *J* = 7.2 Hz, 3H), 1.08 (t, *J* = 7.2 Hz, 3H).

### *N*-Allyl-*N*-methyltrifluoroacetamide (**8**)^19^

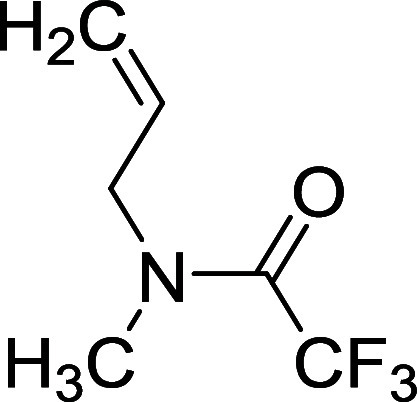



Colorless oil

^1^H NMR (400 MHz, CDCl_3_) *E*-isomer: δ 5.81–5.71 (m, 1H), 5.33–5.20 (m, 2H), 4.01 (dq, *J* = 6.0 Hz, ^5^ *J*_HF_ = 1.2 Hz, 2H), 3.00 (q, ^5^ *J*_HF_ = 0.8 Hz, 3H); *Z*-isomer: δ 5.81–5.71 (m, 1H), 5.33–5.20 (m, 2H), 4.05 (d, *J* = 6.0 Hz, 2H), 3.09 (q, ^5^ *J*_HF_ = 1.6 Hz, 3H).

^1^H NMR (400 MHz, C_6_D_6_) *E*-isomer: δ 5.19–5.08 (m, 1H), 4.85–4.61 (m, 2H), 3.24 (dq, *J* = 6.0 Hz, ^5^ *J*_HF_ = 1.2 Hz, 2H), 2.39 (q, ^5^ *J*_HF_ = 0.8 Hz, 3H); *Z*-isomer: δ 5.34–5.23 (m, 1H), 4.85–4.61 (m, 2H), 3.46 (d, *J* = 6.0 Hz, 2H), 2.29 (q, ^5^ *J*_HF_ = 1.6 Hz, 3H).

^1^H NMR (400 MHz, C_6_F_6_) *E*-isomer: δ 6.04–5.86 (m, 1H), 5.48–5.43 (m, 2H), 4.20 (dq, *J* = 6.0 Hz, ^5^ *J*_HF_ = 1.2 Hz, 2H), 3.10 (q, ^5^ *J*_HF_ = 0.8 Hz, 3H); *Z*-isomer: δ 6.04–5.86 (m, 1H), 5.40–5.34 (m, 2H), 4.12 (d, *J* = 6.0 Hz, 2H), 3.32 (q, ^5^ *J*_HF_ = 1.6 Hz, 3H).

## Supplementary Material



## Data Availability

The data underlying this study are available in the published article and Supporting Information.

## Notes

^1^H NMR spectrum of **1** in C_6_D_6_, ^13^C­{^1^H} NMR spectrum of **1** in C_6_D_6_, ^1^H NMR spectra of **2** in CDCl_3_, ^13^C­{^1^H} NMR spectrum of **2** in CDCl_3_, ^1^H NMR spectrum of **2** in C_6_D_6_, ^13^C­{^1^H} NMR spectrum of **2** in C_6_D_6_, HSQC spectrum of **2** in C_6_D_6_, ^1^H NMR spectra of **3** in CDCl_3_, ^13^C­{^1^H} NMR spectrum of **3** in CDCl_3_, HSQC spectrum of **3** in CDCl_3_, ^1^H NMR spectrum of **3** in C_6_D_6_, ^13^C­{^1^H} NMR spectrum of **3** in C_6_D_6_, ^1^H NMR spectrum of **4** (C_6_D_6_, CDCl_3_), ^1^H NMR spectrum of **5** (C_6_D_6_, CDCl_3_), ^1^H NMR spectrum of **6** (C_6_D_6_), ^1^H NMR spectrum of **7** (C_6_D_6_), ^1^H NMR spectrum of **8** (C_6_D_6_), ASIS of compounds **1–8** (ASIS (Δδ): Δδ = δCDCl_3_ – δC_6_D_6_), ASIS of compounds **1–8** (ASIS (Δδ): Δδ = δCDCl_3_ – δC_6_F_6_), NMR spectra of **4**, NMR spectra of **5**, NMR spectra of **6**, NMR spectra of **7**, NMR spectra of **8**, calculation method, conformational search of **1**, conformational search of **5-II**, and conformational search of **6-II** (PDF)
