# Supplementary material for: Stereochemical Analysis of Tertiary Trifluoroacetamides Leveraging Both Through-Space 1H···19F Spin–Spin Couplings and Anisotropic Solvent-Induced Shifts
Source: J Org Chem. 2026 Feb 24;91(9):3501–11. doi: 10.1021/acs.joc.5c02877 (PMC12973303; doi:10.1021/acs.joc.5c02877)
Supplement: Supplementary file 1 [file jo5c02877_si_001.pdf]

Supporting Information

Stereochemical analysis of tertiary trifluoroacetamides leveraging both through-space $^{1}H\cdots{}^{19}F$ spin–spin couplings and anisotropic solvent-induced shifts

Kizuki Watanabe,[1] Ryota Takano,[1] Hidetsugu Tabata,[2] Kiriko Hirano,[3] Motoo Iida,[1] Tetsuta Oshitari,[2] Hideaki Natsugari,[4] Takenori Kusumi,[5] Kayo Nakamura*,[1] and Hideyo Takahashi*[1]

[1]Faculty of Pharmaceutical Sciences, Tokyo University of Science, 6-3-1 Niijuku, Katsushika-ku, Tokyo 125-8585, Japan

[2]Faculty of Pharma Sciences, Teikyo University, 2-11-1 Kaga, Itabashi-ku, Tokyo 173-8605, Japan

[3]Bruker Japan K.K., 3-9 Moriya, Kanagawa-ku, Yokohama, Kanagawa 221-0022, Japan

[4]Faculty of Pharmacy, Niigata University of Pharmacy and Medical and Life Sciences, 265-1 Higashijima, Akiha-ku, Niigata 956-8603, Japan.

[5]Professor Emeritus, The University of Tokushima, 1–78–1 Shomachi, Tokushima 770–8505, Japan.

*Corresponding Authors: hide-tak@rs.tus.ac.jp
kayo_nakamura@rs.tus.ac.jp,

Contents

1. ^{1}H NMR spectrum of 1 in C_6D_6. ....S3
2. $^{13}C\{^{1}H\}$ NMR spectrum of 1 in C_6D_6. ....S4
3. ^{1}H NMR spectra of 2 in $CDCl_3$. S5
4. $^{13}C\{^{1}H\}$ NMR spectrum of 2 in $CDCl_3$. S6
5. ^{1}H NMR spectrum of 2 in C_6D_6. ....S7
6. $^{13}C\{^{1}H\}$ NMR spectrum of 2 in C_6D_6. ....S8
7. HSQC spectrum of 2 in C_6D_6. ....S9
8. ^{1}H NMR spectra of 3 in $CDCl_3$. S10
9. $^{13}C\{^{1}H\}$ NMR spectrum of 3 in $CDCl_3$. S11
10. HSQC spectrum of 3 in $CDCl_3$. S12
11. ^{1}H NMR spectrum of 3 in C_6D_6. ....S13
12. $^{13}C\{^{1}H\}$ NMR spectrum of 3 in C_6D_6. ....S14
13. ^{1}H NMR spectra of 4 (C_6D_6, $CDCl_3$). S15
14. ^{1}H NMR spectra of 5 (C_6D_6, $CDCl_3$). S16
15. ^{1}H NMR spectra of 6 (C_6D_6, $CDCl_3$). S17
16. ^{1}H NMR spectra of 7 (C_6D_6, $CDCl_3$). S18
17. ^{1}H NMR spectra of 8 (C_6D_6, $CDCl_3$). S19
18. ASIS of compounds 1-8 (ASIS ($\Delta\delta$): $\Delta\delta = \delta_{CDCl_3} - \delta_{C_6D_6}$) S20
19. ASIS of compounds 1-8 (ASIS ($\Delta\delta$): $\Delta\delta = \delta_{CDCl_3} - \delta_{C_6F_6}$) S22
20. NMR spectra of 4. ....S24
21. NMR spectra of 5. ....S29
22. NMR spectra of 6. ....S34
23. NMR spectra of 7. ....S39
24. NMR spectra of 8. ....S44
25. Calculation method. S49
26. Conformational search of 1. ....S49
27. Conformational search of 5-II. S50
28. Conformational search of 6-II. S52

1. ^{1}H NMR spectrum of 1 in C_6D_6.

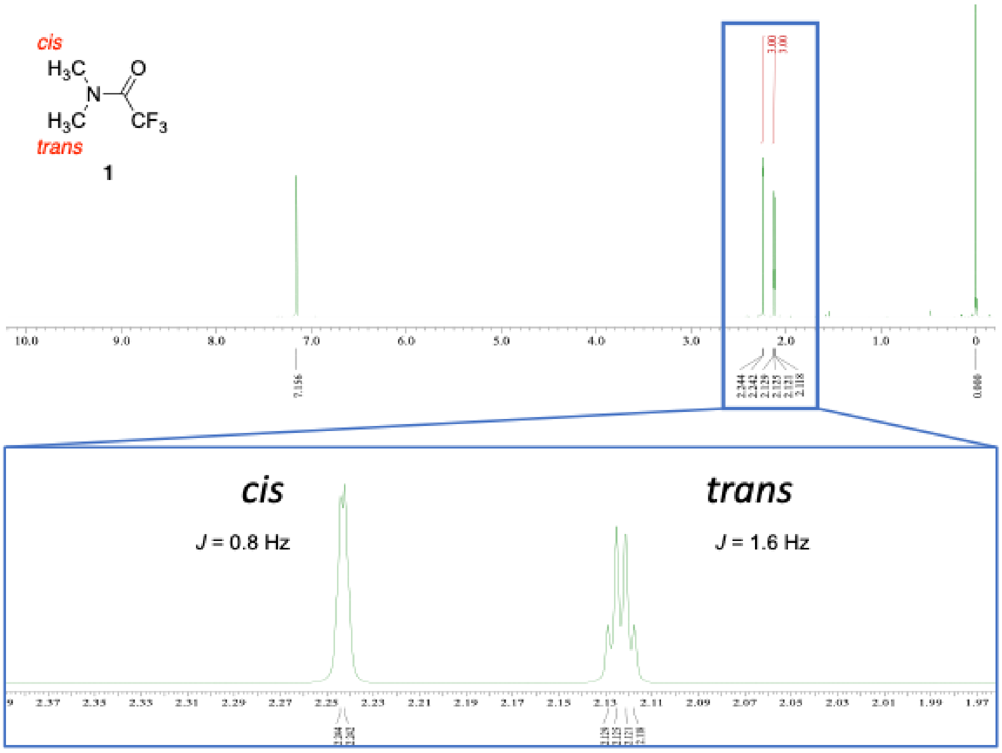

Figure S1. ^{1}H NMR spectrum of **1** (400 MHz, C_6D_6).

2. $^{13}C\{^1H\}$ NMR spectrum of 1 in C_6D_6.

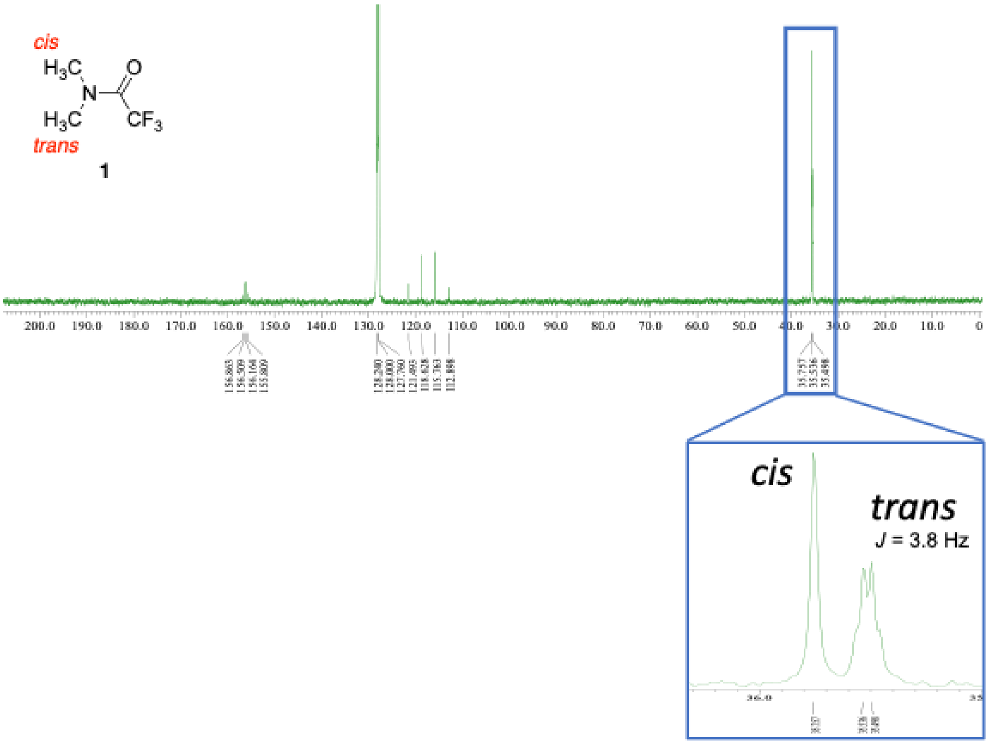

Figure S2. $^{13}C\{^1H\}$ NMR spectrum of **1** (100 MHz, C_6D_6).

3. ^{1}H NMR spectra of 2 in $CDCl_3$.

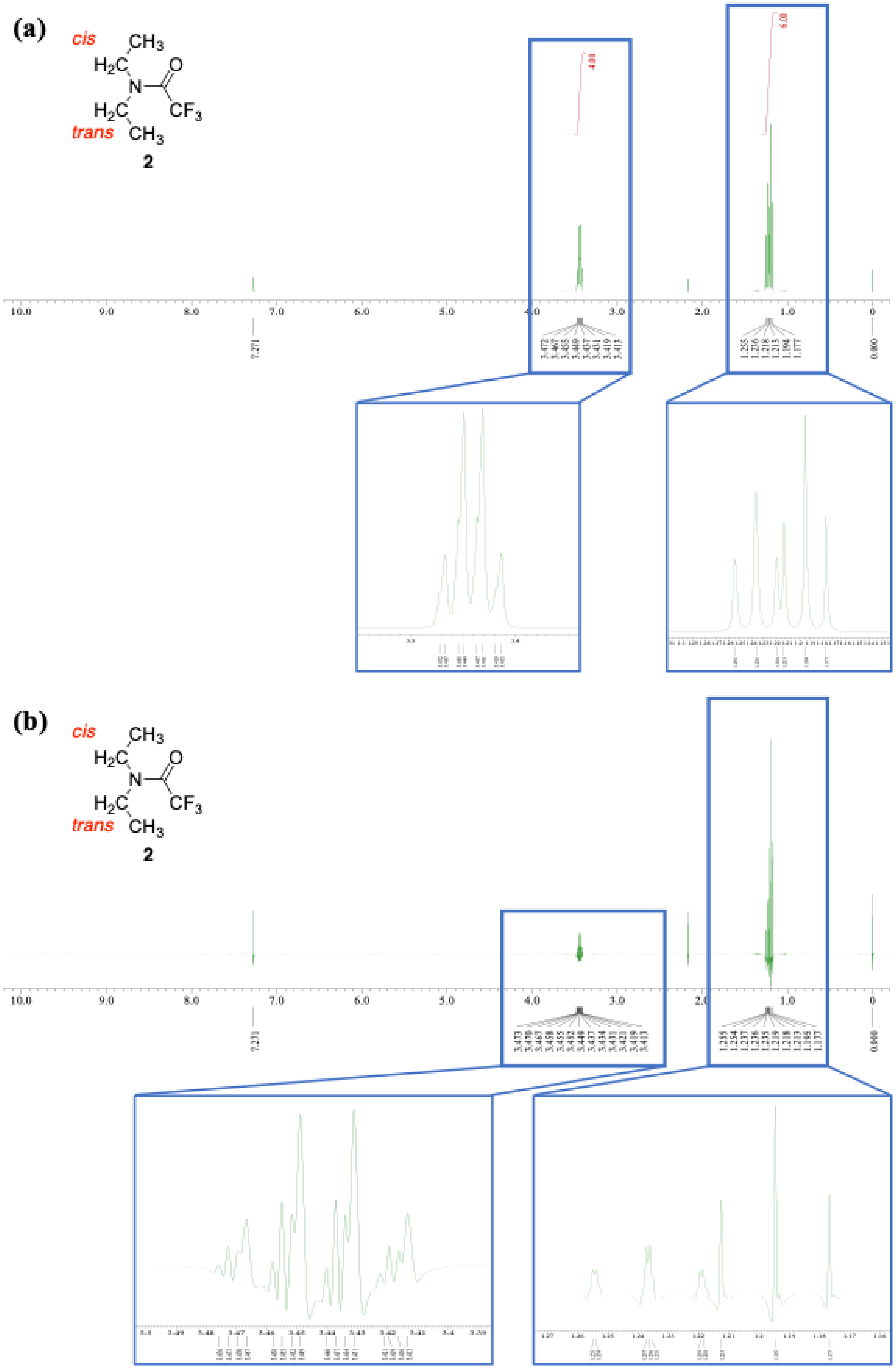

Figure S3. (a) ^{1}H NMR spectrum of **2** (400 MHz, $CDCl_3$) (b) ^{1}H NMR spectrum of **2** (400 MHz, $CDCl_3$) after sine bell window function.

4. $^{13}C\{^{1}H\}$ NMR spectrum of 2 in $CDCl_3$.

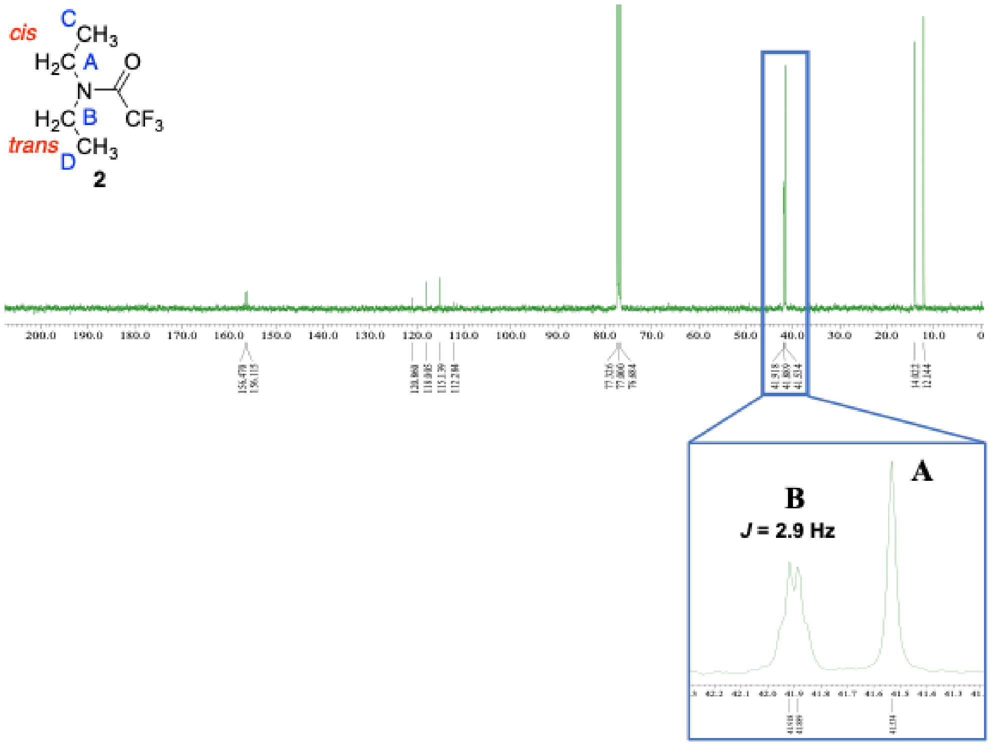

Figure S4. $^{13}C\{^{1}H\}$ NMR spectrum of **2** (100 MHz, $CDCl_3$).

5. ^1H NMR spectrum of 2 in C_6D_6.

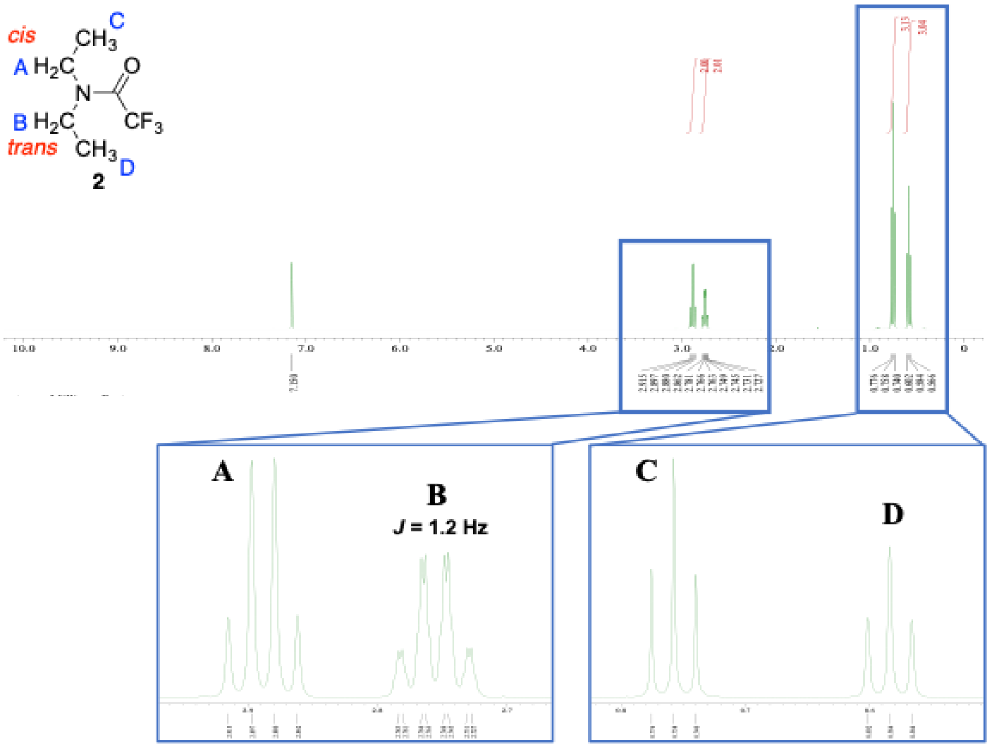

Figure S5. ^1H NMR spectrum of **2** (400 MHz, C_6D_6).

6. $^{13}C\{^1H\}$ NMR spectrum of 2 in C_6D_6.

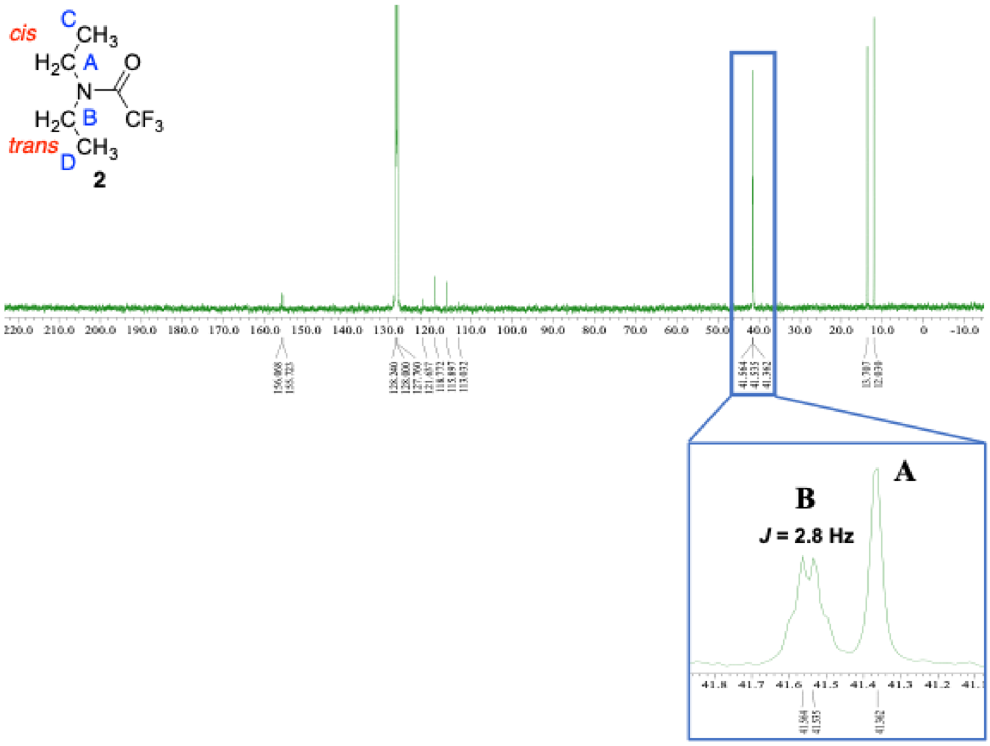

Figure S6. $^{13}C\{^1H\}$ NMR spectrum of **2** (100 MHz, C_6D_6).

7. HSQC spectrum of 2 in C_6D_6.

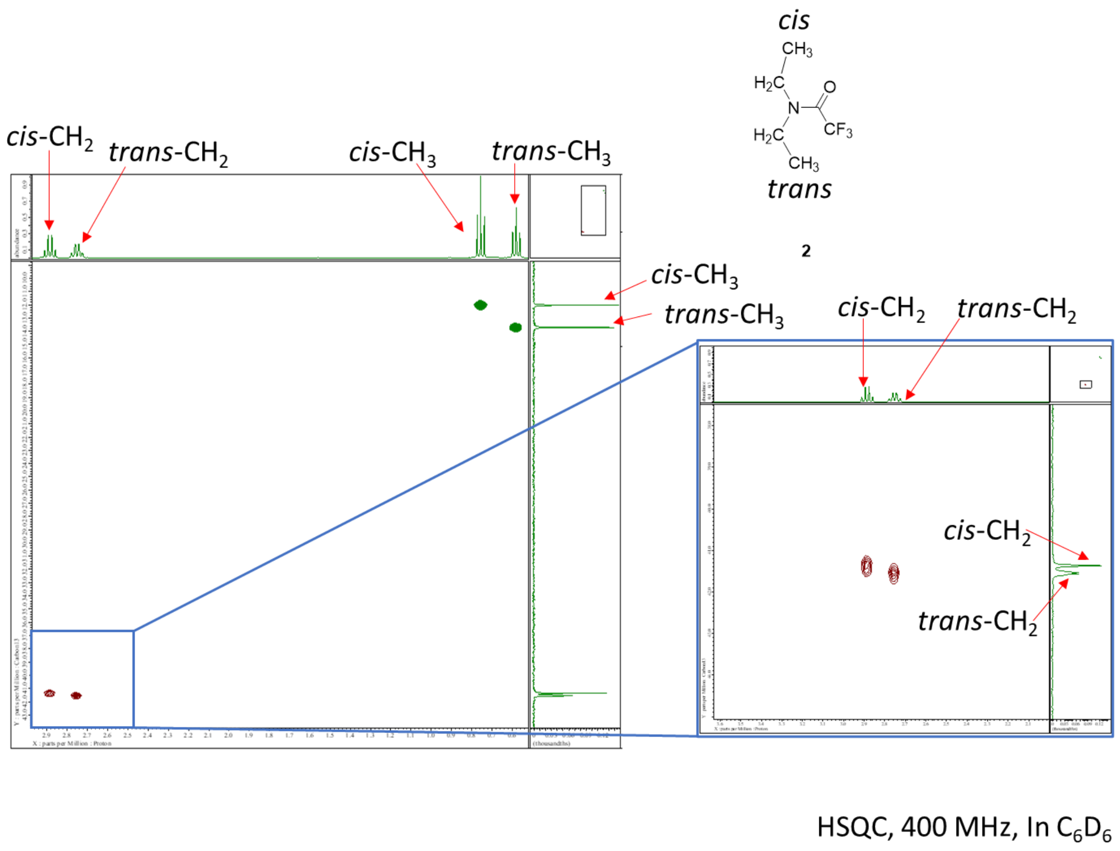

Figure S7. HSQC spectrum of **2** (400 MHz, C_6D_6).

8. ^1H NMR spectra of 3 in $CDCl_3$.

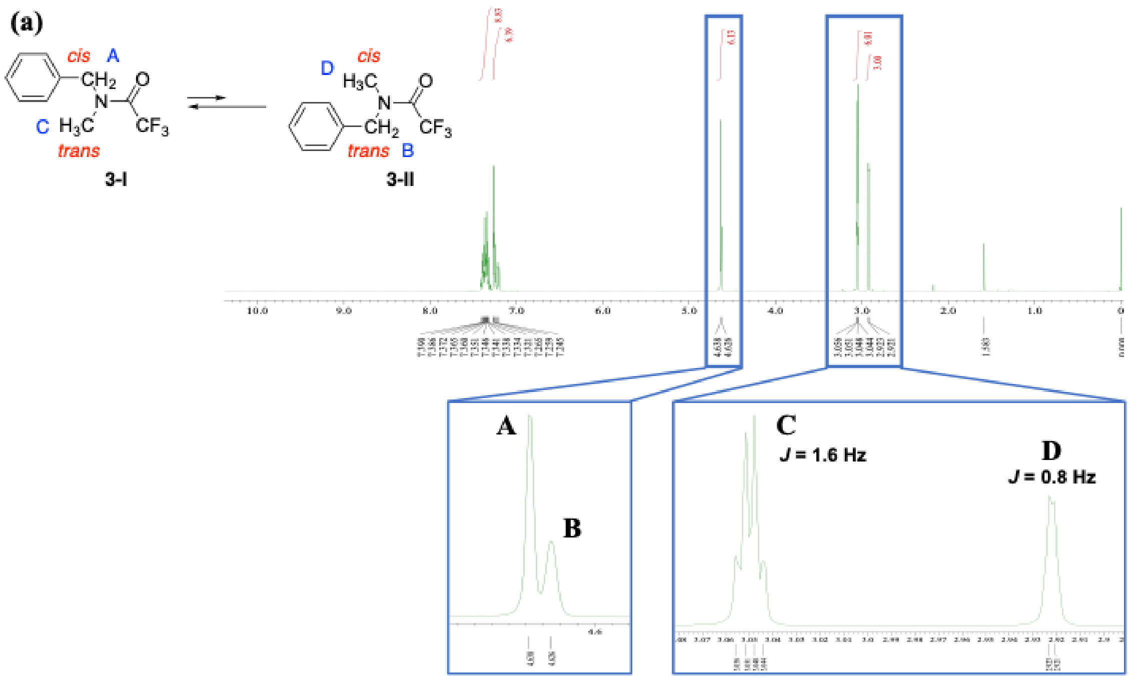

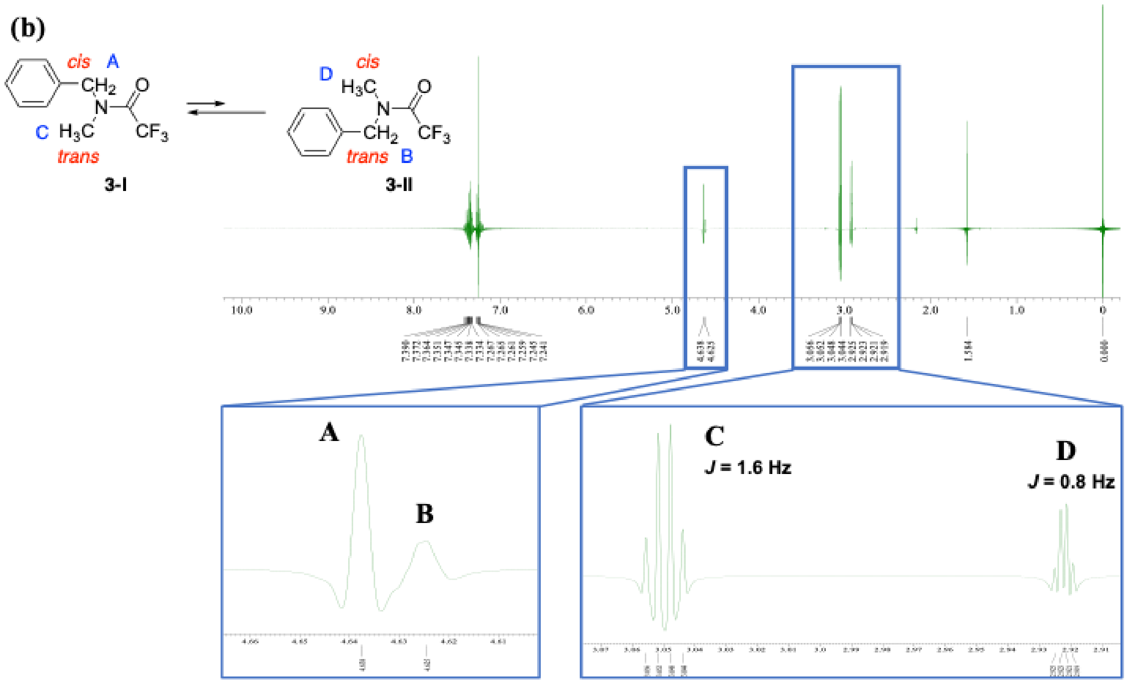

Figure S8. (a) ^1H NMR spectrum of **3** (400 MHz, $CDCl_3$) (b) ^1H NMR spectrum of **3** (400 MHz, $CDCl_3$) after sine bell window function.

9. $^{13}C\{^1H\}$ NMR spectrum of 3 in $CDCl_3$.

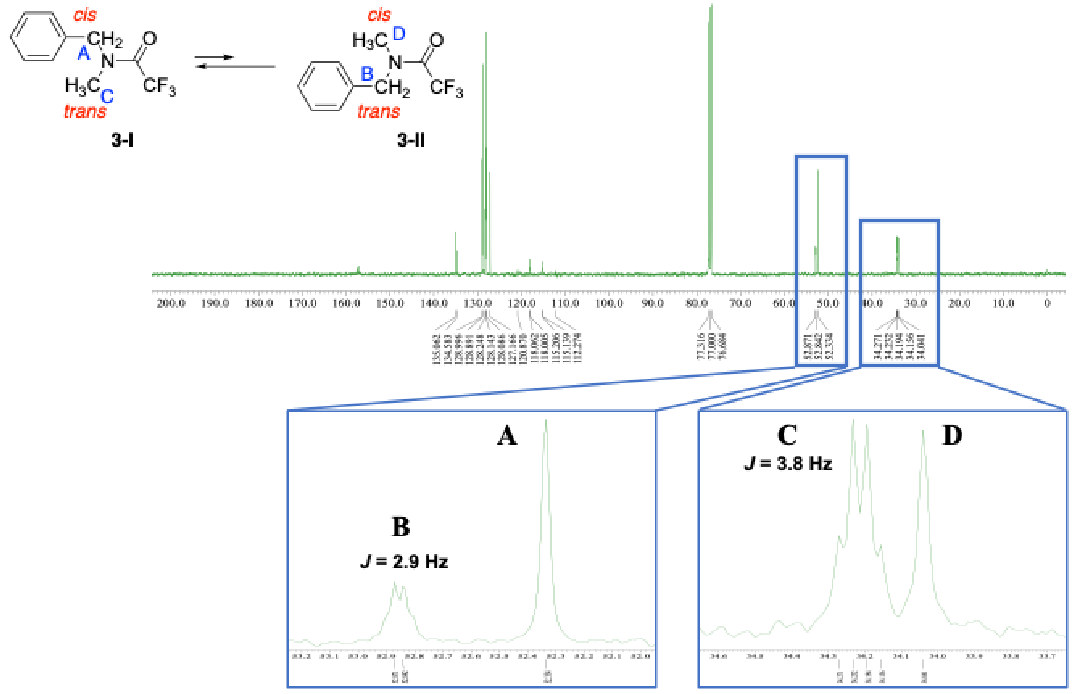

Figure S9. $^{13}C\{^1H\}$ NMR spectrum of **3** (100 MHz, $CDCl_3$).

10. HSQC spectrum of 3 in $CDCl_3$.

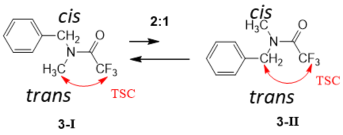

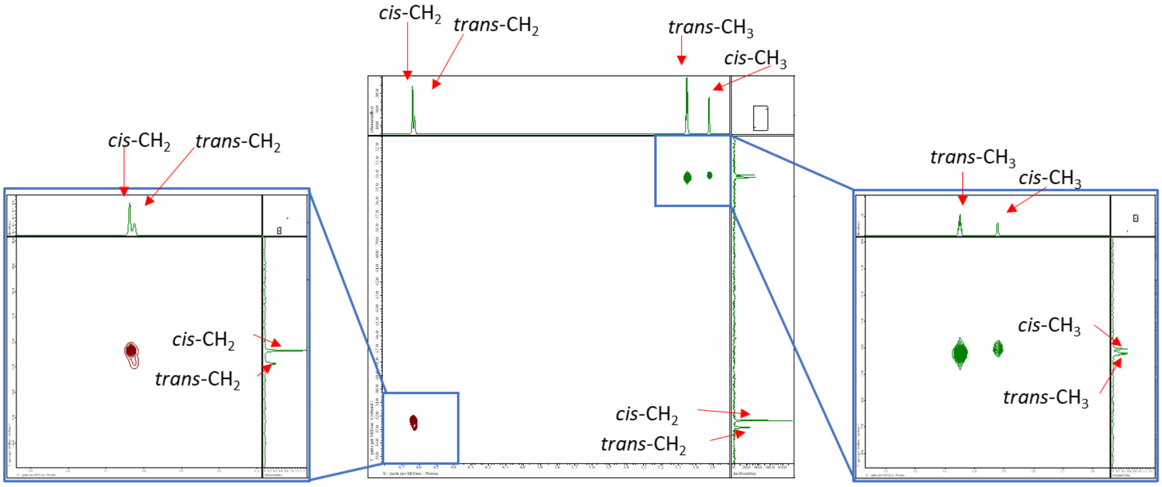

Figure S10. HSQC spectrum of **3** (400 MHz, $CDCl_3$).

11. ^{1}H NMR spectrum of 3 in C_6D_6.

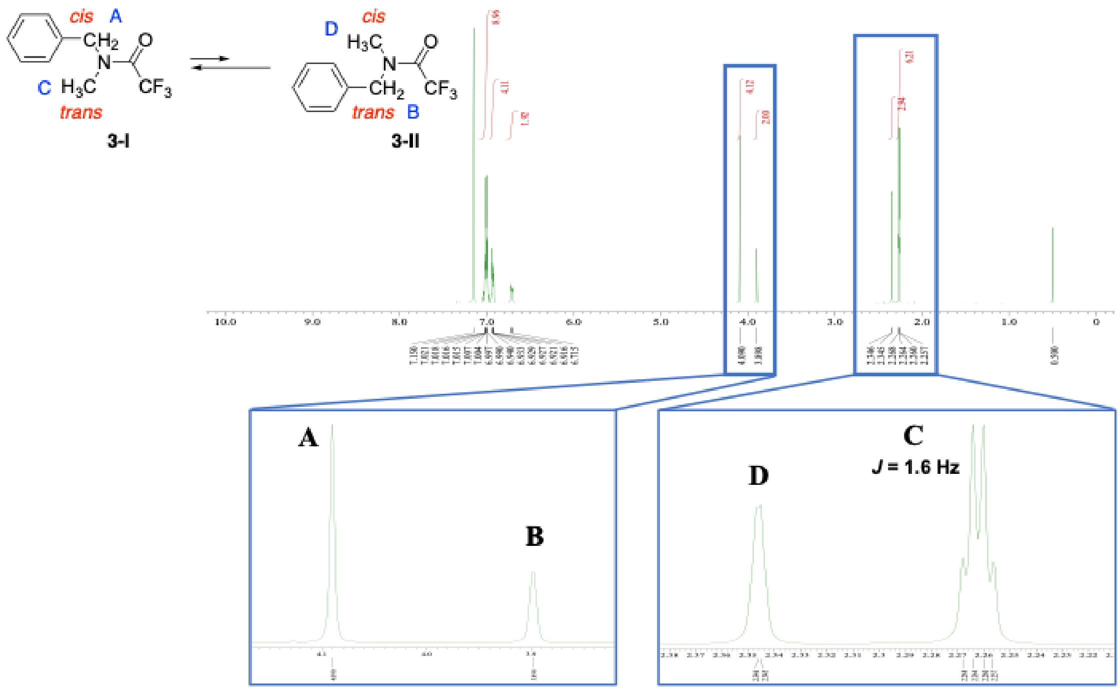

Figure S11. ^{1}H NMR spectrum of **3** (400 MHz, C_6D_6)

12. $^{13}C\{^1H\}$ NMR spectrum of 3 in C_6D_6.

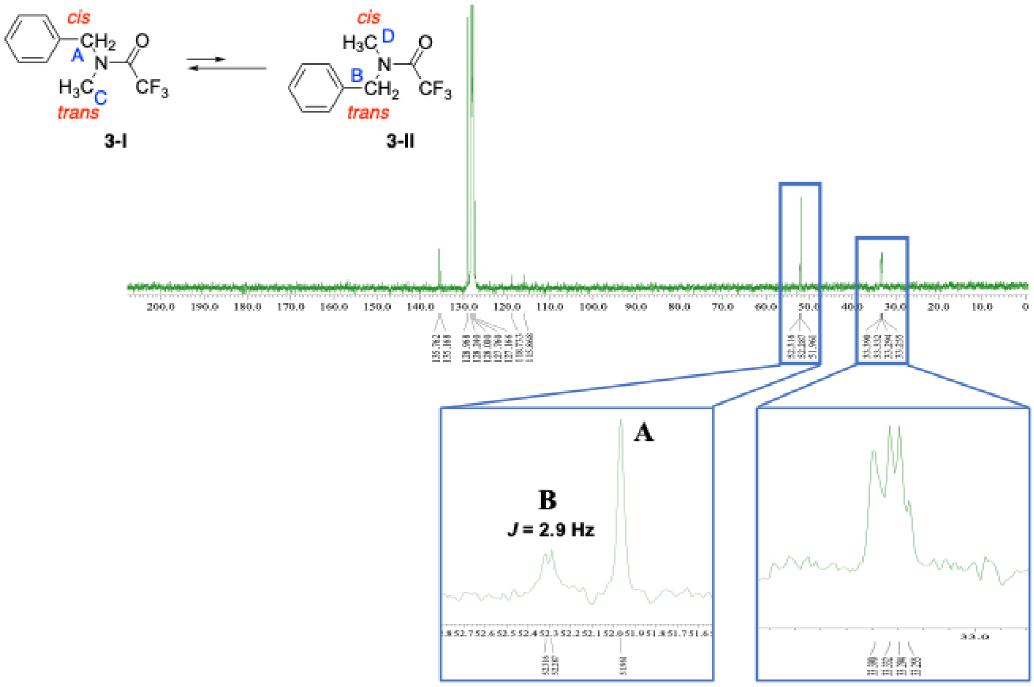

Figure S12. $^{13}C\{^1H\}$ NMR spectrum of **3** (100 MHz, C_6D_6).

13. ^{1}H NMR spectrum of 4 (C_6D_6, $CDCl_3$).

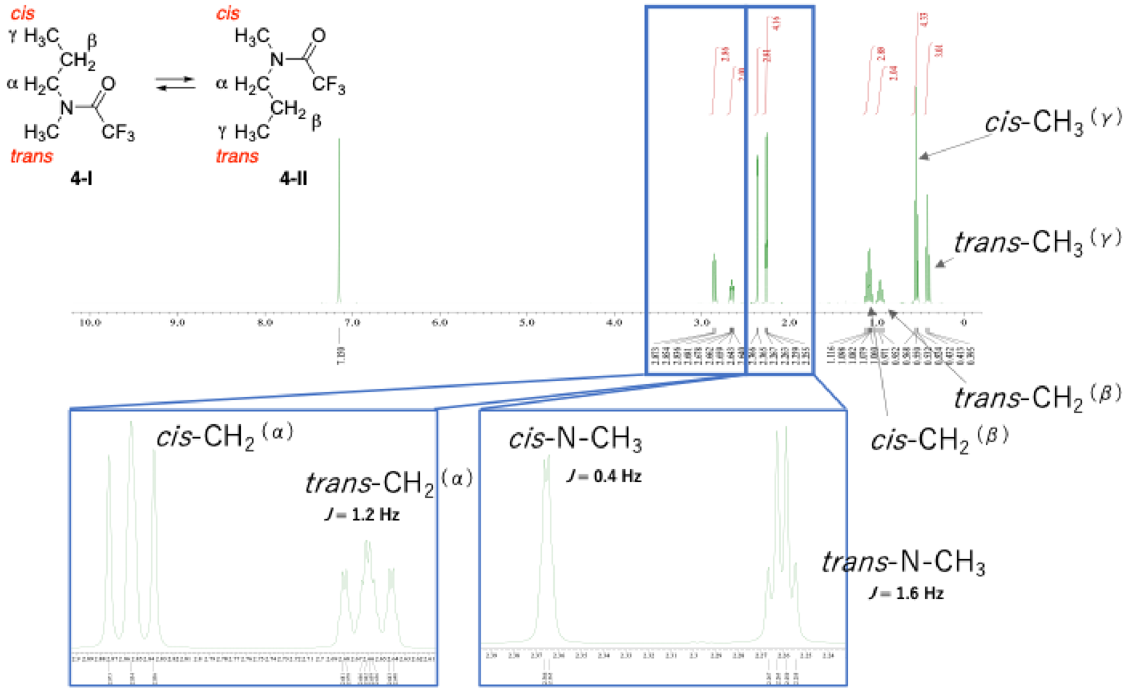

Figure S13. ^{1}H NMR spectra of **4** (400 MHz, C_6D_6).

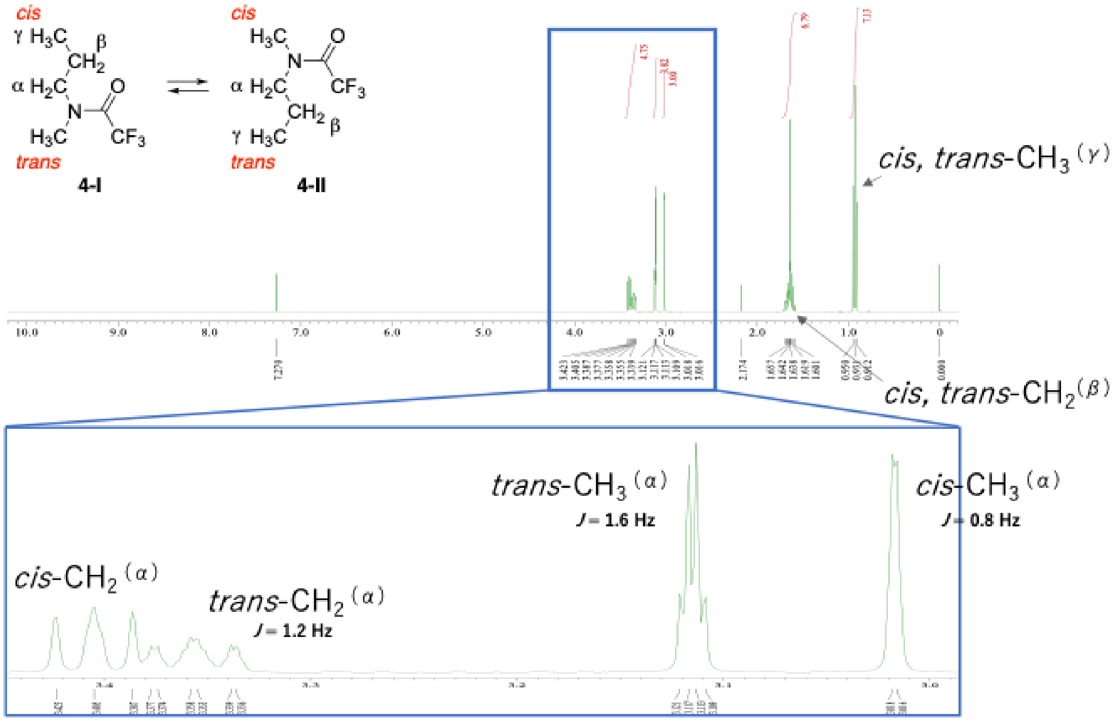

Figure S14. ^{1}H NMR spectra of **4** (400 MHz, $CDCl_3$).

14. ^{1}H NMR spectrum of 5 (C_6D_6, $CDCl_3$).

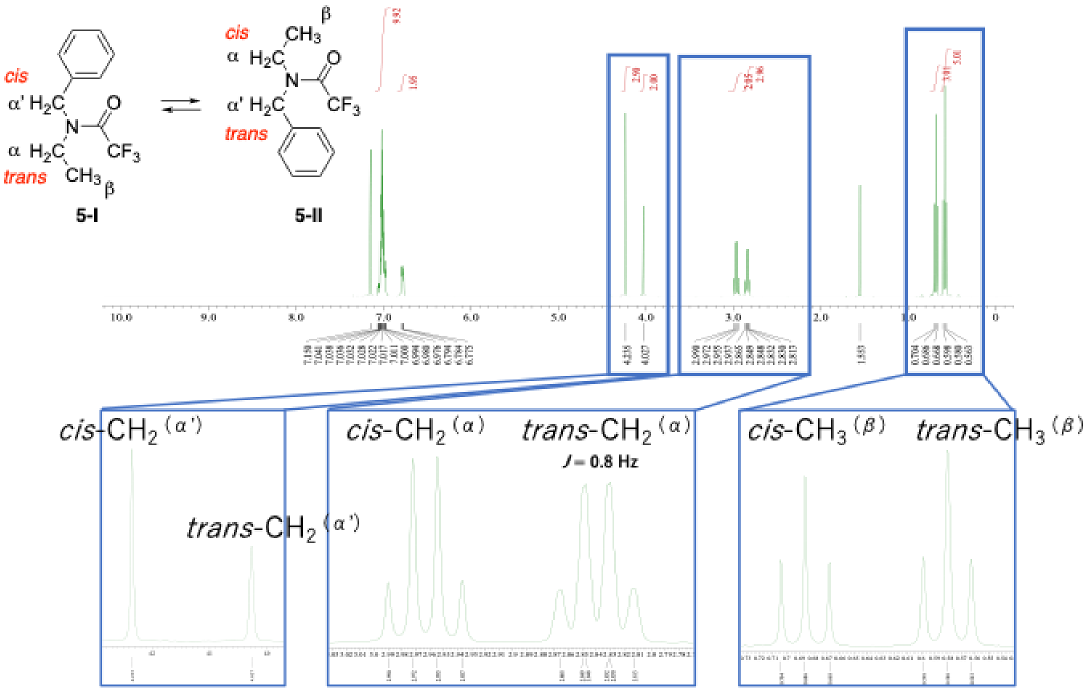

Figure S15. ^{1}H NMR spectra of **5** (400 MHz, C_6D_6).

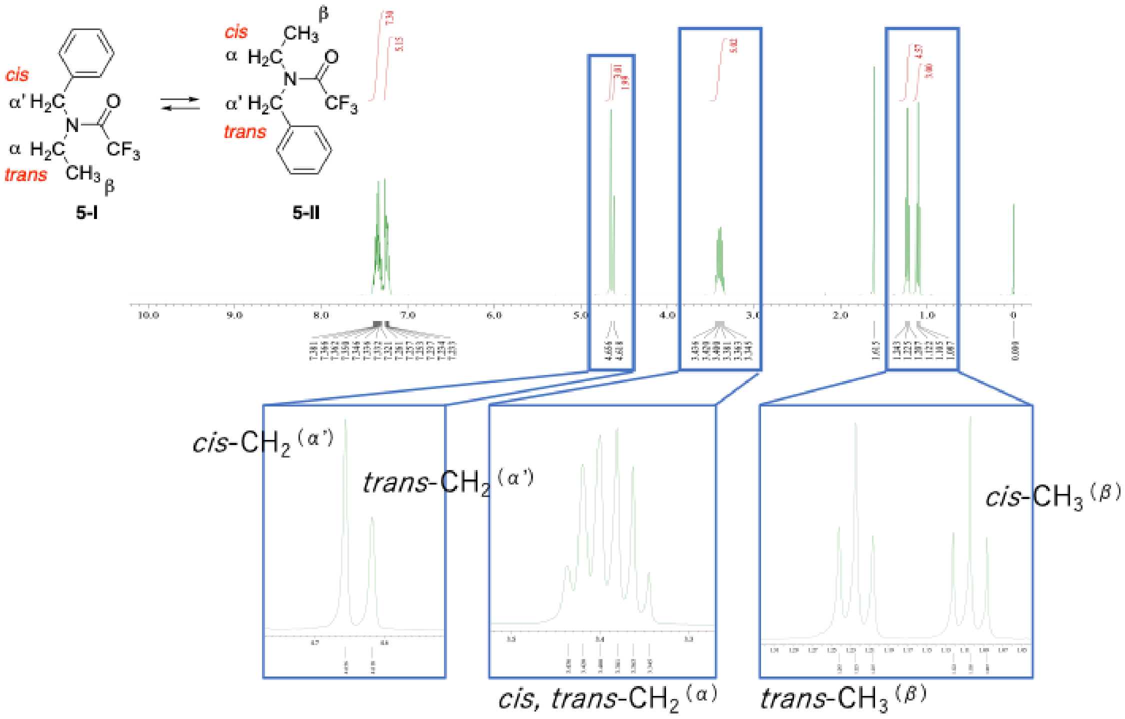

Figure S16. ^{1}H NMR spectra of **5** (400 MHz, $CDCl_3$).

15. ^{1}H NMR spectrum of 6 (C_6D_6, $CDCl_3$).

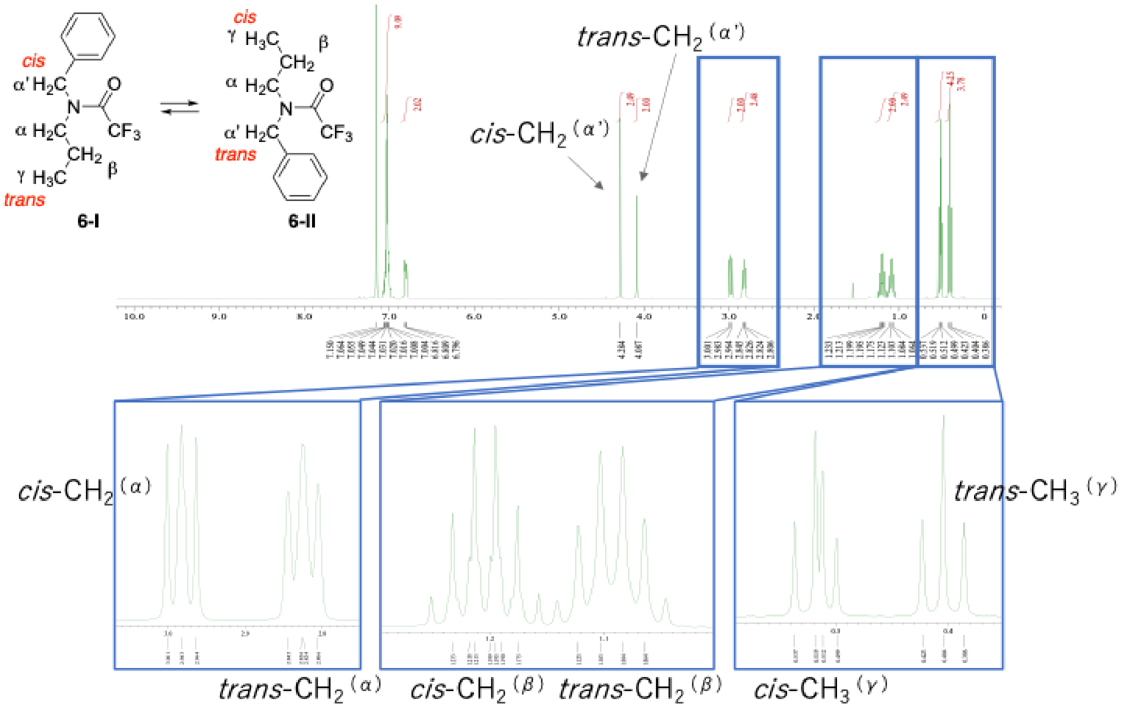

Figure S17. ^{1}H NMR spectrum of **6** (400 MHz, C_6D_6).

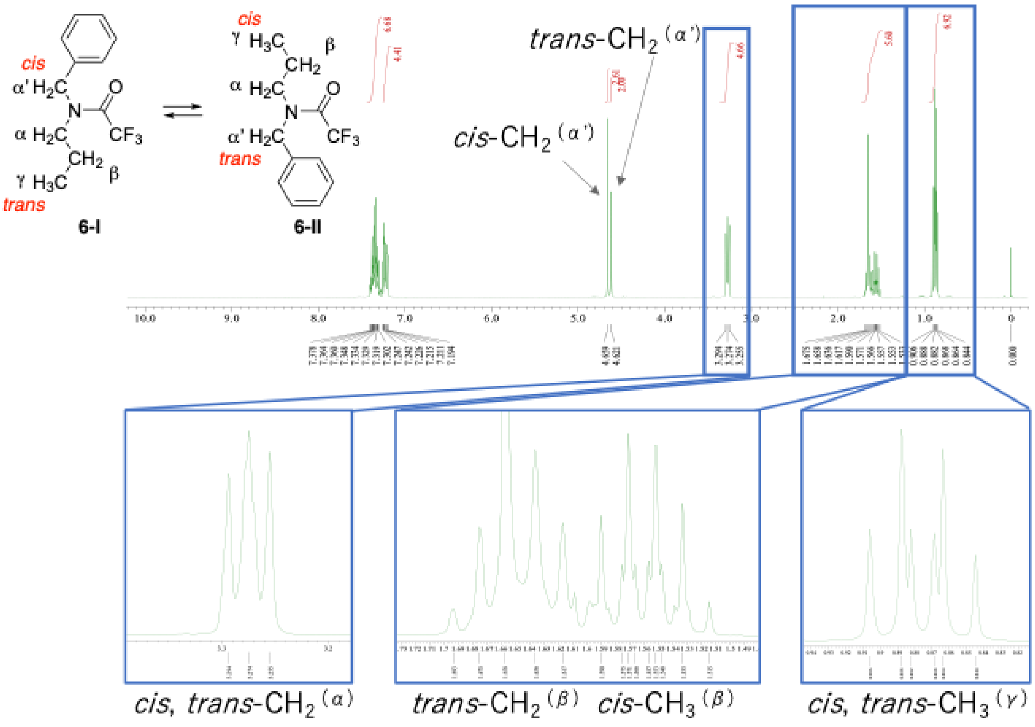

Figure S18. ^{1}H NMR spectrum of **6** (400 MHz, $CDCl_3$).

16. ^{1}H NMR spectrum of **7** (C_6D_6, $CDCl_3$).

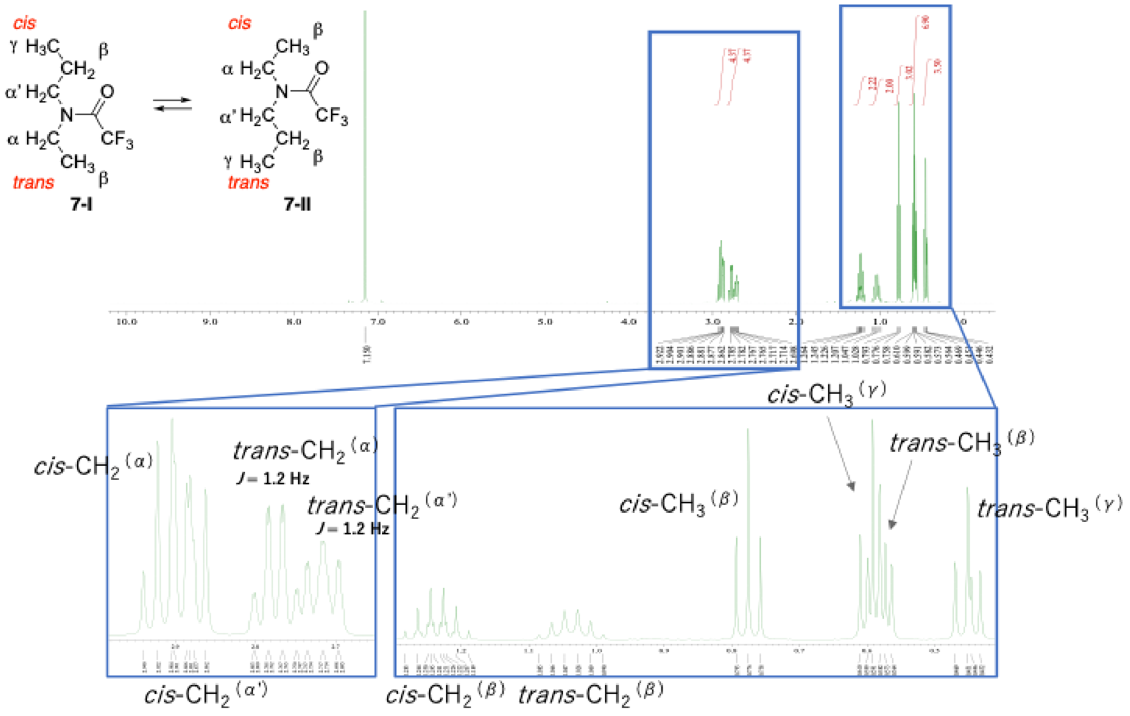

Figure S19. ^{1}H NMR spectrum of **7** (400 MHz, C_6D_6).

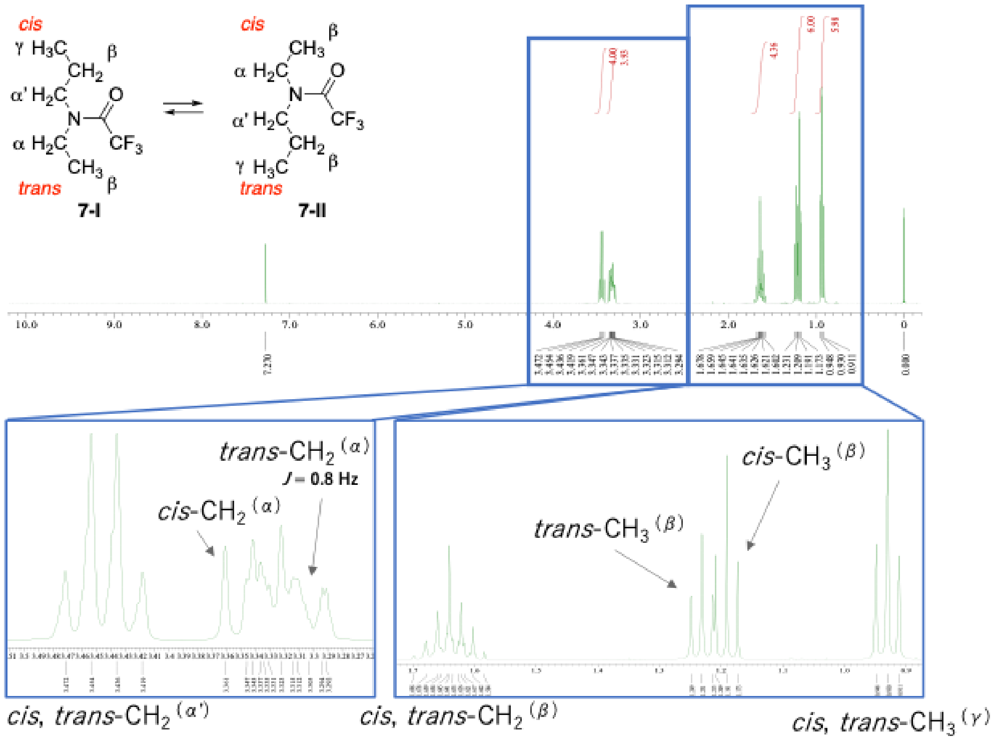

Figure S20. ^{1}H NMR spectrum of **7** (400 MHz, $CDCl_3$).

17. ^{1}H NMR spectrum of 8 (C_6D_6, $CDCl_3$).

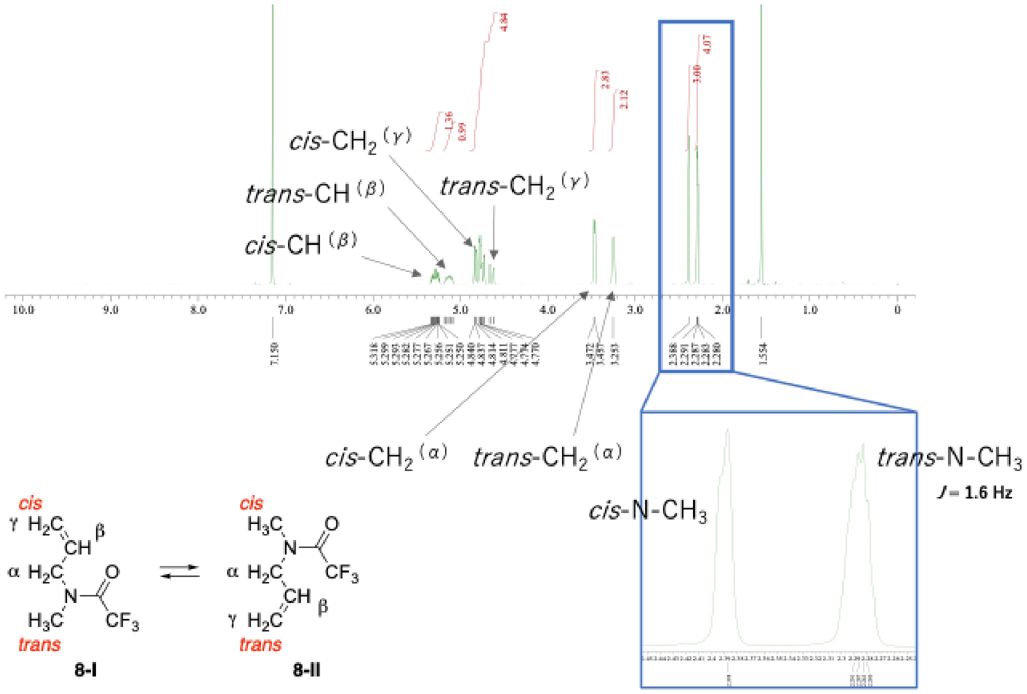

Figure S21. ^{1}H NMR spectrum of **8** (400 MHz, C_6D_6).

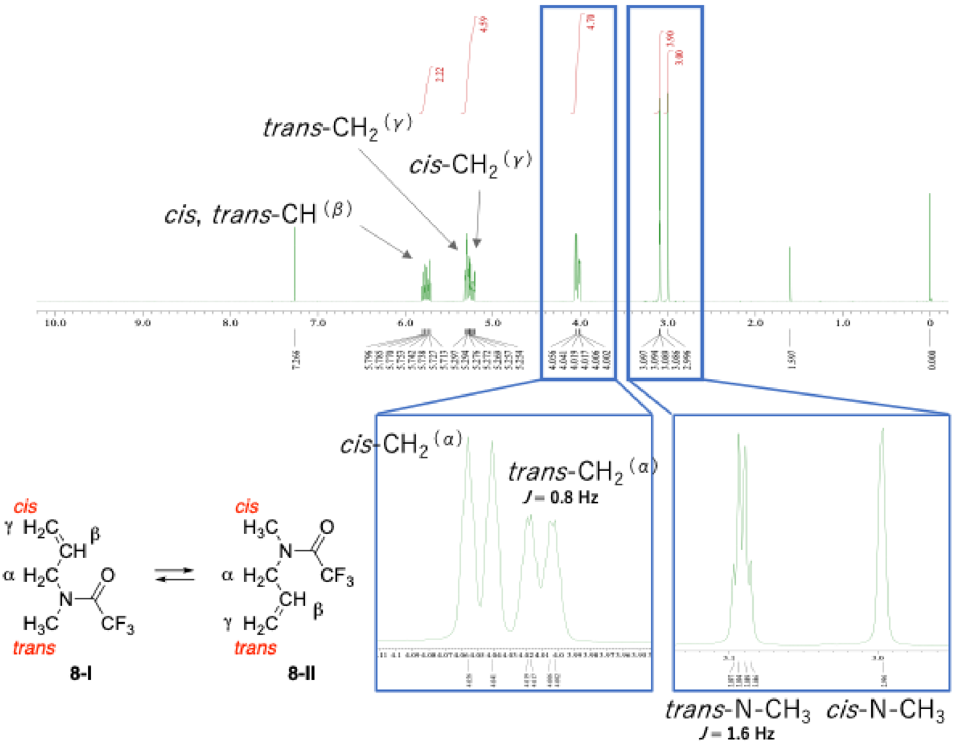

Figure S22. ^{1}H NMR spectrum of **8** (400 MHz, $CDCl_3$).

18. ASIS of compounds 1-8 (ASIS (Δδ): $\Delta\delta = \delta_{CDCl_3} - \delta_{C_6D_6}$).

Table S1. ASIS of compounds **1-8** (ASIS (Δδ): $\Delta\delta = \delta_{CDCl_3} - \delta_{C_6D_6}$).

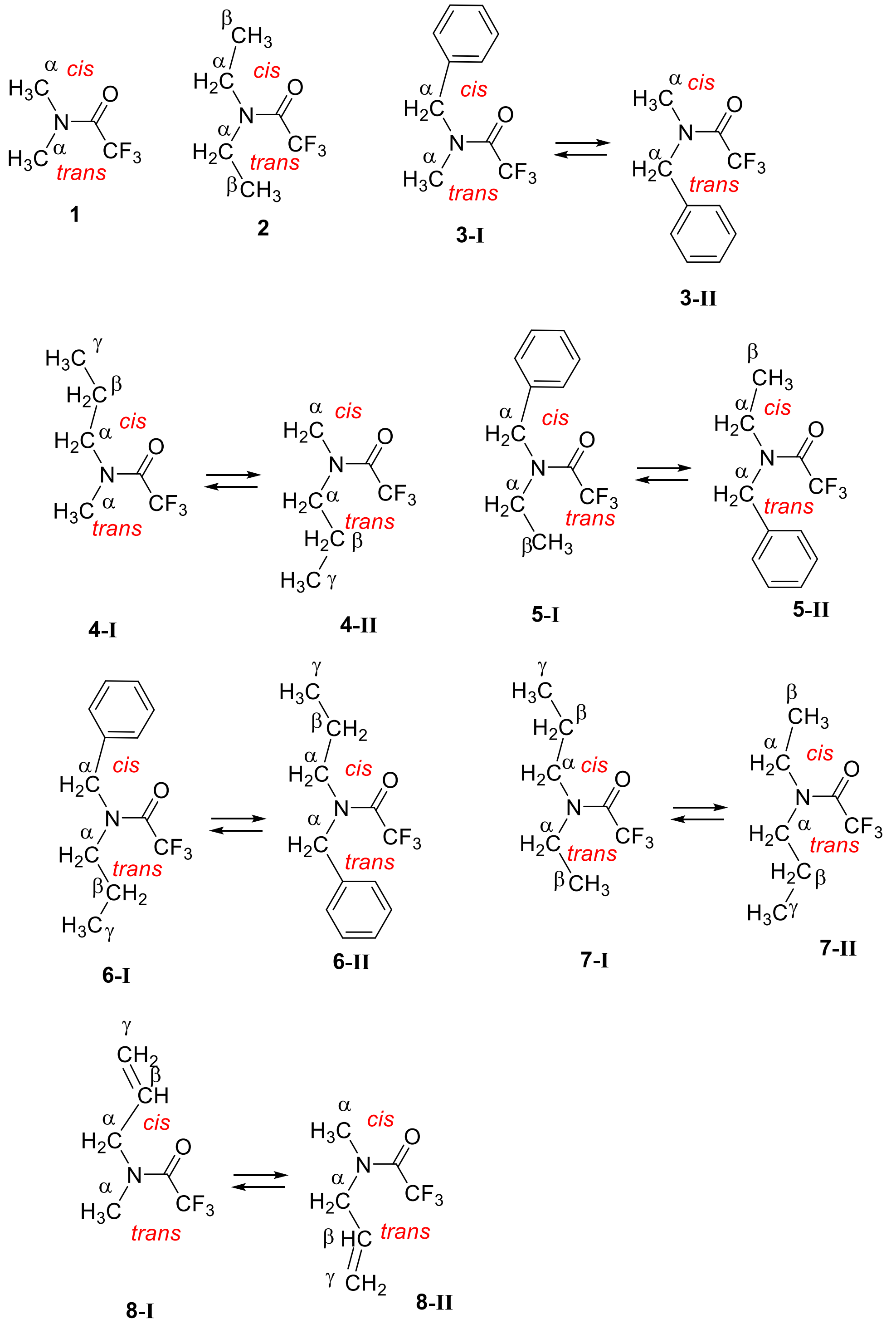

ASIS ($\Delta\delta$): $\Delta\delta = \delta_{CDCl_3} - \delta_{C_6D_6}$							
		cis			*trans*		
Entry	Compounds	C^{α}	C^{β}	C^{γ}	C^{α}	C^{β}	C^{γ}
1	**1**	0.82	—	—	1.03	—	—
2	**2**	0.55	0.44	—	0.68	0.65	—
3	**3-I**	0.55	—	—	0.79	—	—
	**3-II**	0.57	—	—	0.73	—	—
4	**4-I**	0.56	0.57	0.38	0.86	—	—
	**4-II**	0.65	—	—	0.70	0.67	0.52
5	**5-I**	0.42	—	—	0.56	0.63	—
	**5-II**	0.40	0.42	—	0.59	—	—
6	**6-I**	0.37	—	—	0.44	0.55	0.48
	**6-II**	0.28	0.35	0.34	0.52	—	—
7	**7-I**	0.46	0.60	0.34	0.67	0.65	—
	**7-II**	0.54	0.41	—	0.60	0.40	0.48
8	**8-I**	0.59	0.47	ND	0.80	—	—
	**8-II**	0.61	—	—	0.77	0.63	ND

ND = not determined

19. ASIS of compounds 1-8 (ASIS ($\Delta\delta$): $\Delta\delta = \delta_{CDCl_3} - \delta_{C_6F_6}$).

Table S2. ASIS of compounds **1-8** (ASIS ($\Delta\delta$): $\Delta\delta = \delta_{CDCl_3} - \delta_{C_6F_6}$).

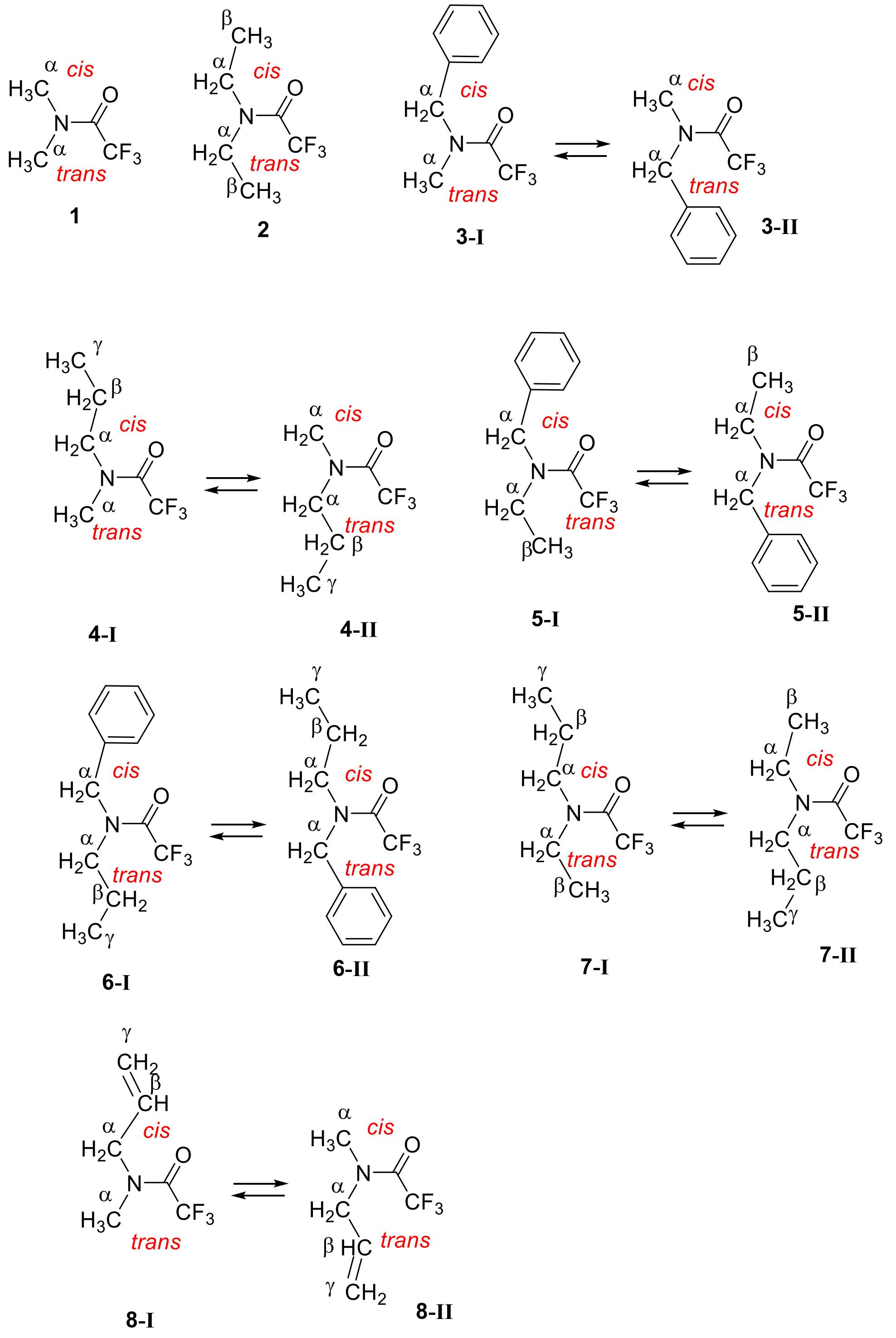

ASIS ($\Delta\delta$): $\Delta\delta = \delta_{CDCl_3} - \delta_{C_6F_6}$							
		cis			*trans*		
Entry	compounds	C^{α}	C^{β}	C^{γ}	C^{α}	C^{β}	C^{γ}
1	**1**	-0.13	—	—	-0.24	—	—
2	**2**	-0.12	-0.12	—	-0.24	-0.24	—
3	**3-I**	-0.04	—	—	-0.23	—	—
	**3-II**	-0.08	—	—	-0.15	—	—
4	**4-I**	-0.10	-0.14	-0.16	-0.23	—	—
	**4-II**	-0.11	—	—	-0.22	-0.30	-0.24
5	**5-I**	-0.03	—	—	-0.20	-0.27	—
	**5-II**	-0.07	-0.13	—	-0.15	—	—
6	**6-I**	-0.02	—	—	-0.18	-0.27	-0.24
	**6-II**	-0.06	-0.13	-0.15	-0.13	—	—
7	**7-I**	-0.11	-0.12	-0.15	-0.23	-0.24	—
	**7-II**	-0.11	-0.13	—	-0.22	-0.27	-0.23
8	**8-I**	-0.07	-0.16	ND	-0.23	—	—
	**8-II**	-0.10	—	—	-0.19	-0.24	ND

ND = not determined

20. NMR spectra of 4.

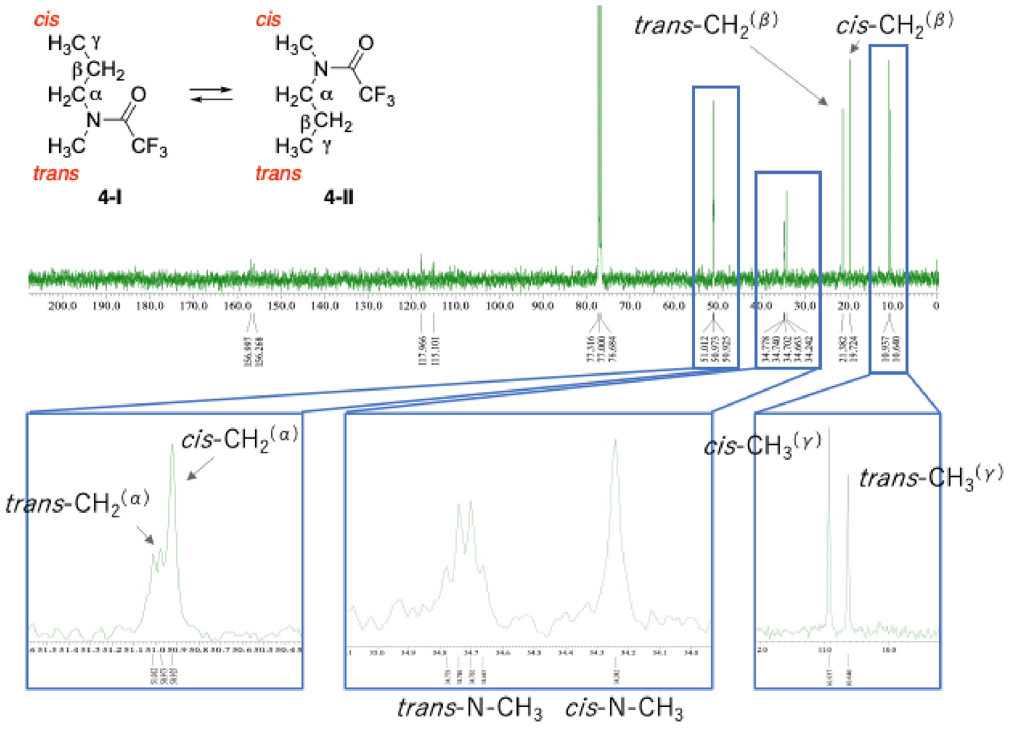

^{13}C, $CDCl_3$, 100 MHz

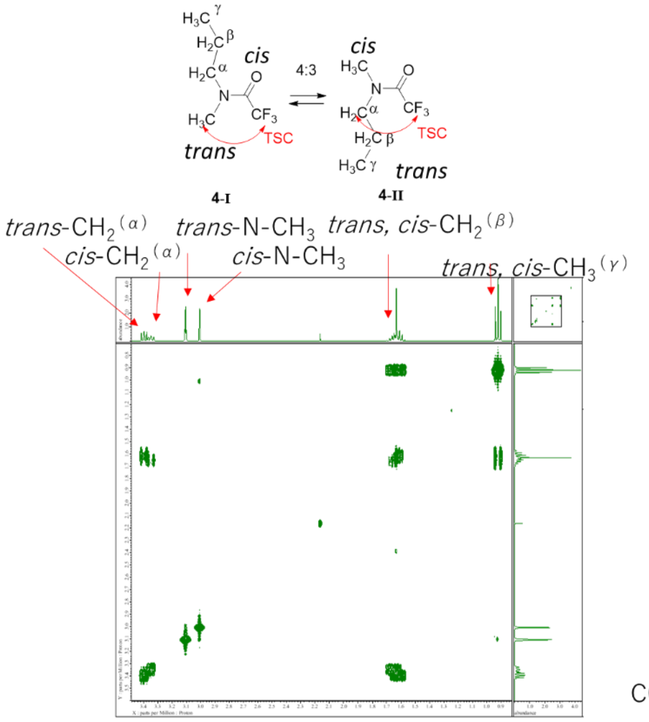

COSY, $CDCl_3$, 400 MHz

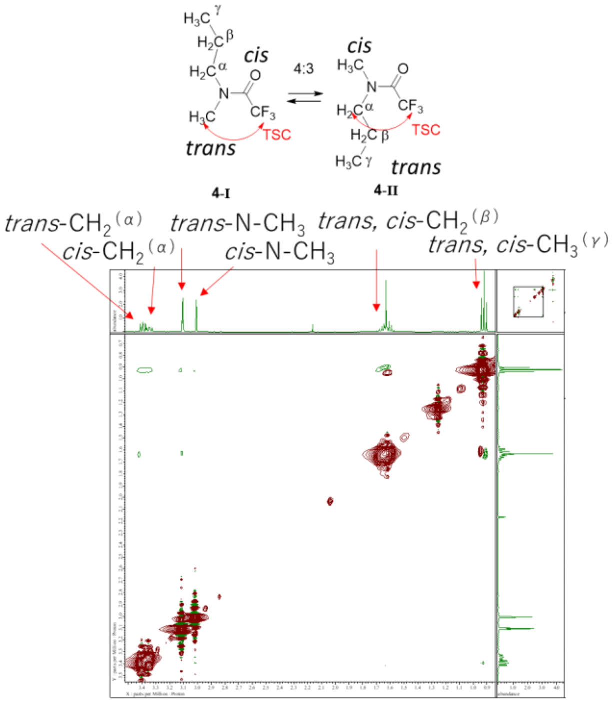

NOESY, $CDCl_3$, 400 MHz

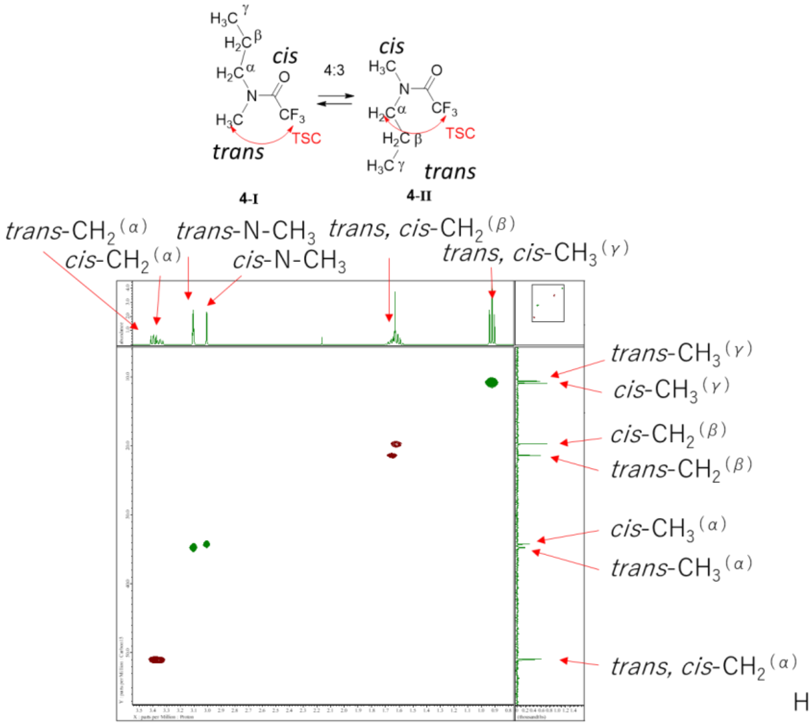

HSQC, 400 MHz, In $CDCl_3$

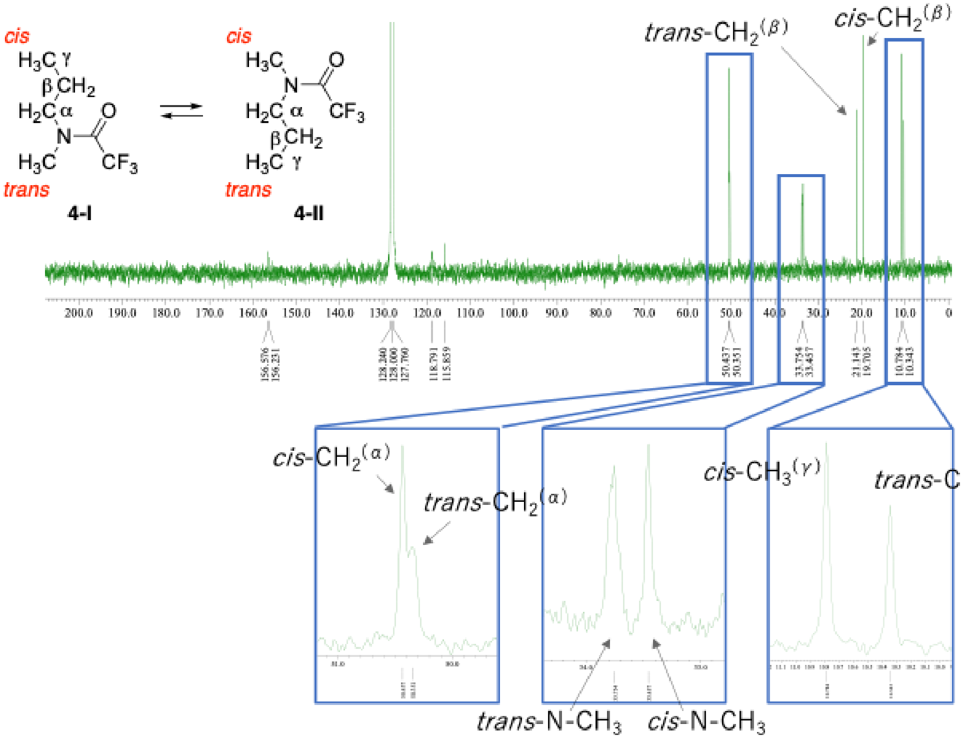

^{13}C, C_6D_6, 100 MHz

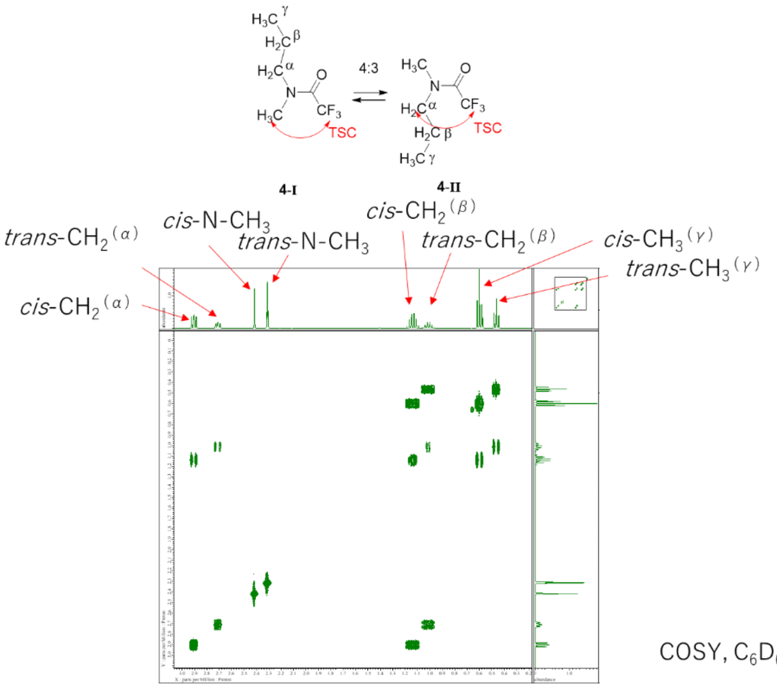

COSY, C_6D_6, 400 MHz

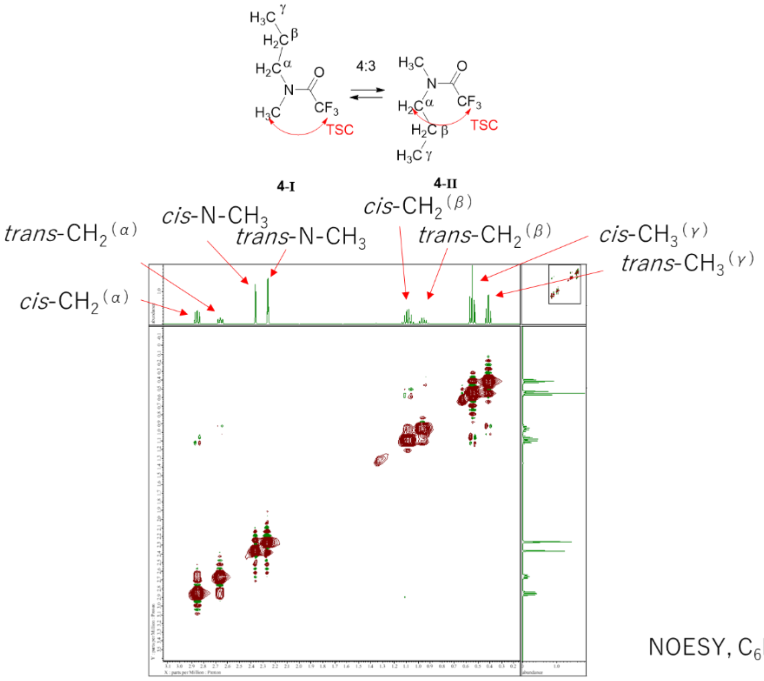

NOESY, C_6D_6, 400 MHz

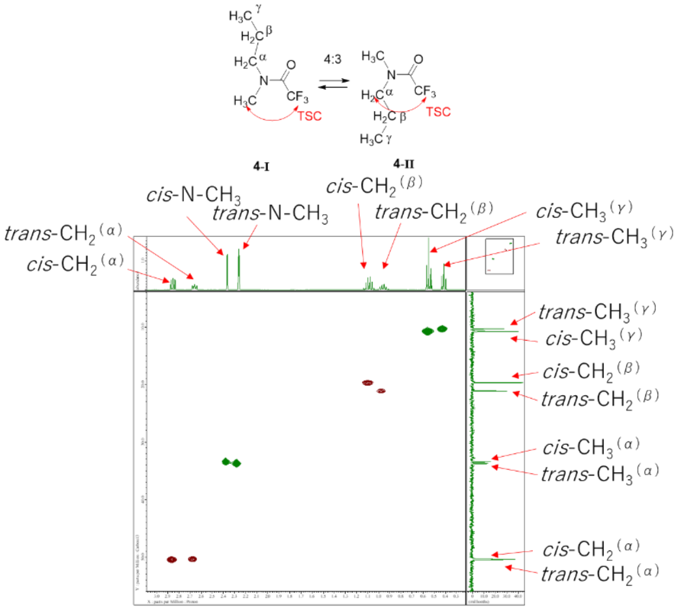

HSQC, C_6D_6, 400 MHz

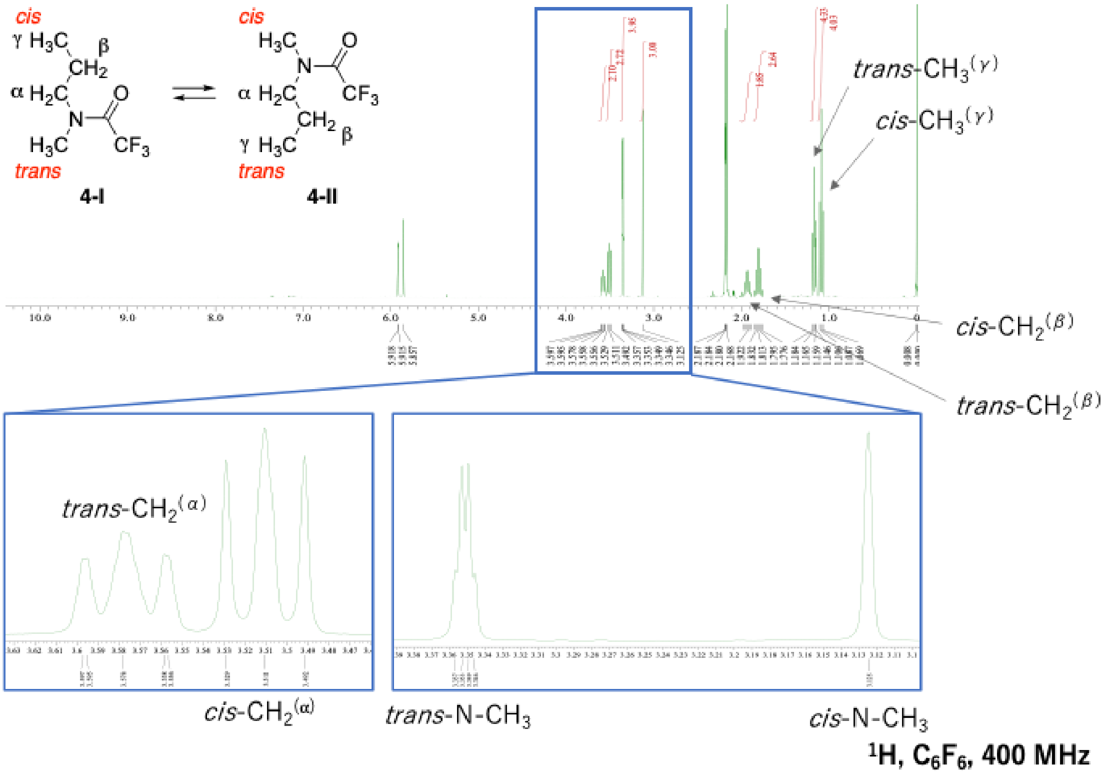
cis
trans
4-I
4-II
trans-CH3(γ)
cis-CH3(γ)
cis-CH2(β)
trans-CH2(β)
trans-CH2(α)
cis-CH2(α)
trans-N-CH3
cis-N-CH3
1H, C6F6, 400 MHz

21. NMR spectra of 5.

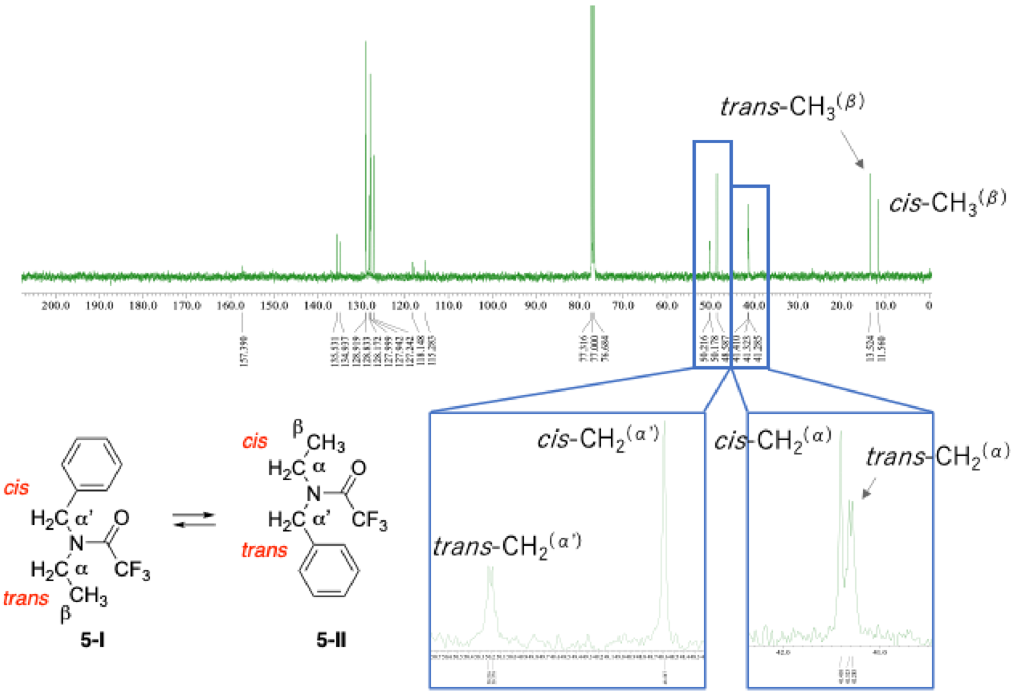

^{13}C, $CDCl_3$, 100 MHz

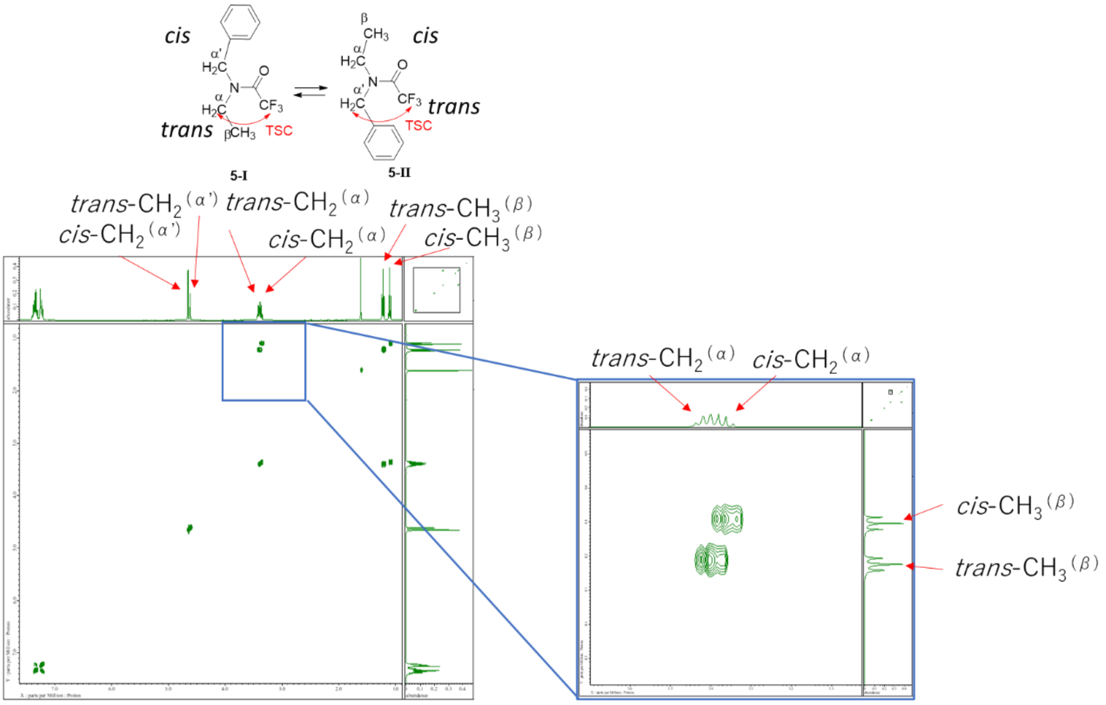

COSY, $CDCl_3$, 400 MHz

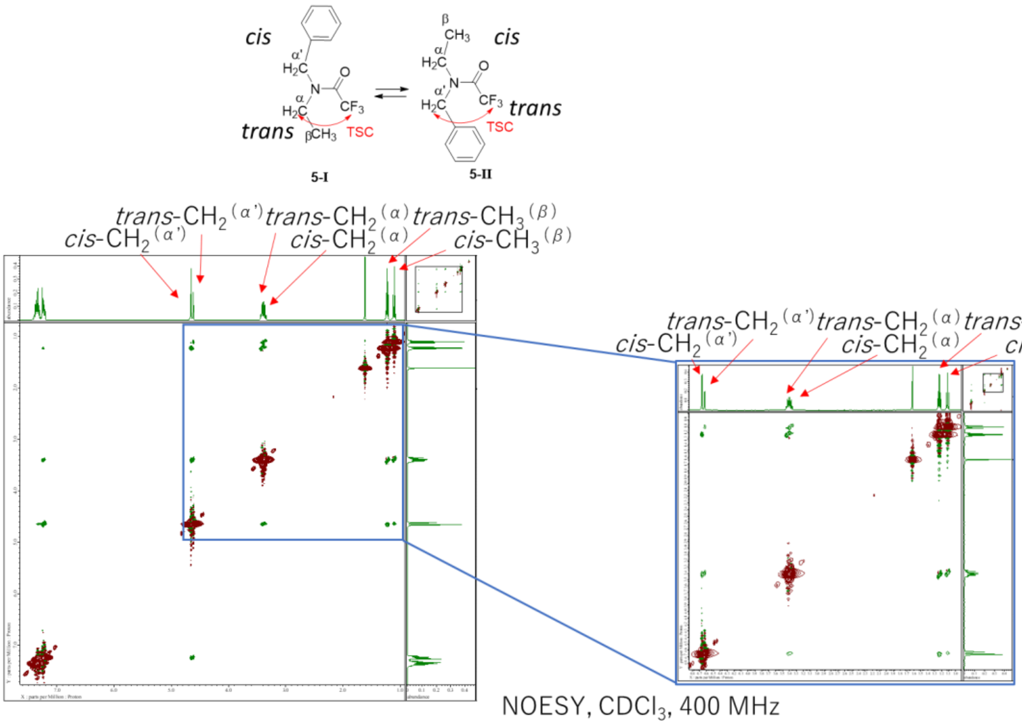

NOESY, $CDCl_3$, 400 MHz

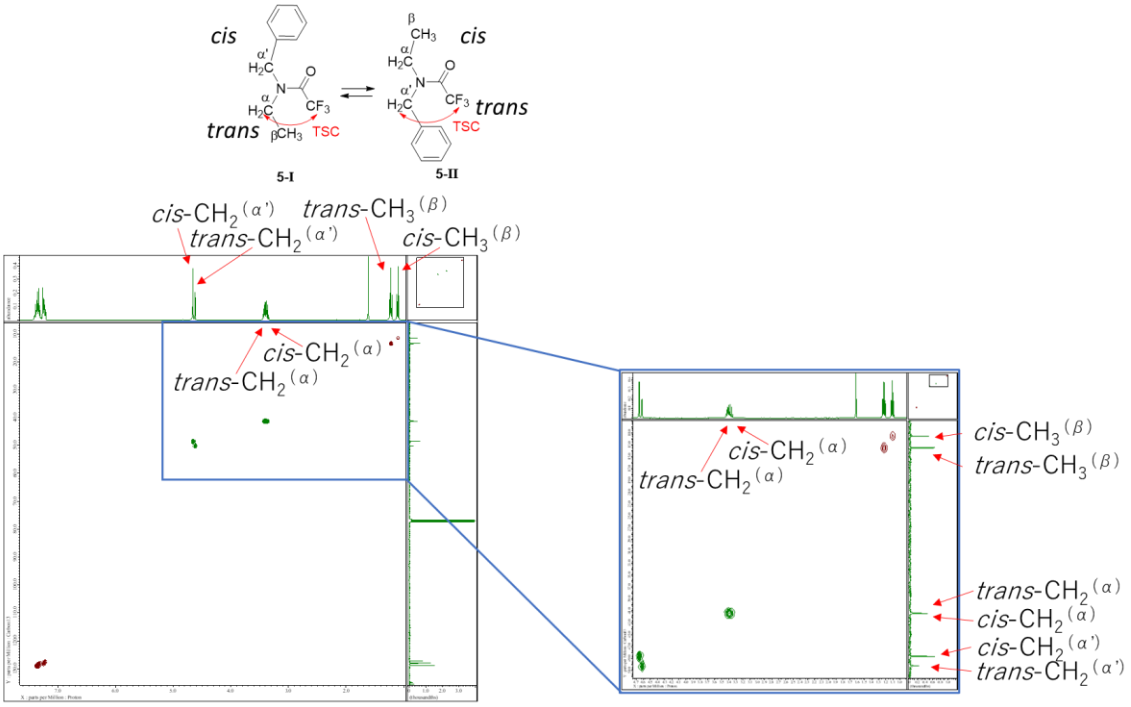

HSQC, $CDCl_3$, 400 MHz

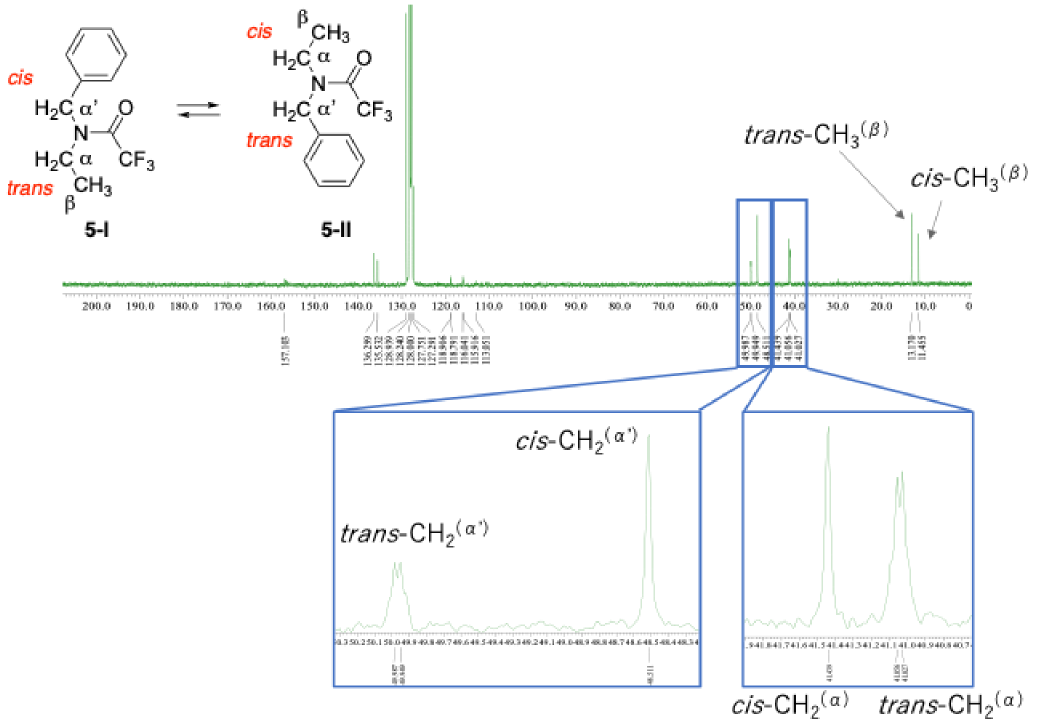

^{13}C, C_6D_6, 100 MHz

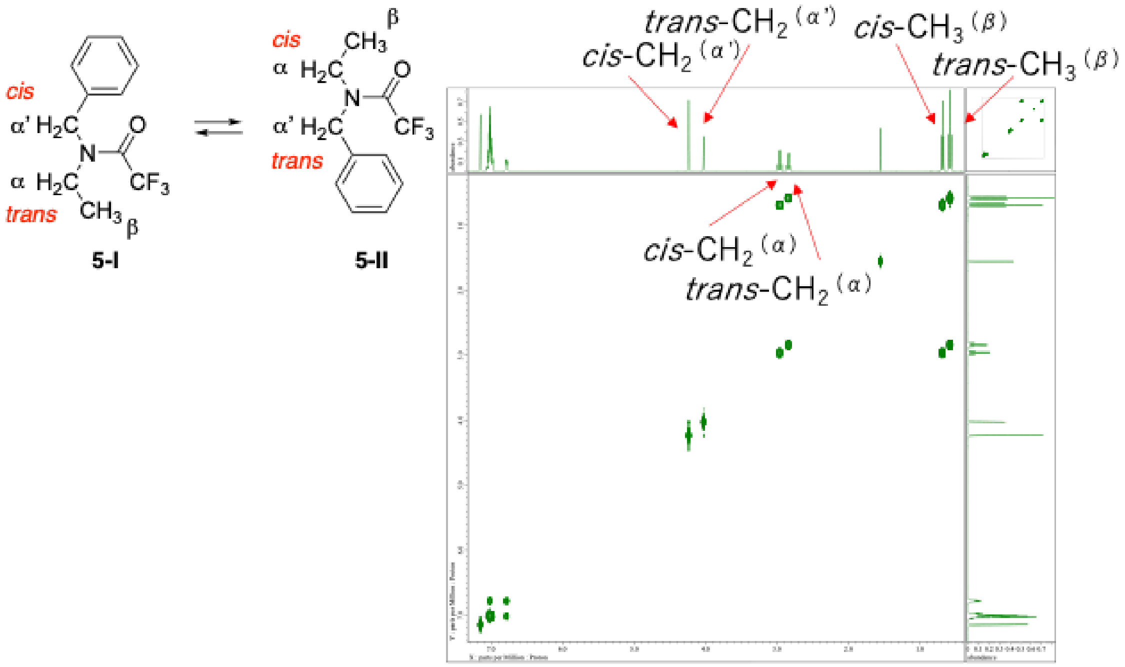

COSY, C_6D_6, 400 MHz

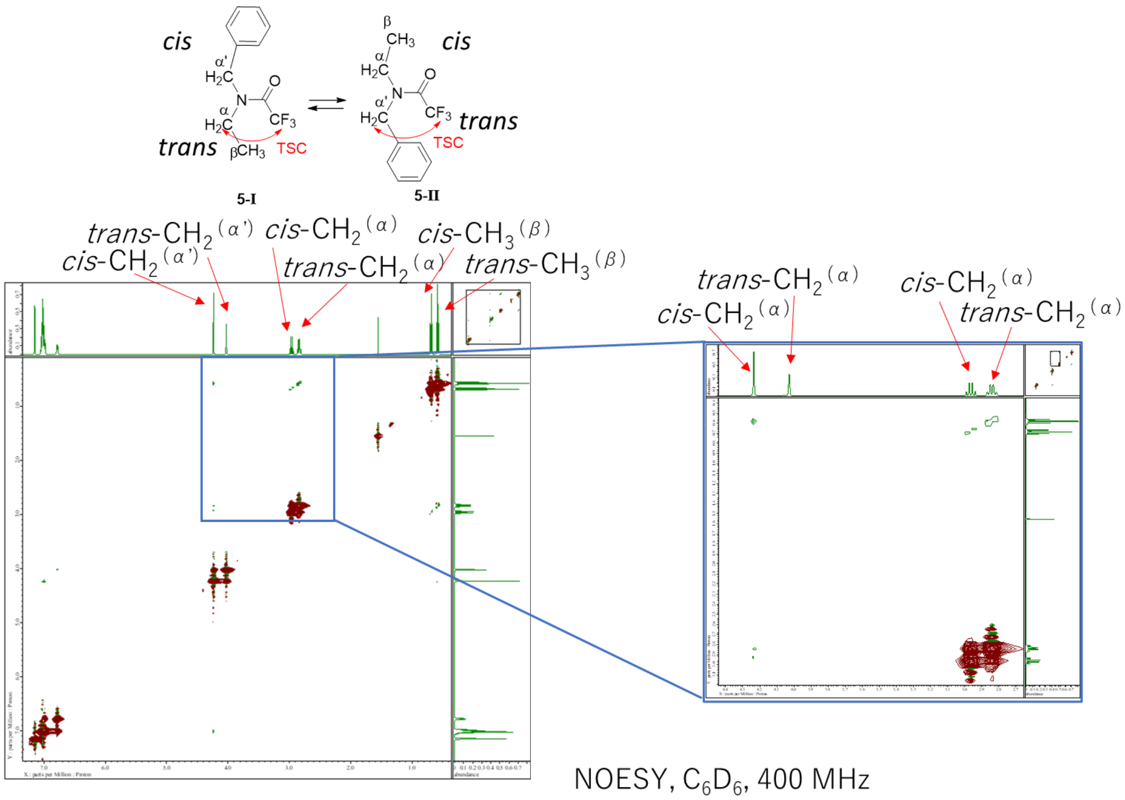

NOESY, C_6D_6, 400 MHz

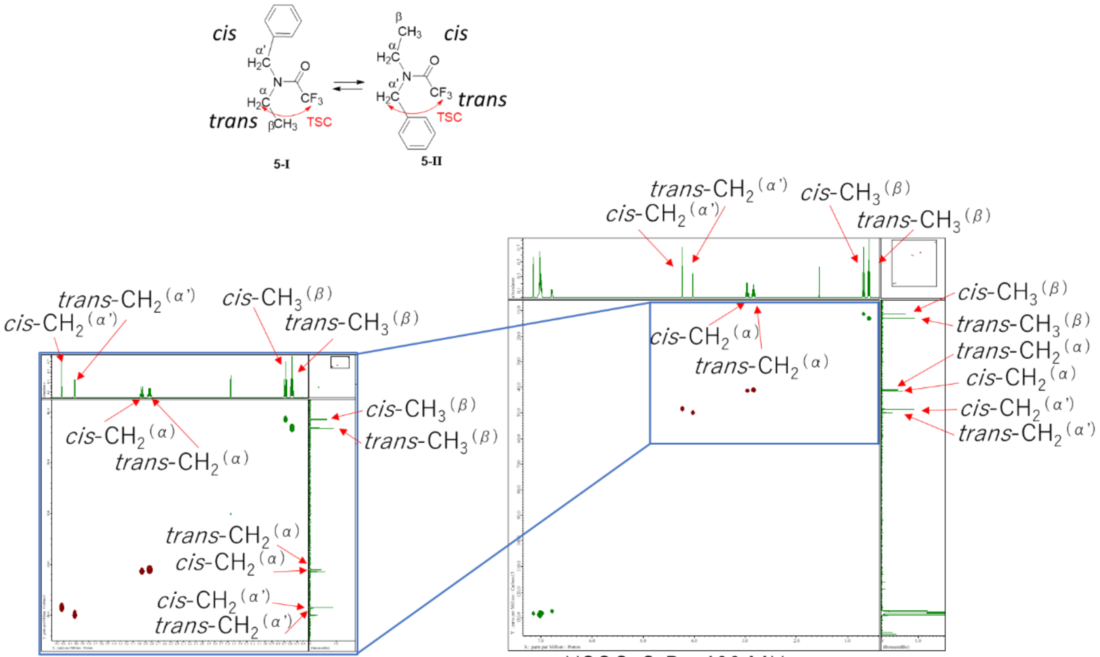

HSQC, C_6D_6, 400 MHz

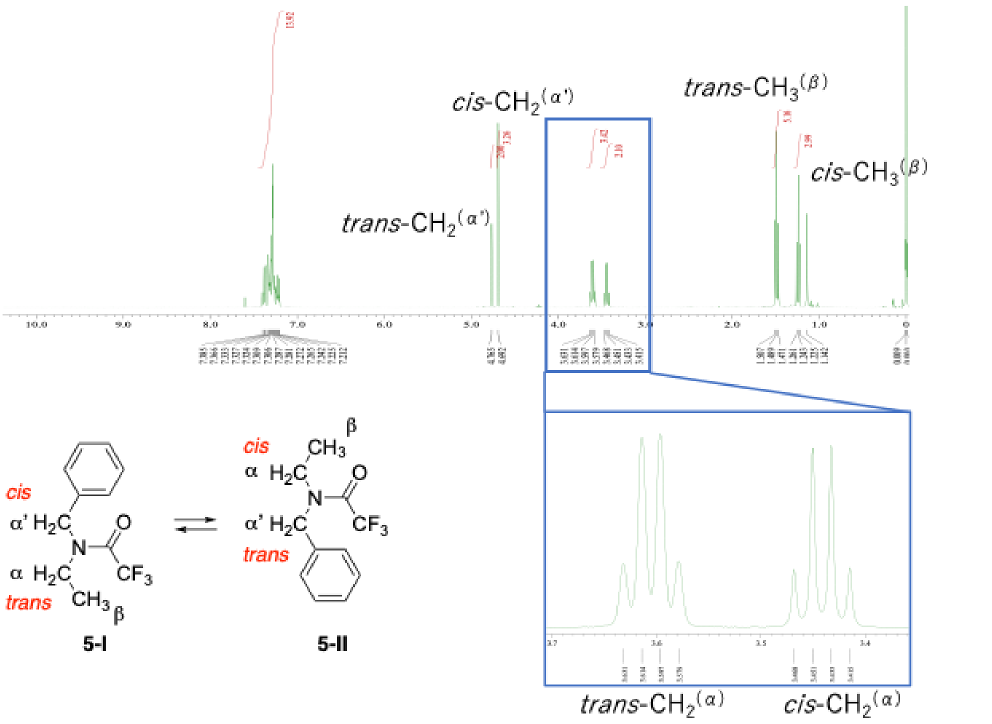

^{1}H, C_6F_6, 400 MHz

22. NMR spectra of 6.

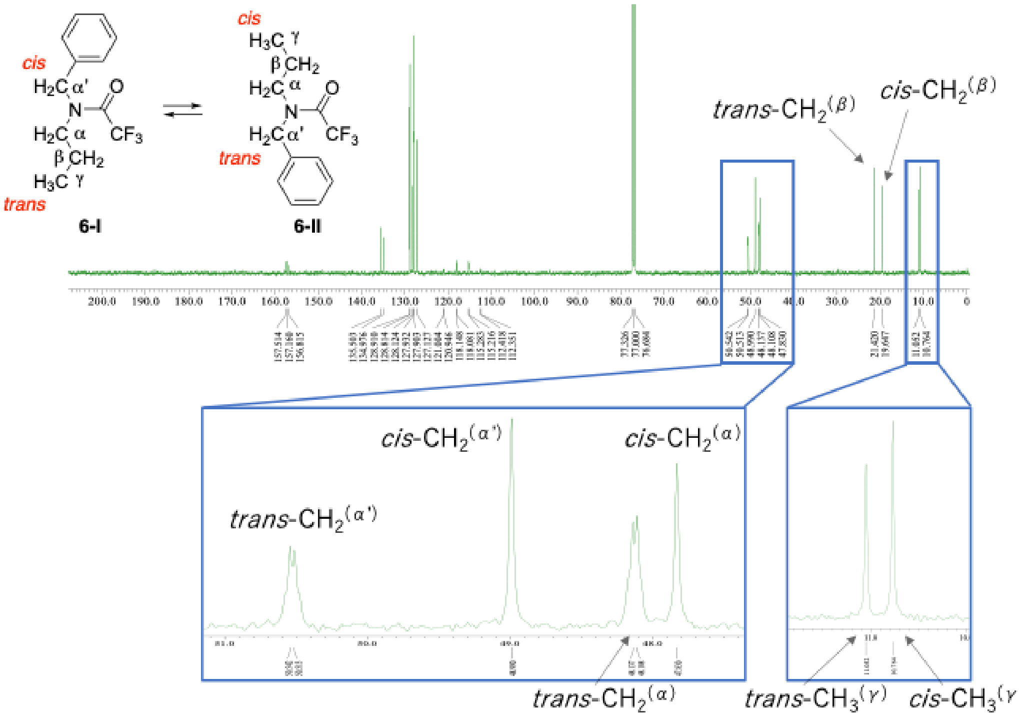

^{13}C, $CDCl_3$, 100 MHz

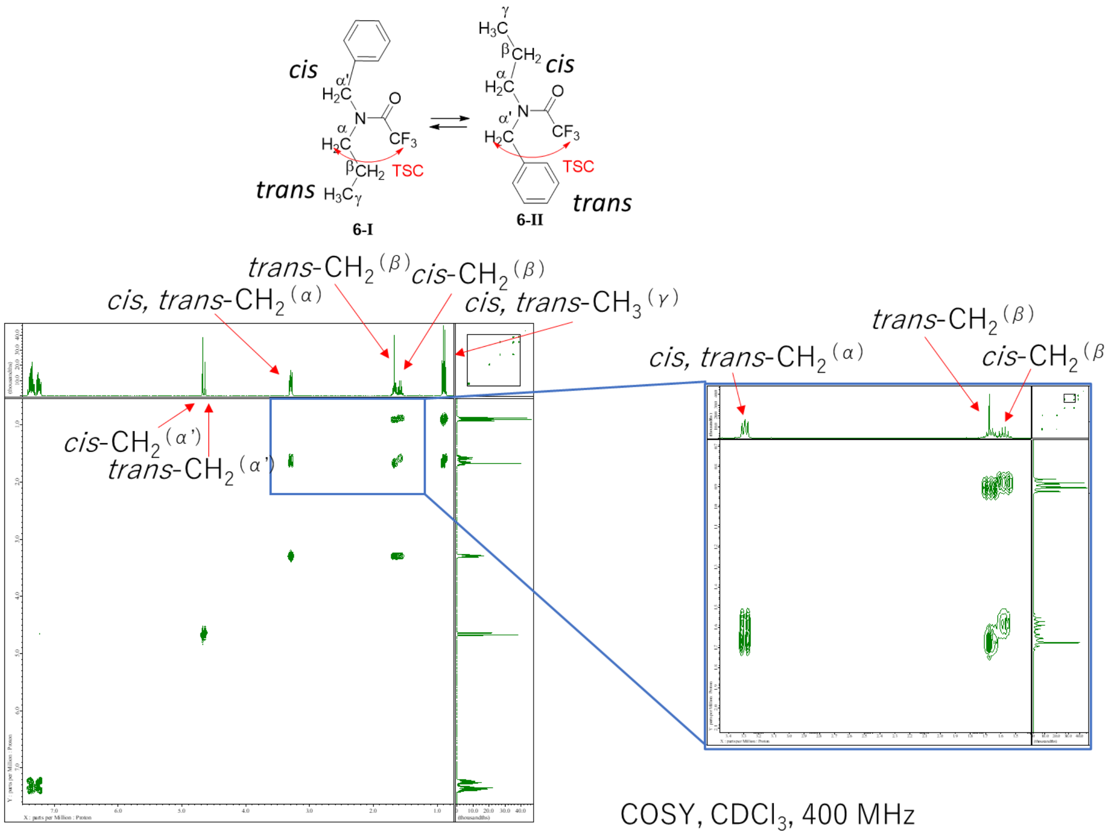

COSY, $CDCl_3$, 400 MHz

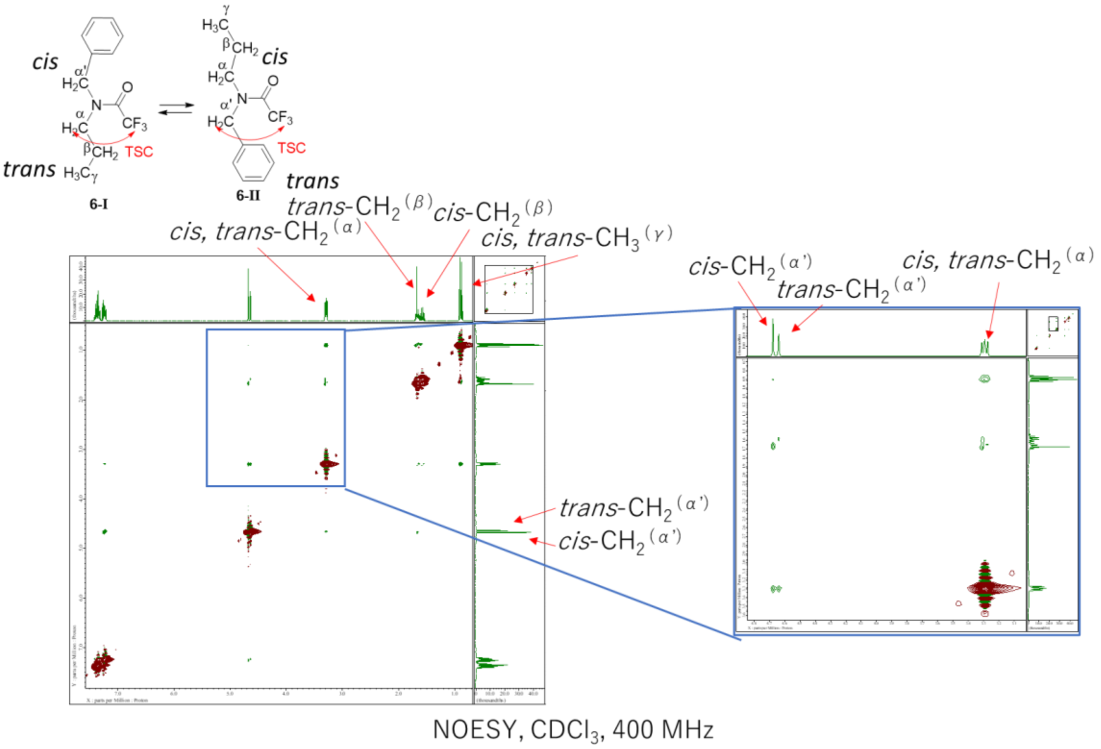

NOESY, $CDCl_3$, 400 MHz

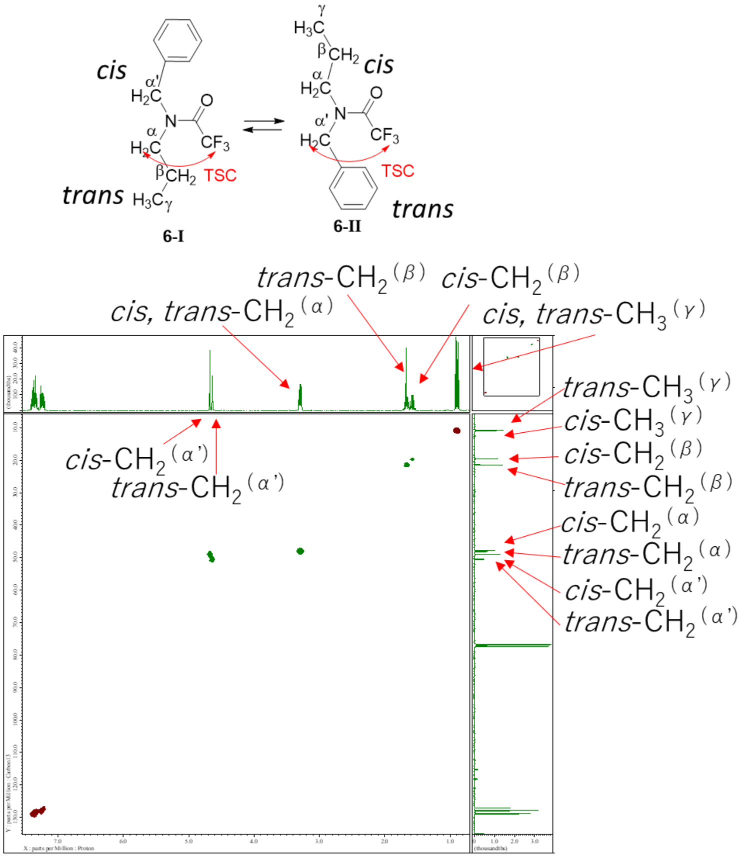

HSQC, $CDCl_3$, 400 MHz

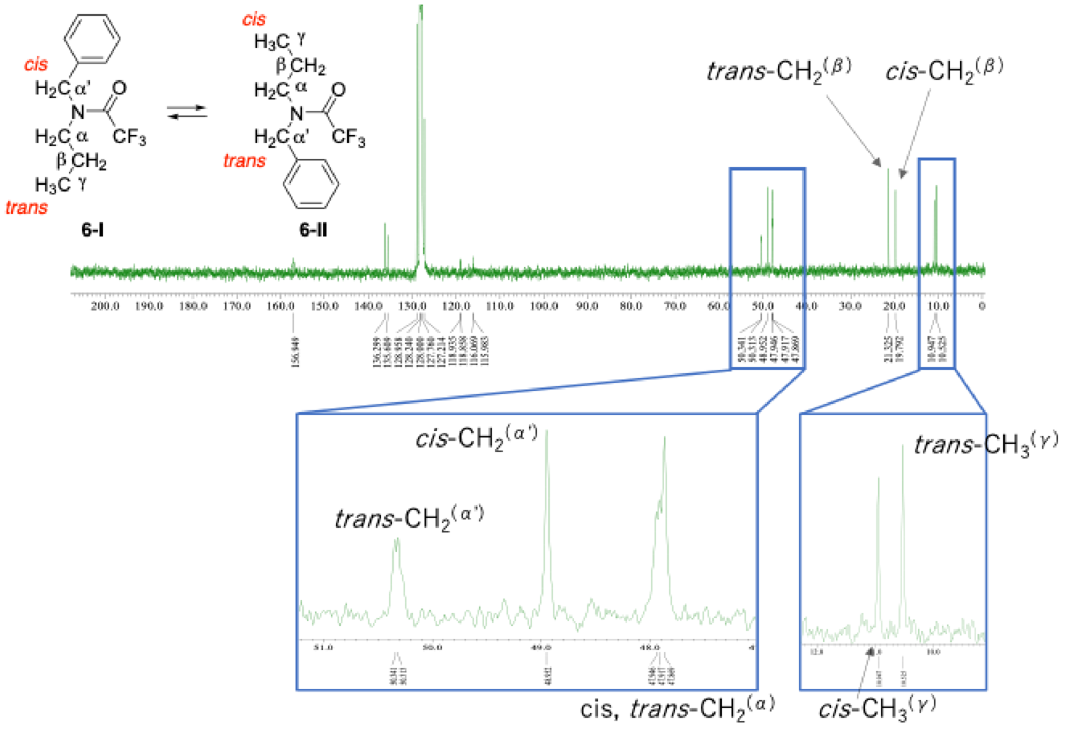

^{13}C, C_6D_6, 100 MHz

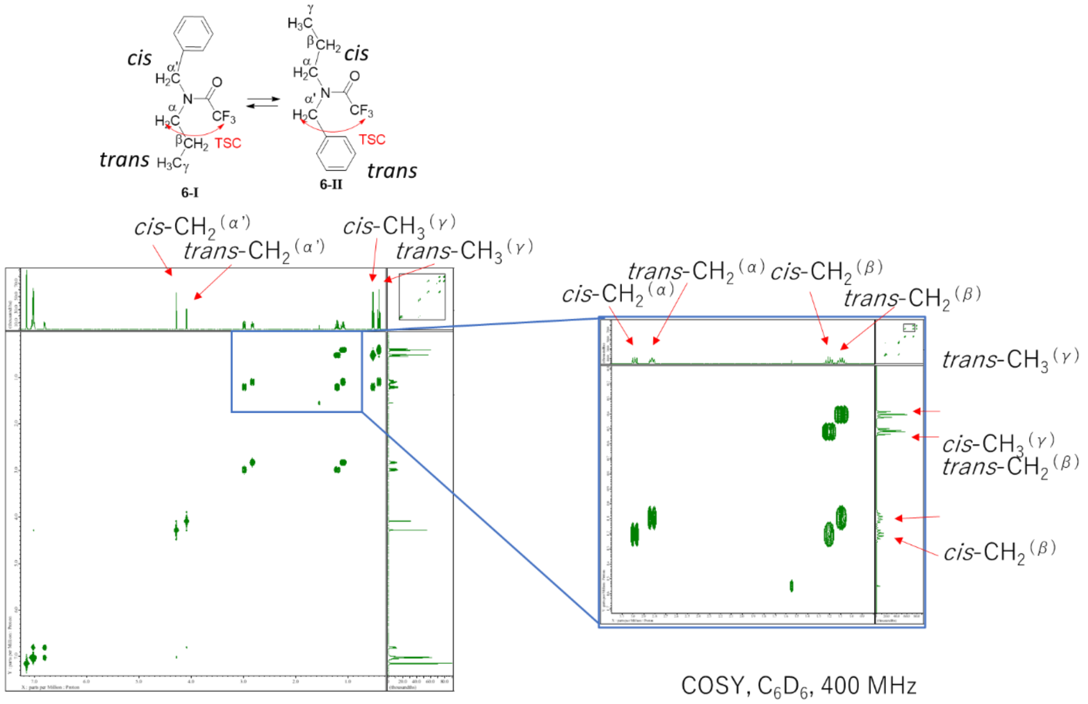

COSY, C_6D_6, 400 MHz

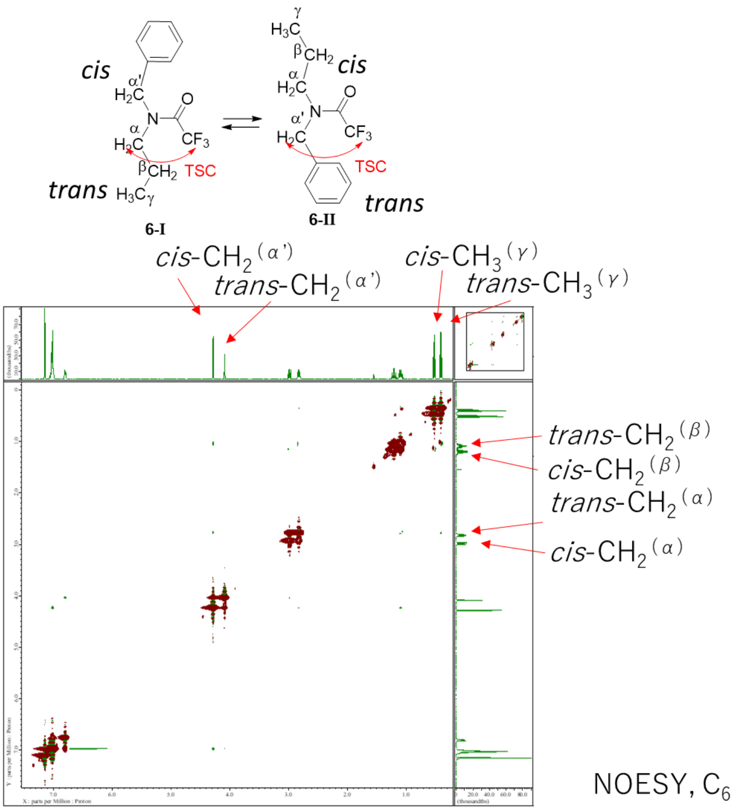

NOESY, C_6D_6, 400 MHz

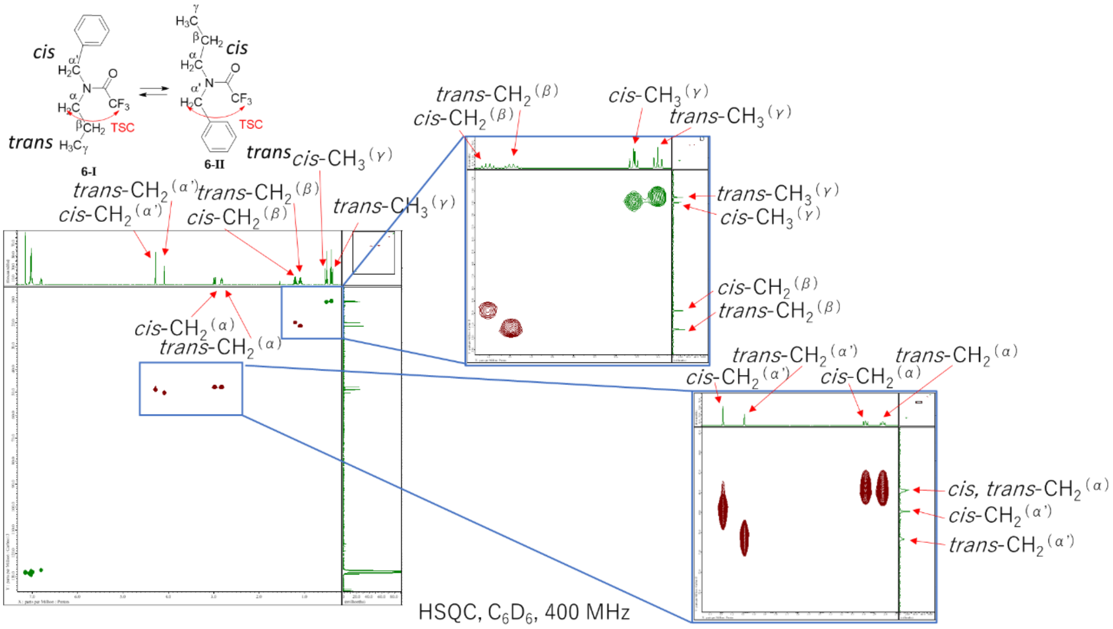

HSQC, C_6D_6, 400 MHz

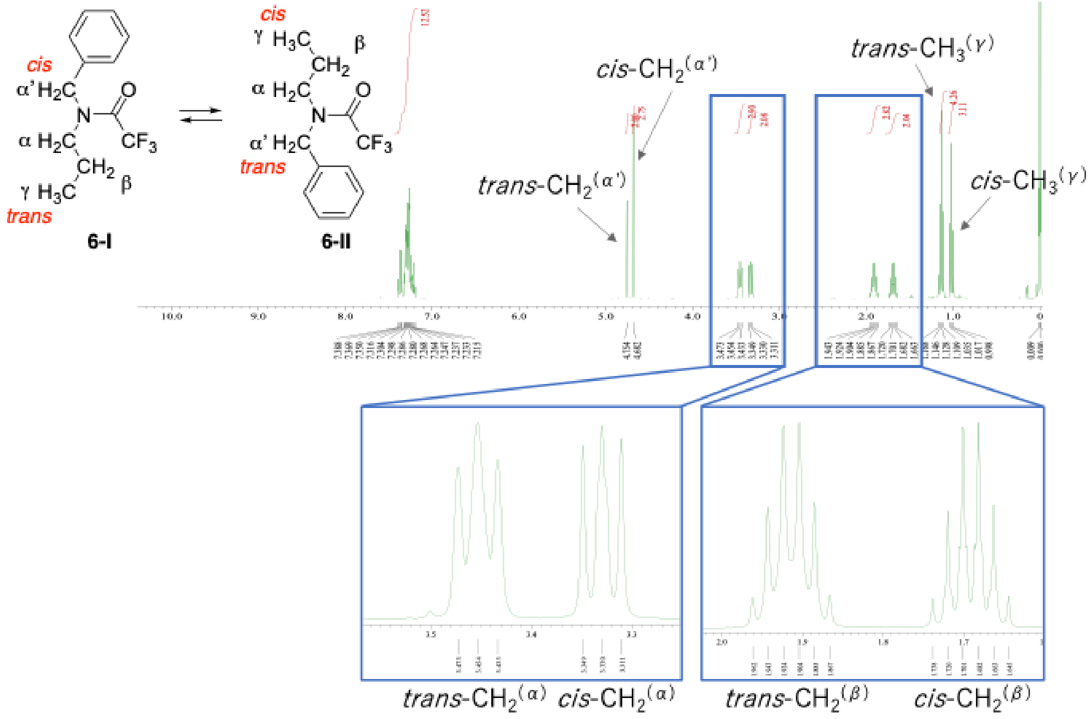

^{1}H, C_6F_6, 400 MHz

23. NMR spectra of 7.

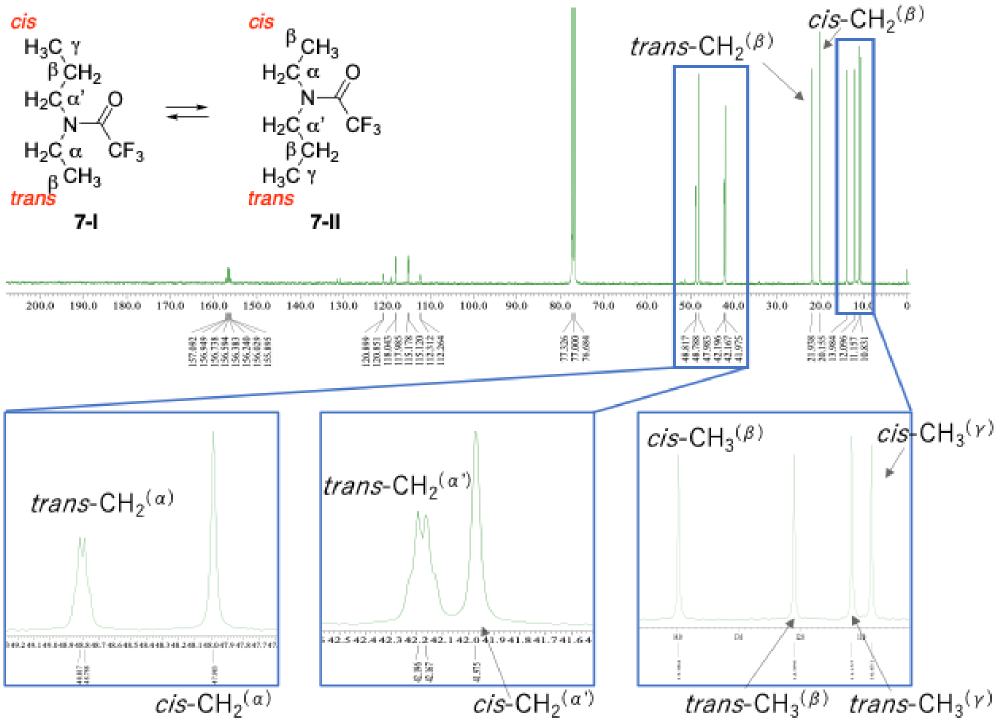

^{13}C, CDCl$_3$, 100 MHz

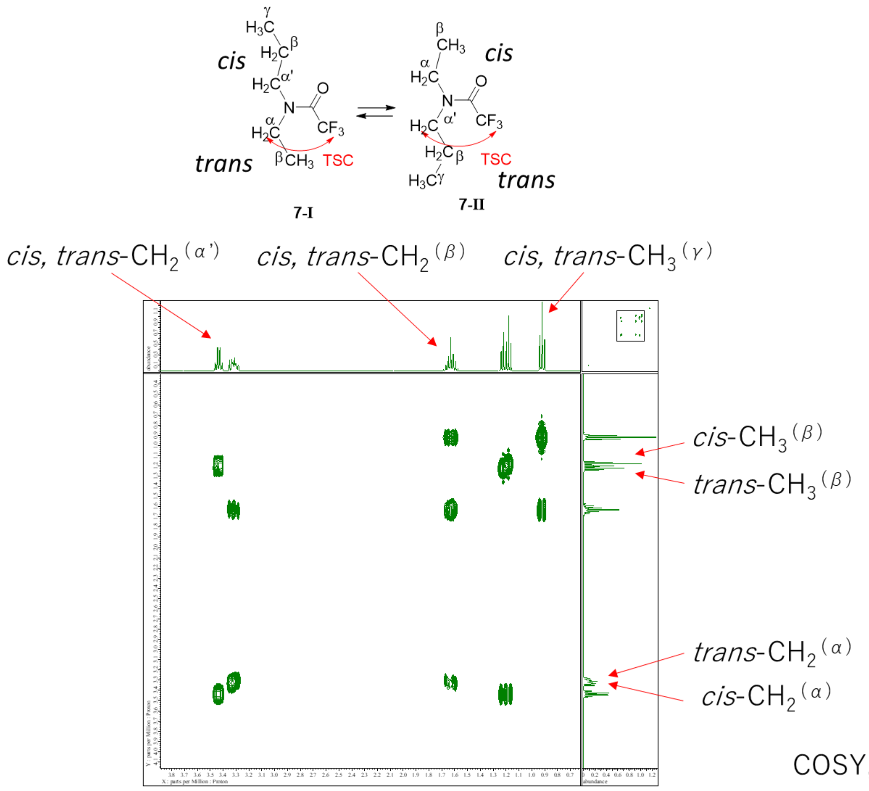

COSY, CDCl$_3$, 400 MHz

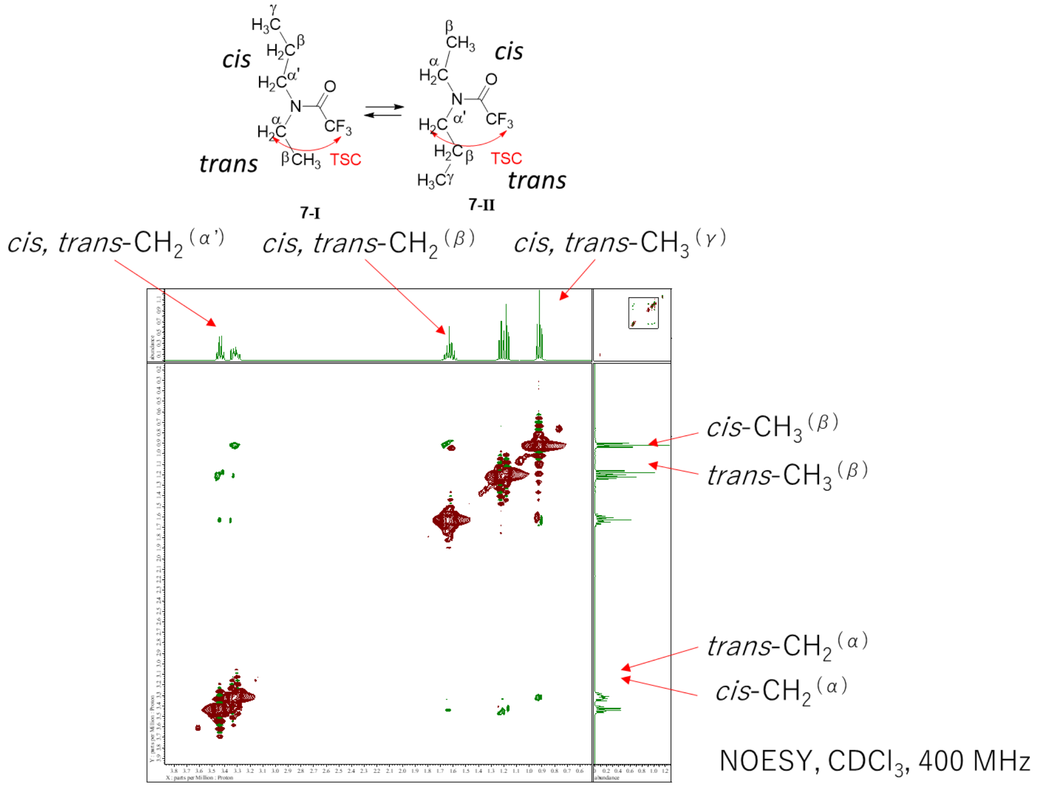
cis
trans
TSC
7-I
7-II
cis, trans-CH2 (α')
cis, trans-CH2 (β)
cis, trans-CH3 (γ)
cis-CH3 (β)
trans-CH3 (β)
trans-CH2 (α)
cis-CH2 (α)
NOESY, CDCl3, 400 MHz

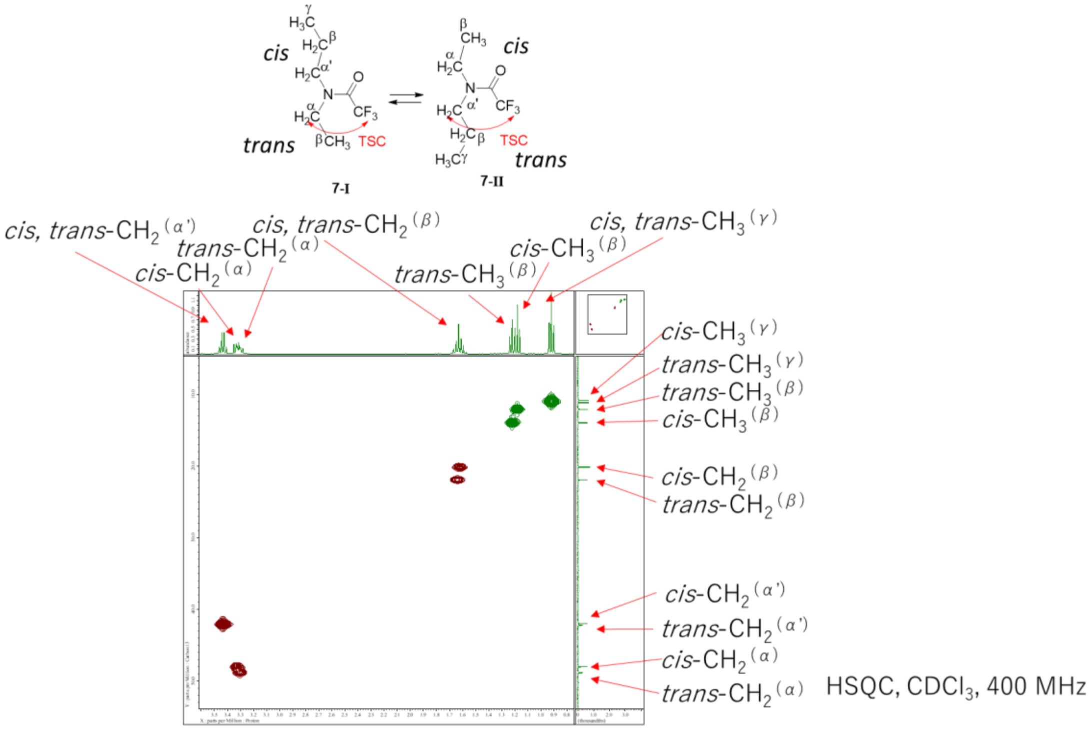
cis
trans
TSC
7-I
7-II
cis, trans-CH2 (α')
cis, trans-CH2 (β)
cis, trans-CH3 (γ)
trans-CH2 (α)
cis-CH2 (α)
cis-CH3 (β)
trans-CH3 (β)
cis-CH3 (γ)
trans-CH3 (γ)
trans-CH3 (β)
cis-CH3 (β)
cis-CH2 (β)
trans-CH2 (β)
cis-CH2 (α')
trans-CH2 (α')
cis-CH2 (α)
trans-CH2 (α)
HSQC, CDCl3, 400 MHz

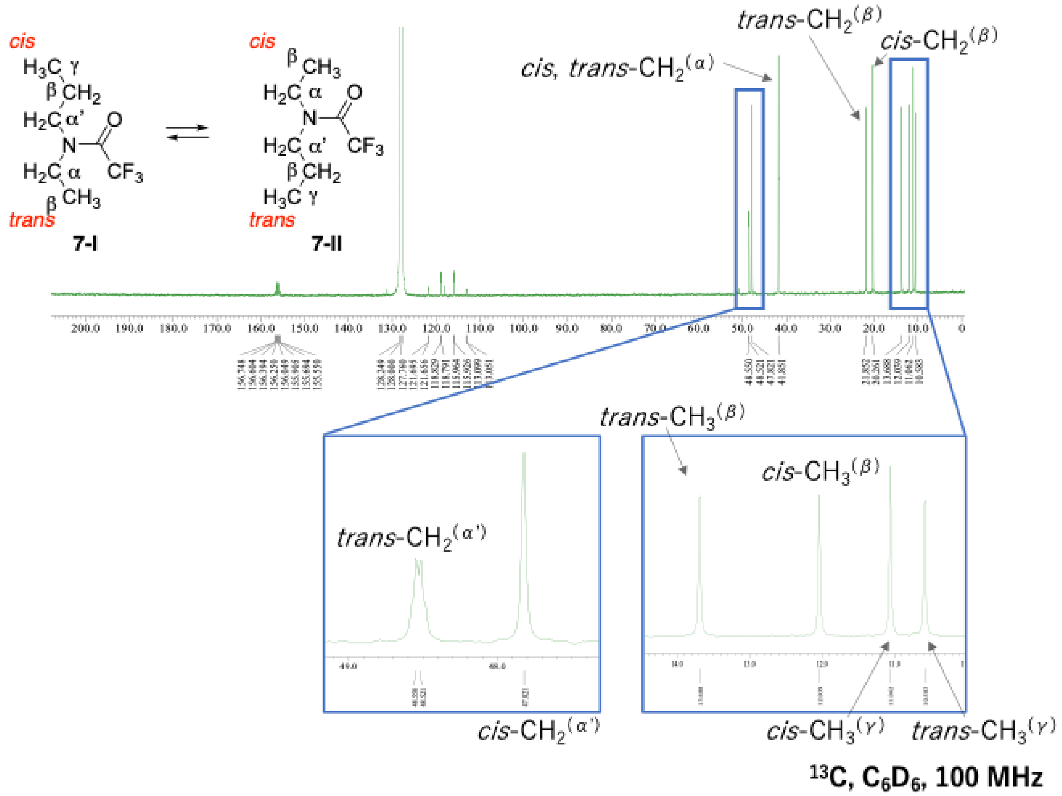

cis
trans
7-I
7-II
TSC
trans-CH2(β)
cis-CH2(β)
cis, trans-CH2(α)
trans-CH3(β)
cis-CH3(β)
trans-CH2(α')
cis-CH2(α')
cis-CH3(γ)
trans-CH3(γ)
13C, C6D6, 100 MHz

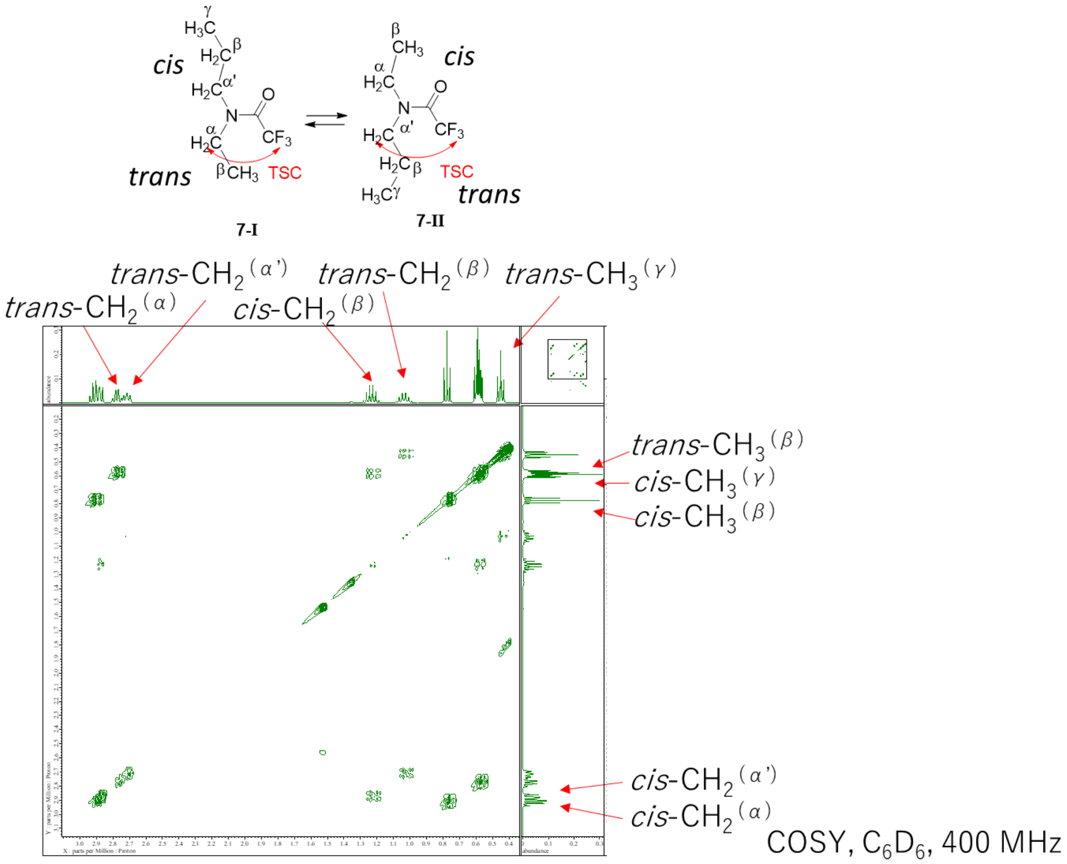

cis
trans
7-I
7-II
TSC
trans-CH2(α')
trans-CH2(β)
trans-CH3(γ)
trans-CH2(α)
cis-CH2(β)
trans-CH3(β)
cis-CH3(γ)
cis-CH3(β)
cis-CH2(α')
cis-CH2(α)
COSY, C6D6, 400 MHz

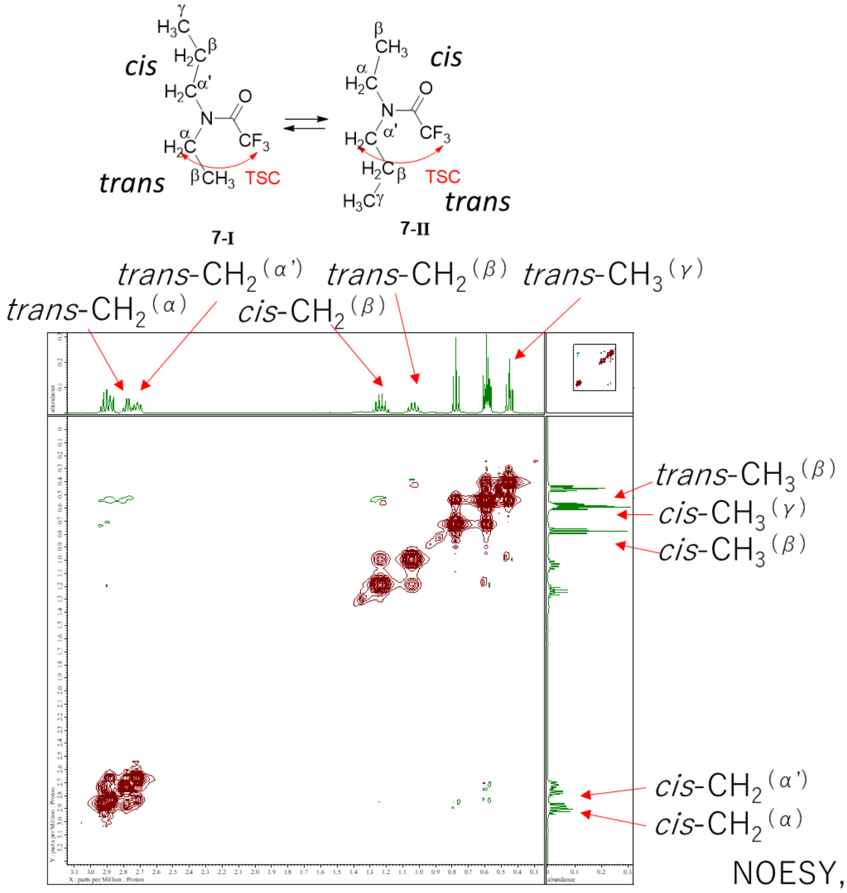

NOESY, C_6D_6, 400 MHz

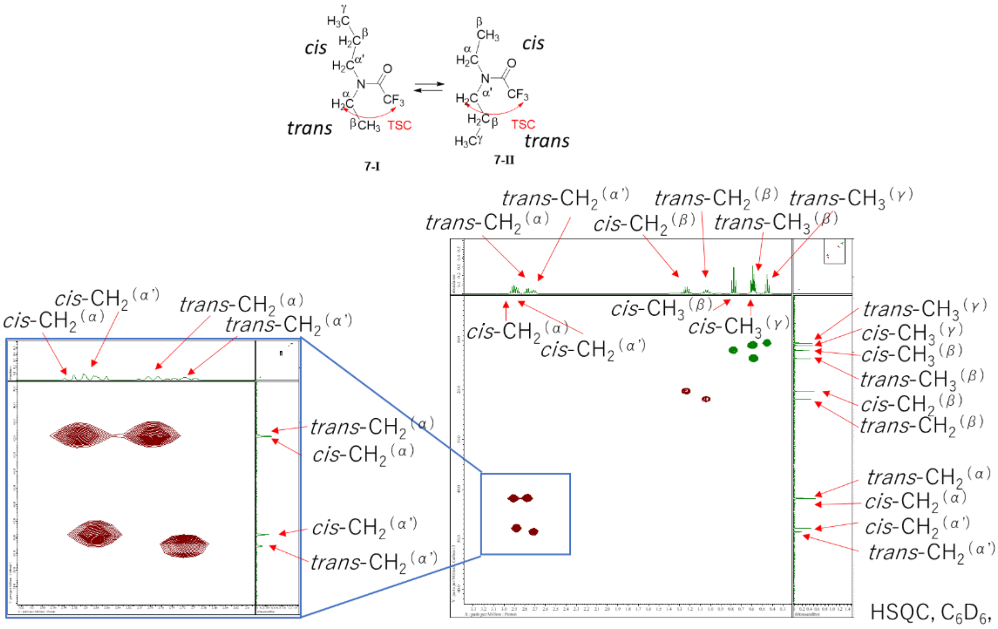

HSQC, C_6D_6, 400 MHz

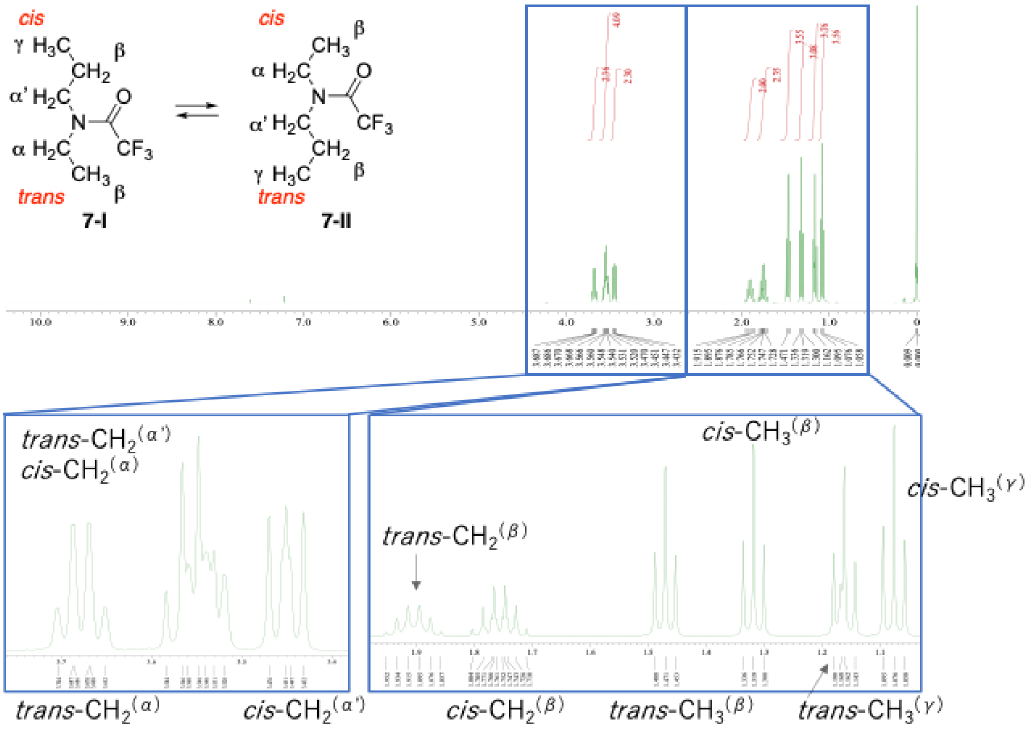

^{1}H, C_6F_6, 400 MHz

24. NMR spectra of 8.

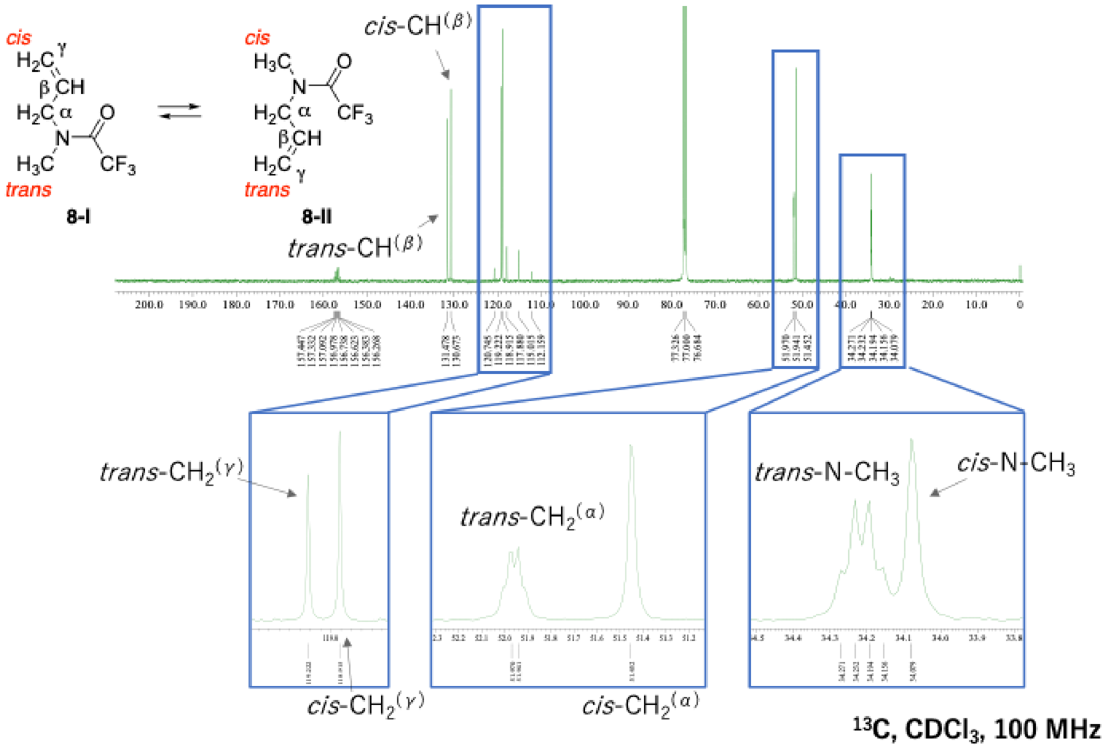

^{13}C, $CDCl_3$, 100 MHz

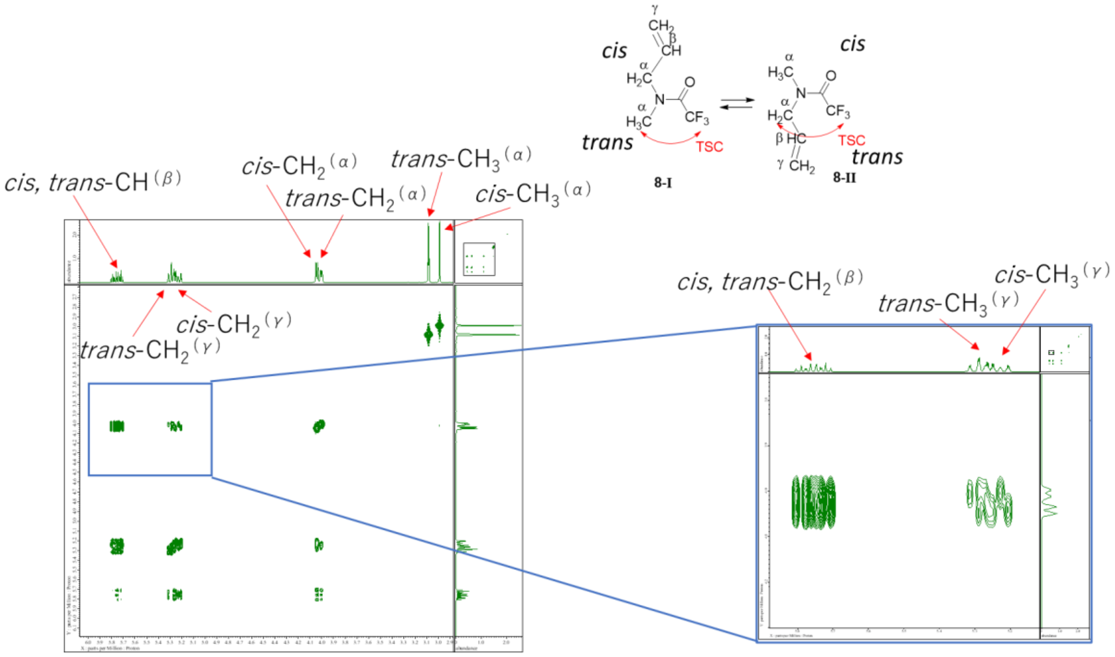

COSY, $CDCl_3$, 400 MHz

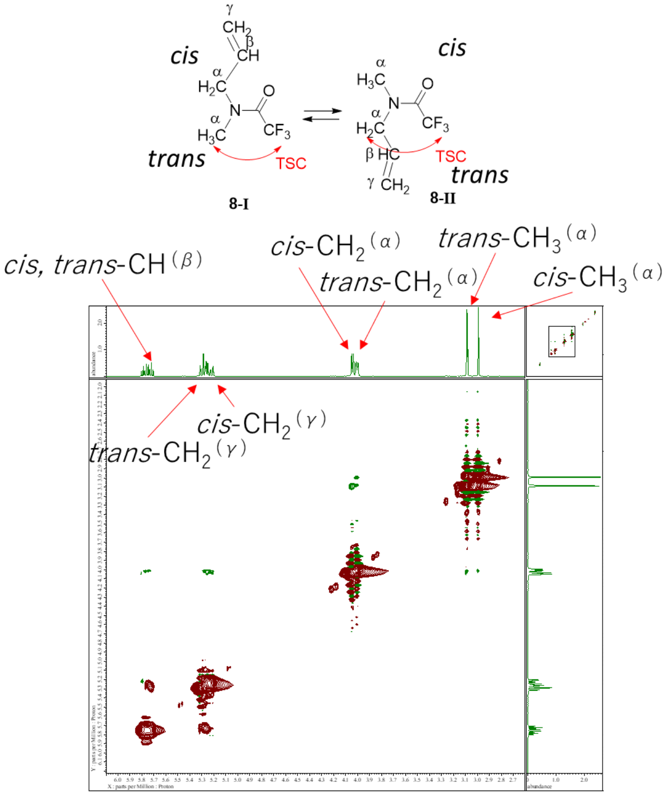

NOESY, $CDCl_3$, 400 MHz

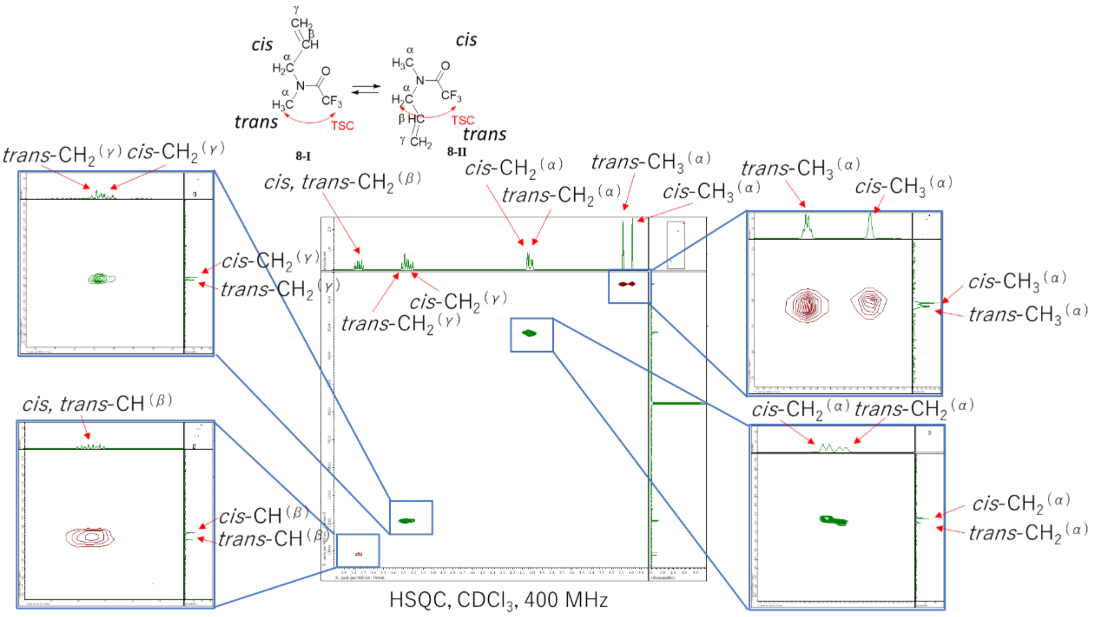

HSQC, $CDCl_3$, 400 MHz

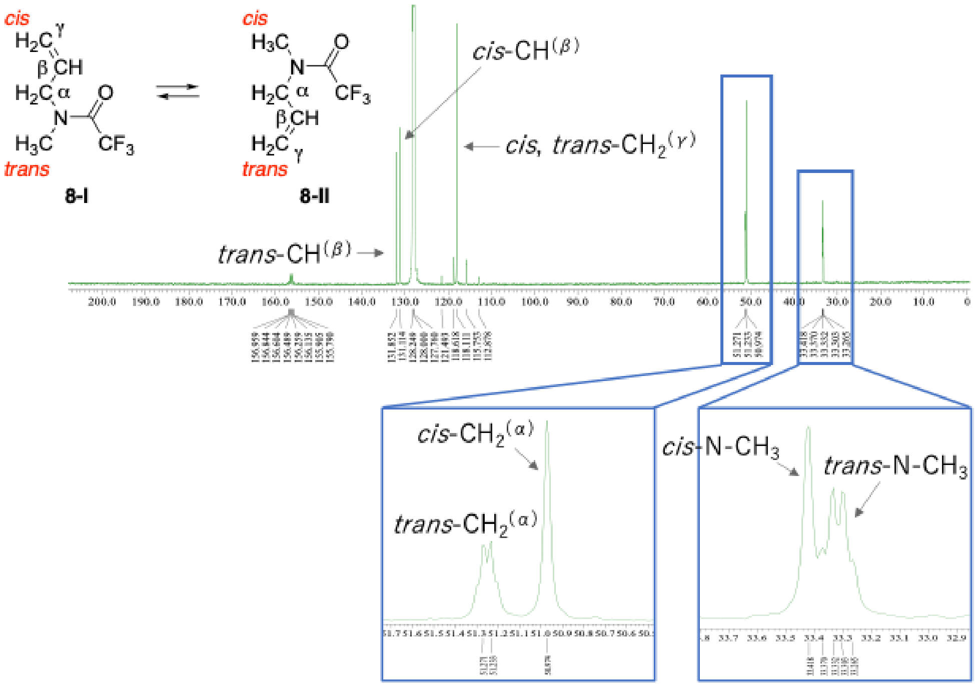

^{13}C, C_6D_6, 100 MHz

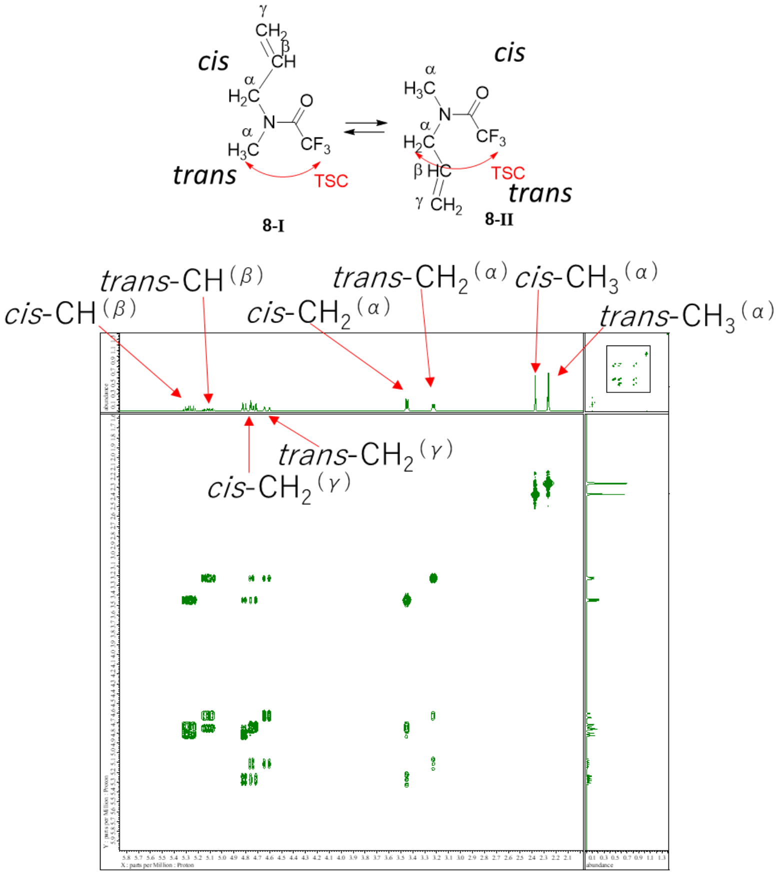

COSY, C_6D_6, 400 MHz

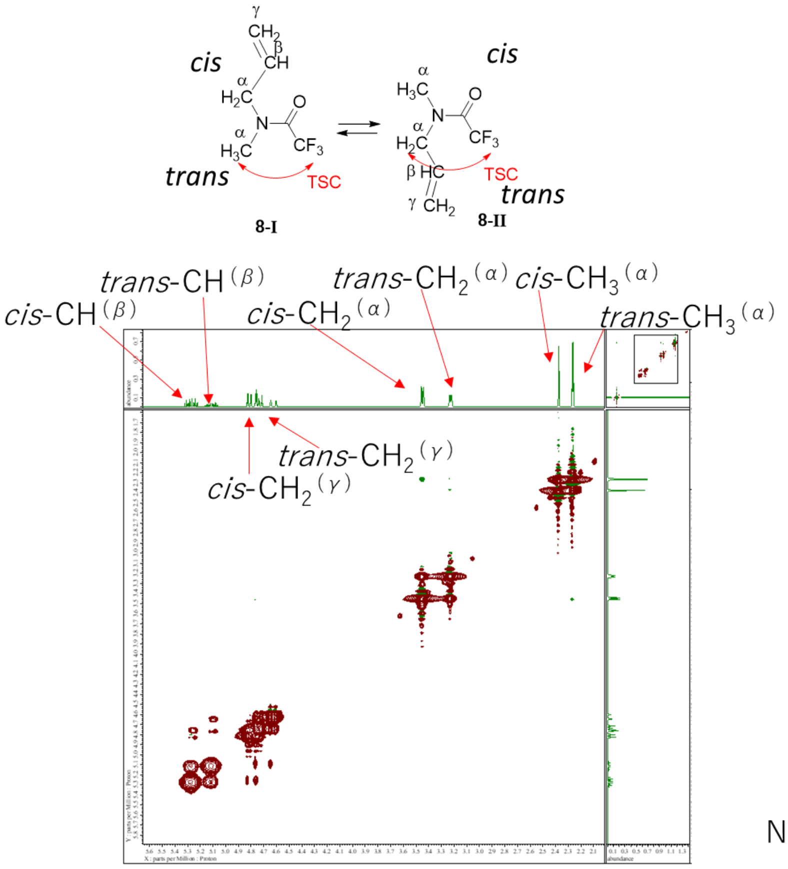

NOESY, C_6D_6, 400 MHz

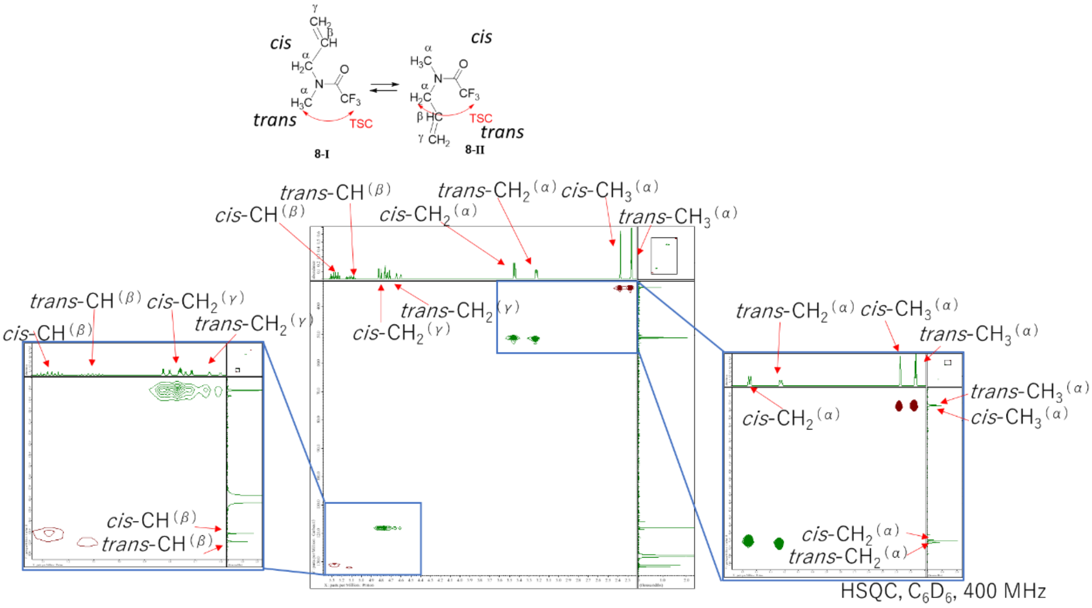

HSQC, C_6D_6, 400 MHz

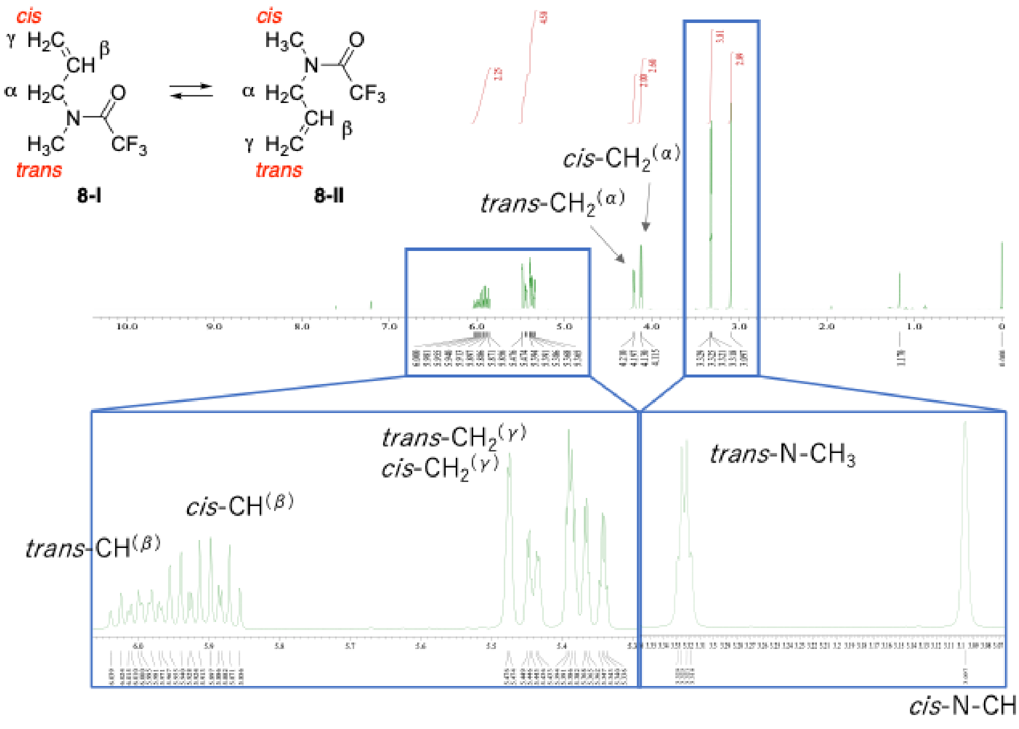

^{1}H, C_6F_6, 400 MHz

25. Calculation method.

Compounds **1**, **5-II** and **6-II** were constructed on Spartan’20 (Wavefunction, Inc., Irvine, CA, USA) and subjected to a conformational search with molecular mechanics MMFF94, with which the program is equipped, using a threshold of 40 kJ/mol from the global minimum conformer and successive conformer narrowing based on HF/3-21G (threshold: 40 kJ/mol) and ωB97X-D/6-31G(d) (threshold: 10 kJ/mol) to yield one stable conformer (M001) for **1** and four conformers (M001–M004) for **5-II** and **6-II**.

26. Conformational search of 1.

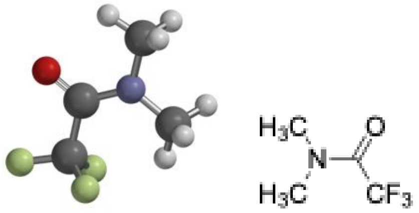

Figure S23. Most stable conformation of **1**.

Table S3. XYZ coordinates of **1** (M001).

Atomic Number		Coordinates (Angstroms)		
		X	Y	Z
1	C	-0.9641293701	-1.7674927317	0.1246891477
2	C	-1.0231519806	-0.2268141314	0.3008065971
3	N	0.0889160163	0.4737721745	-0.0354241083
4	C	0.0867586770	1.9076382815	0.1964944866
5	H	-0.9010422644	2.2079349879	0.5420362521
6	H	0.8338951121	2.1696969294	0.9547215891
7	H	0.3277903464	2.4350790068	-0.7323764042
8	O	-2.0526797212	0.2618218745	0.7241289868
9	F	-0.7809733699	-2.1102384114	-1.1650471749
10	F	-2.0937531894	-2.3173451896	0.5380098793
11	F	0.0458211913	-2.3024487339	0.8359535786
12	C	1.3565224646	-0.0847845827	-0.4773090105
13	H	1.7653440407	0.5623306145	-1.2592584055
14	H	1.2348956027	-1.0793755628	-0.8967163130
15	H	2.0757864444	-0.1297745255	0.3492908989

27. Conformational search of 5-II.

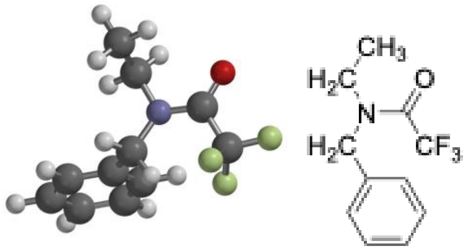

Figure S24. Most stable conformation of **5-II**.

Table S4. XYZ coordinates of **5-II** (M002).

Atomic Number		Coordinates (Angstroms)		
		X	Y	Z
1	C	-3.18048	-0.2272	-0.19977
2	C	-2.03888	-1.23467	-0.49461
3	O	-2.30157	-2.21225	-1.17038
4	N	-0.8119	-0.96601	0.010655
5	C	-0.45698	0.128407	0.909871
6	H	-1.35121	0.683033	1.187384
7	H	-0.06275	-0.30673	1.836946
8	C	0.242495	-1.94501	-0.26596
9	H	0.102928	-2.30846	-1.28533
10	H	1.195021	-1.40991	-0.2243
11	C	0.220981	-3.11134	0.717472
12	H	1.043413	-3.7999	0.503128
13	H	0.331309	-2.76408	1.749632
14	C	0.57228	1.063854	0.309478
15	C	2.461839	2.813915	-0.7808
16	C	1.743755	1.360472	1.002363
17	C	0.356189	1.6478	-0.94051
18	C	1.294982	2.516833	-1.48229
19	C	2.684718	2.234615	0.463195
20	H	3.194727	3.493032	-1.20555
21	H	1.924098	0.903957	1.972959
22	H	-0.55114	1.417833	-1.48978
23	H	1.11591	2.965434	-2.45481
24	H	3.593723	2.456265	1.014009

25	F	-3.46454	-0.18448	1.116246
26	F	-4.27934	-0.58086	-0.84556
27	F	-2.86027	1.02563	-0.58388
28	H	-0.71932	-3.66019	0.63019

28. Conformational search of 5-II.

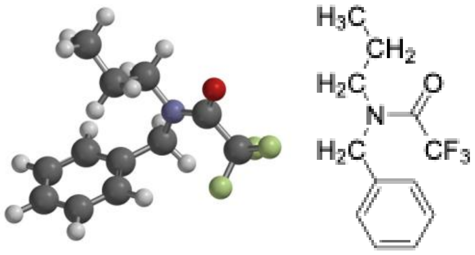

Figure S25. Most stable conformation of **6-II**.

Table S5. XYZ coordinates of **6-II** (M002).

Atomic Number		Coordinates (Angstroms)		
		X	Y	Z
1	C	-3.54199	-0.30514	0.188628
2	C	-2.28691	-1.21609	0.118866
3	O	-2.35031	-2.31271	0.642577
4	N	-1.20035	-0.75256	-0.54351
5	C	-0.05807	-1.66539	-0.63412
6	H	-0.4494	-2.66686	-0.83803
7	H	0.537766	-1.35148	-1.49972
8	C	0.815411	-1.70003	0.620913
9	H	0.187288	-1.98706	1.471655
10	H	1.207041	-0.69768	0.824045
11	C	1.966013	-2.69105	0.460905
12	H	2.586494	-2.71876	1.362246
13	H	1.593123	-3.70616	0.279416
14	H	2.613127	-2.41457	-0.38081
15	C	-1.04943	0.562341	-1.1671
16	H	-0.84127	0.409281	-2.2333
17	H	-1.98812	1.109028	-1.10659
18	C	0.065518	1.369048	-0.53733
19	C	2.150633	2.792073	0.666705
20	C	1.243268	1.62285	-1.23563
21	C	-0.063	1.839386	0.771414
22	C	0.972988	2.544737	1.370793
23	C	2.283684	2.332091	-0.63814
24	H	2.960845	3.341623	1.136747

25	H	1.351647	1.259905	-2.25518
26	H	-0.97466	1.63576	1.325356
27	H	0.863418	2.902523	2.39018
28	H	3.197313	2.522327	-1.19343
29	F	-4.51349	-0.91706	0.845181
30	F	-3.99717	0.003469	-1.03973
31	F	-3.2814	0.856159	0.826996
